# Opisthobranchs from the western Indian Ocean, with descriptions of two new species and ten new records (Mollusca, Gastropoda)

**DOI:** 10.3897/zookeys.197.1728

**Published:** 2012-05-22

**Authors:** Nathalie Yonow

**Affiliations:** 1 Conservation Ecology Research Team, Department of Biosciences, Swansea University, Singleton Park, Swansea SA2 8PP, Wales, UK

**Keywords:** Nudibranchs, Cephalaspidea, Anaspidea, Sacoglossa, Nudibranchia, Doridina, biogeography, taxonomy

## Abstract

Seventy species of opisthobranchs are described in this work based on collections from the Persian Gulf, Socotra, Kenya, Zanzibar, Madagascar, La Réunion, Mauritius, the Seychelles, the Maldives, and Sri Lanka. Ten species are newly recorded from the western Indian Ocean and four species are recorded in the scientific literature for the first time since their original descriptions. Two species are described as new: *Cyerce bourbonica* **sp. n.** from La Réunion and *Doriopsilla nigrocera***sp. n.** from the Persian Gulf coast of Saudi Arabia. *Chromodoris cavae* is removed from its synonymy with *Chromodoris tennentana* and redescribed from specimens from La Réunion, while several new synonyms are proposed for some commonly occurring species. *Risbecia bullockii* is recorded for the second time from the Indian Ocean and assigned to its correct genus.

## Introduction

The year 2010 was designated the *International Year of Biodiversity* by the United Nations; however, biogeographical and taxonomic works cataloguing collections of opisthobranchs are rare in this age of advanced technology and DNA techniques. Expeditions have been mounted in order to collect and record species of particular groups, but systematic papers on their collections often remain unpublished. For example, in 1990 the Rumphius Biohistorical Expedition revisited the area sampled by the Dutch explorer-naturalist Rumphius to compare the marine invertebrate fauna with his records (see Strack 1993, 1998) but the 15^th^ report has only just been published (Yonow 2011). The Chagos Expedition 1996 also concentrated on marine invertebrates and fishes, with conservation aims in mind (Sheppard 1996) but few reports have been published. An expedition to Socotra mounted by the Senckenberg Museum ran from 1997 to 2002; it is ongoing today as a monitoring scheme and two volumes of Fauna of Arabia were dedicated to its findings (2002, volume 19, and 2004, volume 20). The innovative project in Kuwait (http://www.burohappold.com/projects/project/sabah-al-ahmad-sea-city-71/) is already providing a base for coral reef development after only a few years, and is being intensively monitored; the opisthobranch list now approaches two dozen species (Nithyanandan in prep.). Unlike the other localities, most of the Persian Gulf habitats are not typical coral reefs, but patches of coral growing on hard substrata are substantial and support many species.

In this publication, expedition material is supplemented by occasional specimens collected by a number of people from a range of western Indian Ocean localities. Very few of the species were collected in high numbers, but these specimens are none-the-less valuable; if occasional specimens of the uncommon or rare species are never recorded in the scientific literature, our knowledge of the basic ‘data set’ remains hidden, with only a few scientists and museum curators aware of their existence. For example, a description and illustration of an unidentified species from the Maldives based on a single specimen, *Thuridilla* sp. (Yonow 1994), was recognised as new and described one year later (*Thuridilla undula* Gosliner, 1995) as having a widespread Indo-West Pacific distribution: the single Maldive specimen provided the only Indian Ocean record.

Four species included in this report have not been recorded in the scientific literature since their original descriptions, and many other species provide records from new localities. Although some of these new locations may not be unexpected, a surprisingly patchy distribution for opisthobranchs within the Indian Ocean is emerging, with several species occurring only in one or two localities. Ten species are recorded from the western Indian Ocean for the first time: some are common western Pacific species, but several have again been rarely recorded in the literature. This work aims to document some of the species occurring in the region and to provide information on their identities and geographical distributions; not all groups are covered, and subsequent papers will deal with the Aeolidina, Dendronotina, Arminina, and Pleurobranchida as well as provide a more thorough discussion on the biogeography of the western Indian Ocean Opisthobranchia.

A map of the collection localities of the species included in this work is provided below. Species recorded and discussed by the author in previous publications are listed with a relevant and updated synonymy within the Indian Ocean: new specimens are listed with their collection details and brief descriptions of each individual, with colour photographs to provide a visual reference as well as to enable the readers to make their own assessment. Further information acquired on morphology, behaviour, habitat, and distributions is provided, as are references to any recent research. Species not covered in previous papers by the author are described and illustrated in more detail to verify their identification, especially if they have been rarely recorded or are new records for the western Indian Ocean. “The color (both dorsal and ventral) is critically important in nudibranch taxonomy, not only because it provides potentially good diagnostic characters ... Therefore it is important to evaluate color variation among individuals” (Dayrat 2010). With the advent of the internet, sites abound which depict photographs of opisthobranchs. Although some of these sites have been searched for localities and species variability, they are not cited since the records cannot be confirmed, although reference is made to them where they provide confirmation of a distribution. However, the website based in La Réunion includes only specimens and photographs by residents from the islands of Mauritius and La Réunion, and more recently Mayotte; many of these photographs were sent to the author more than fifteen years ago as slides and prints. The records from this website are listed under the relevant species and more information can be found on http://seaslugs.free.fr/nudibranche/a_intro.htm.

**Figure 1.**
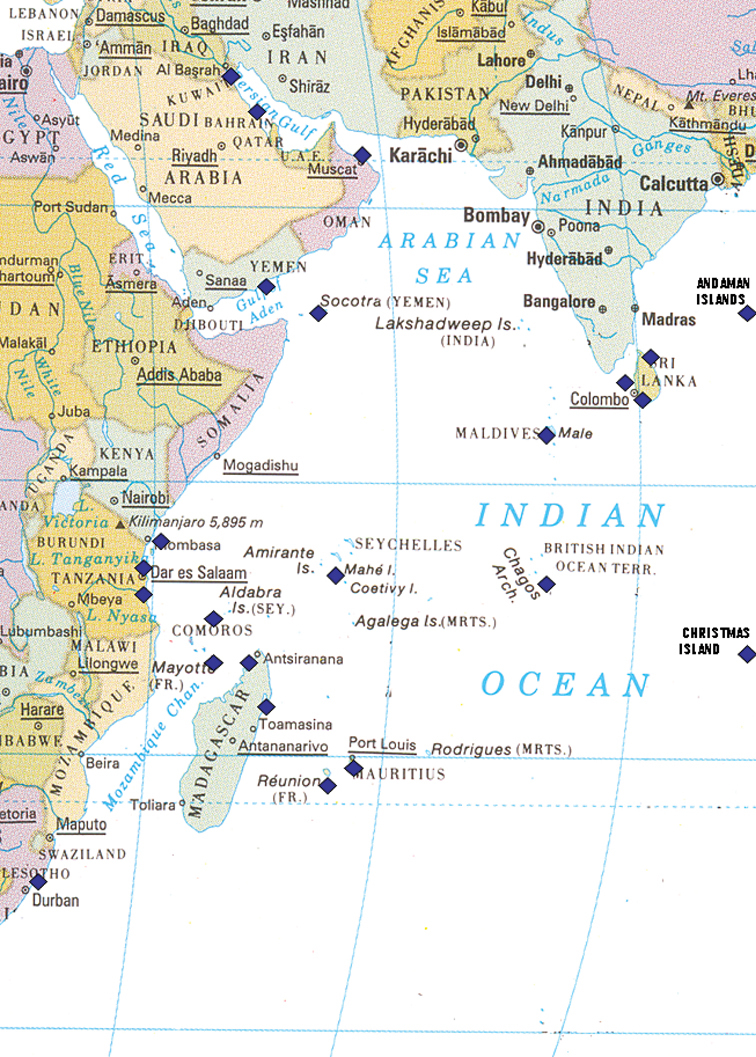
Map of the western half of the Indian Ocean showing collecting localities listed in text marked by blue squares; note the Andaman Islands and Christmas Island are off the map in reality.

## Materials and methods

Parts of these collections have already been reported upon by the author; all methods are as described previously (Yonow 1984, Yonow and Hayward 1991, Yonow 1994, Yonow 1996, Yonow et al. 2002), with specimens preserved in formaldehyde or alcohol; a number of specimens were found unidentified in museums. In all, more than 300 specimens were examined, of which some 250 are included in this paper. All sizes given are living measurements unless otherwise stated in text, figure and plate legends, and all colour and habitat notes made by the collectors are provided in quotation marks in the material section. In some cases, my notes on the preserved specimens are also included, but these are not in quotation marks to differentiate between the two. Photographs listed in the material section are from reliable sources and have been examined carefully; they are listed as individuals to distinguish them from collected specimens. All specimens described in this paper have been deposited in the Senckenberg Museum, Frankfurt, Germany (unless they belong to other institutions) with their radular slides, colour slides, and images. Some specimens had been sent to colleagues for study, and have since been logged into their institutions; these specimens are included as they form part of my unpublished collections, and some provide new records.

The literature was carefully reviewed, and the references included in the synonymies are as complete as possible for Indian Ocean records.

The buccal bulb was removed through ventral longitudinal and horizontal cuts in the head and dissolved in a weak solution of sodium hydroxide (NaOH) for at least 48 hours. The radula was rinsed in several washes of water, stained and flattened on a glass slide with lignin pink in lactophenol, and photographed through an Olympus compound microscope. Dissections of phyllidiids was made by cuts in the mantle enabling the removal of the dorsum from the rhinophores to the anal hole. All line drawings were made using a camera lucida attachment to the Olympus for the radulae, a Wild for the anatomy, or traced from micrographs. All drawings are precisely what was seen and labelled for each specimen, and future workers should be able to link unambiguously all illustrations to the specimens and radular preparations.

## Systematic accounts

### Haminoea cymbalum

(Quoy & Gaimard, 1833) cf.

http://species-id.net/wiki/Haminoea_cf_cymbalum

Plate 1

Haminoea cymbalum . – Rudman 1971: 558, fig. 1 (Fiji, Hawaii); Marshall and Willan 1999: 25 fig. 23 (Great Barrier Reef); Gosliner et al. 2008: 26 (Mozambique, Madagascar + West Pacific); Apte 2009: 165, fig. 1c (Laccadive Islands).

#### Material.

Maldives: two specimens approx. 20 mm length (MDV/AB/96/23), Vilufushi lagoon, Thaa Atoll, 3 m depth, 31 March 1993, leg. RC Anderson & SG Buttress. – La Réunion: photographs of numerous individuals http://seaslugs.free.fr/nudibranche/a_intro.htm.

#### Description.

Globose shell thin, transparent, too small to accommodate body completely; shell 15 mm, living animal up to 25 mm. Body pale green with large and small orange spots, part contained within the shell much darker green than both head and tail. Larger orange spots and patches underlying shell outlined with white or very pale green.

Infrequently, huge mating congregations are observed, with numbers of up to 100 individuals per m^2^ in February 2007 in La Réunion, which lasted approximately one week (P Bidgrain, pers. comm. and http://seaslugs.free.fr/nudibranche/a_intro.htm). These aggregations were not observed again until 2010, but with a much smaller number of animals, only 100 individuals in total (P Bidgrain, pers. comm.).

#### Distribution/Remarks.

*Haminoea cf. cymbalum* has been rarely recorded in the Indian Ocean; it is more common in the western Pacific. Difficulties arise with the name of this well-known species: both Burn (in Marshall and Willan 1999) and Carlson and Hoff (Sea Slug Forum 1999) suggested that the shell representing the type in the MNHN (Paris) is not the same species; therefore this species would take the next available name, which is *Haminoea simillina* (Pease). Additionally, there are several other species differing in colour, radula, and gizzard plates, so further work is needed; deposits of individual specimens such as these in museums with their descriptions and photographs will ensure that enough material is made available for future workers.

### Chelidonura electra

Rudman, 1970

http://species-id.net/wiki/Chelidonura_electra

Plate 2

Chelidonura electra Rudman, 1970: 8, figs. 3-7 (Solomon Islands); Marshall & Willan 1999:18, fig. E (Great Barrier Reef); Gosliner et al. 2008: 44 (Tanzania and Madagascar + W Pacific); Apte 2009: 165, fig. 1d (Laccadive Islands); Richmond 2011: 276 (East Africa).

#### Material.

Maldives: 29 mm length (10 mm pres., MDV/AB/96/1), Guraidhoo Channel, South Malé Atoll, 16 m depth, 30 April 1996, leg. RC Anderson & SG Buttress. – Zanzibar: two pres. specimens 15 mm × 2 mm and 19 mm × 9 mm, Bawe Island, underside of *Porites* head with encrusting *Tubastrea* and sponges, 7 m depth, June 1995, leg. MD Richmond. – Madagascar: 50 mm × 10 mm (PK-C), Ampangorina, Nosy Komba, on algal-encrusted coral, 3 m depth, 30 January 1992, leg. P Kemp. – Mayotte: photographs of two individuals, 15 mm and 25 mm http://seaslugs.free.fr/nudibranche/a_intro.htm.

#### Description.

These specimens and photographs are among the first records of *Chelidonura electra* from the Indian Ocean. *Chelidonura electra* is distinguished by its white body and yellow edges to the parapodia and tails. There are no similar specimens in the Indian Ocean: *Chelidonura pallida* Risbec is found in the western Pacific and eastern Indian Ocean only; it has not been recorded from the western Indian Ocean. Also white, it has a black line along the edges, followed by an inner orange-yellow band. Gosliner (1980) suggested that *Chelidonura electr*a might be a colour form of *Chelidonura pallida*, arguing that colour can be variable in a single species but in his recent book (Gosliner et al. 2008) they are treated as separate species, and their different distributions are acknowledged. They are also treated as different species with different reproductive strategies in Anthes and Michiels (2007). *Chelidonura amoena* Bergh is also found in the eastern Indian Ocean and western Pacific: it is also a large species, translucent white with diffuse yellow margins to the head and tail and dark brown to black speckled pigmentation on the dorsum and parapodia, the latter having yellow anterior and posterior corners (pers. obs., Willan and Cattaneo-Vietti 1995).

### Chelidonura hirundinina

(Quoy & Gaimard, 1833)

http://species-id.net/wiki/Chelidonura_hirundinina

Plate 3

Bulla hirundinina Quoy & Gaimard, 1833: 367, pl. 26, figs. 20-25 (Mauritius).
Chelidonura philinopsis Eliot, 1903a: 336 (Zanzibar); Rudman 1973b: 203, figs. 1, 2, 3D, 5 (Zanzibar).
Chelidonura hirundinina . – Macnae 1962: 194 (South Africa); Gosliner 1987: 42, fig. 11 (South Africa); Gosliner 1988: 90, fig. 9 (Aldabra, Seychelles).

#### Material.

Maldives: three specimens, approx. 5-6 mm length pres., Kuredu, Lhaviyani Atoll, on sandy substrate in shallow water, 10-24 March 1998, leg. J Hinterkircher; several individuals, photos only, 1986-1994, J Hinterkircher. – La Réunion: photographs of numerous individuals, 10 - 40 mm http://seaslugs.free.fr/nudibranche/a_intro.htm.

#### Description/Distribution.

Dark brown to black body with black and orange edging, vivid blue Y- or T-shaped mark on head, small white patch at posterior end of head shield, and large white crescent-shaped mark on posterior shield. The species is rarely recorded in the Indian Ocean, where the pattern is relatively consistent, and somewhat different to those from the Pacific Ocean. Gosliner (1988) comments on the different colour forms, and that two colour forms were recorded mating in South Africa. *Chelidonura hirundinina* is recorded in the Mauritius - La Réunion region, the type locality, and it has a circumtropical distribution (also found in the Caribbean, Thompson 1977). Most records are from the western Pacific, where it is larger, more abundant, and more variable in colour and pattern, although the variations exhibited by http://seaslugs.free.fr/nudibranche/a_intro.htm are substantial.

### Chelidonura punctata

Eliot, 1903

http://species-id.net/wiki/Chelidonura_punctata

Plate 4

Chelidonura hirundinina var.* punctata* Eliot, 1903a: 336, pl. 8, fig. 2 (Zanzibar).
Chelidonura punctata . – Yonow and Hayward 1991: 4, fig. 4F (Mauritius); Yonow et al. 2002: 834, fig. 2a (Chagos); Mangubhai 2007; fig. 1 (Kenya); Apte 2009: 165, fig. 1e (Laccadive Islands).
non Chelidonura punctata . – Yonow 2008: 79 (Gulf of Eilat) (misidentification).

#### Material.

Maldives: 15 mm (MDV/AB/96/15, “very dark brown, almost black, white edge anteriorly and blue grey edge to parapodia, with orange-red spots”), Fulidhoo Channel, Felidhoo Atoll, 9 m depth on coral rock, 7 May 1996, leg. RC Anderson & SG Buttress. – Mauritius, La Réunion, and Mayotte: five individuals photographed http://seaslugs.free.fr/nudibranche/a_intro.htm.

#### Distribution.

**Plate 5.**
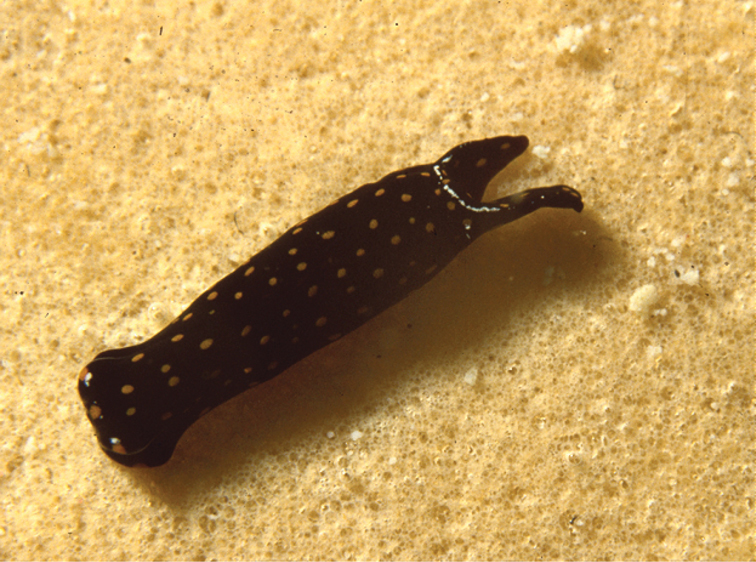
*Chelidonura cf. punctata*, Seychelles, photo: P Kemp (no spcm).

*Chelidonura punctata* appears to be restricted to the western Indian Ocean; to date there are no records of the species occurring in the Red Sea, the Gulf of Aden, the Gulf of Oman, or the Persian Gulf. The similar species found in the Gulf of Eilat (recorded erroneously as *Chelidonura punctata* in Yonow 2008) and in the Seychelles (pers. obs.) is illustrated here as *Chelidonura cf. punctata* (Plate 5) for comparison as no specimens are available; it has many small, yellower spots which appear to be slightly raised, lacks the white margins to the parapodia but has broken white markings along the posterior margin of the posterior shield, and the tails are very differently proportioned. It is also appears to be small, but without specimens, identification is impossible.

#### Remarks.

An interesting paper documenting the feeding habits of *Chelidonura punctata* in Kenya describes the association between it and an acoel flatworm present on the hard coral *Platygyra daedalea* (Mangubhai 2007); neither the sea slug nor the flatworm was observed on other coral species. A similar association was observed between *Chelidonura livida* and *Waminoa cf. brickneri* in the Red Sea (Yonow 2008).

### Chelidonura sandrana

Rudman, 1973

http://species-id.net/wiki/Chelidonura_sandrana

Plates 6 , 7

Chelidonura sandrana Rudman, 1973a: 208, figs. 4, 5 (Zanzibar); Yonow 1994b: 100, figs. 2E, 4A, B (Maldives); Yonow et al. 2002: 835, fig. 2b (Chagos); Yonow 2008: 80 (Gulf of Eilat).
Chelidonura babai Gosliner, 1988: 91, fig. 10 (Aldabra, Seychelles).

#### Material.

Maldives: 3 mm pres., Maayafushi, Ari Atoll, shallow water in lagoon, February 1995, leg. J Hinterkircher; photos of two individuals, 1986-1994, J Hinterkircher; 7 mm (5 mm pres., “all black”), Maayafushi Channel, Ari Atoll, 7-17 m depth on sandy slope, 12 October 1994, leg. RC Anderson & SG Buttress.

#### Remarks.

**Plate 6.**
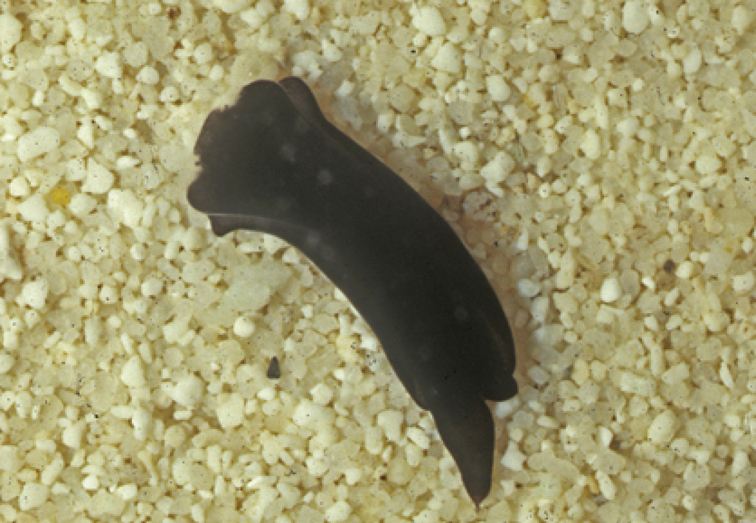
*Chelidonura sandrana*, 7 mm, Maldives, photo: J Hinterkircher.

**Plate 7.**
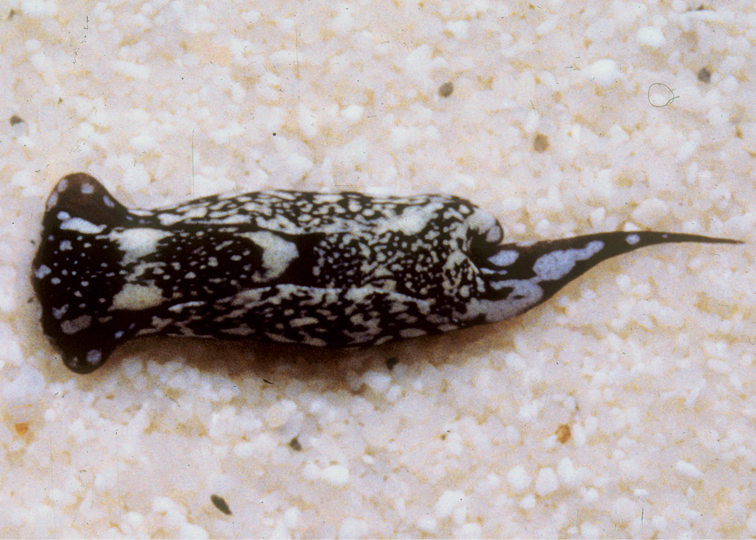
*Chelidonura sandrana*, Maldives, photo: J Hinterkircher (no spcm).

The collecting notes state that there were “plenty of aglajids, all completely black” in Maayafushi lagoon, up to approximately 10 mm in length, and it is very common in the Maldives (Yonow 1994). This species is recorded throughout the western Indian Ocean as well as in the northern Red Sea, and its extensive variability of colouration is now well known; both specimens demonstrate the all-black colouration (Plate 6) while an additional photo (from another locality in the same atoll) depicts an individual which is black with white marbling, but without the orange spots (Plate 7). Turner and Wilson (2011) have shown that the range of colour morphs show no breeding differences; *Chelidonura tsurugensis* Baba & Abe, 1959 may be an older name (Yonow in prep.).

### Chelidonura varians

Eliot, 1903

http://species-id.net/wiki/Chelidonura_varians

Plate 8

Chelidonura varians Eliot, 1903a: 335 (Zanzibar); Yonow 1994a: 102, fig. 4C (Maldives).

#### Material.

Maldives: two specimens, 30 mm and 15 mm pres., Maayafushi Channel, Ari Atoll, 7-17 m depth on sandy slope, 12 October 1994, leg. RC Anderson & SG Buttress.

### Philinopsis speciosa

Pease, 1860

http://species-id.net/wiki/Philinopsis_speciosa

Plate 9

Philinopsis speciosa Pease, 1860: 21 (Hawaii); Gosliner et al. 2008: 29 (Pacific); Yogesh Kumar et al. 2011: 106, fig. 1a (India).
Doridium cyaneum : Eliot 1903a: 334 (Zanzibar).
Aglaja cyanea : Macnae 1962: 193 (South Africa).
Philinopsis cyanea (Martens). – Rudman 1972: 394, fig. 15 (Zanzibar); Gosliner 1987: 41, fig. 9 (South Africa); Yonow 1992: 199, figs. 1, 2 (Gulf of Eilat and Maldives); Yonow 1994a: 103, fig. 2A, 4D (Maldives); Yonow 2008: 81 (Red Sea); Richmond 2011: 276 (East Africa) (syn. n.)

#### Material.

Maldives: two specimens, 18 mm and 12 mm pres., Maayafushi Channel, Ari Atoll, 7-17 m depth on sandy slope, 12 October 1994, leg. RC Anderson & SG Buttress; 24 mm (MDV/AB/96/5), Fulidhoo lagoon, Felidhoo Atoll, 7 m depth, 03 May 1996, leg. RC Anderson & SG Buttress. – La Réunion, photographs of several individuals http://seaslugs.free.fr/nudibranche/a_intro.htm. – Tanzania: photograph of single individual, Mafia Island, shallow water, 31 July 2004, A de Villiers.

#### Distribution/Remarks.

The photographs on the La Réunion website show mating, egg laying, and egg development during the breeding season, November. This common Indo-West Pacific species known as *Philinopsis cyanea*, with an extremely variable colour pattern, has an older name, *Philinopsis speciosa*, which was previously considered as endemic to Hawaii. Rudman (http://www.seaslugforum.net/find/philspec) states that there are no anatomical differences between the two, and Gosliner et al. (2008) have combined them under the older name with no discussion or comment. Pease (1860) described the habit of stressed slugs vomiting perfect shells of *Bulla*, as recorded for specimens from the Red Sea (Yonow 1992) and Zanzibar (Rudman, Sea Slug Forum). *Philinopsis cyanea* is formally synonymised with *Philinopsis speciosa* in this work.

### Aplysia parvula

Guilding in Mörch, 1863

http://species-id.net/wiki/Aplysia_parvula

Plates 10 11

**Figure 2.**
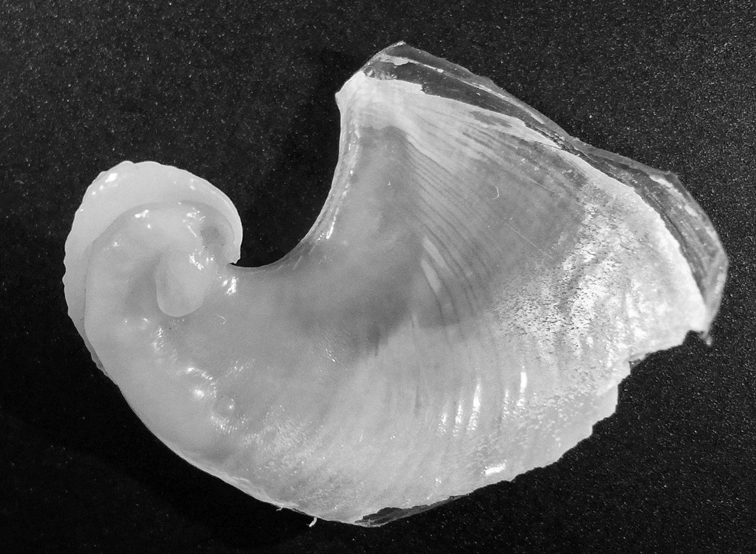
*Dolabella auricularia*, shell of 46 mm specimen (24 mm maximum shell dimension).

**Figure 3.**
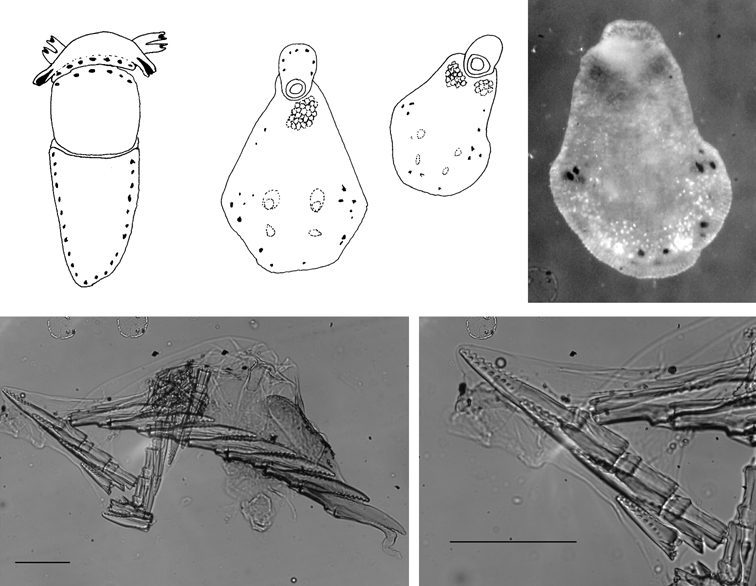
*Cyerce bourbonica*sp. n. **A** ventral view of holotype **B** selection of larger cerata of paratype (formaldehyde) showing morphology: globular patches at base near attachment point, the small flap, and some black pigment spots remaining at the distal end **C** photograph of ceras of paratype (alcohol) from anterior side showing denticulate margin and pointed papillae **D** whole radula of paratype (formaldehyde) **E** single tooth magnified to show denticles and shape of shaft. Scale bars 100 µm.

Aplysia spuria Krauss. – Macnae 1955, 235, figs.1d, 2d, 3d, 6a, b (South Africa).
Aplysia parvula . – Gosliner 1987: 46, fig. 24 (South Africa); Yonow and Hayward 1991: 5, figs. 4E, 5D (Mauritius); Yonow 2008: 98 (Red Sea); Apte 2009: 165, fig. 1h (Laccadive Islands).
Aplysia fasciata . – Yonow 1994a: 104, fig. 4G (Maldives); Yonow 2000: 94, fig. 3, plate 7 (Red Sea) (non *Aplysia fasciata* Poiret).
Aplysia cf. parvula . – Yonow et al. 2002: 837, figs. 2d, 3a, b (Chagos).
Aplysia sp. – Yonow 2008: 100 (Red Sea).

#### Material.

Pale form: Socotra: 10 mm × 5 mm pres. (IT-084, N-171), Rhiy di-Irisal, SE site, 2-8 m depth, 24.II.1999, leg. N Simões. – La Réunion, photographs of numerous individuals http://seaslugs.free.fr/nudibranche/a_intro.htm.

Dark form: Maldives: two specimens approx. 14 mm in length, Hulhulé Island, North Malé Atoll, 12 m depth on orange encrusting sponge on outer reef, 30 July 1995, leg. RC Anderson & SG Buttress (“dark brown with numerous tiny white dots, brown tending to orange, greyish white at margins”); two specimens both 14 mm, Old Shark Point, Thilafalhu Reef, North Malé Atoll, 16 m depth, 18 November 1995, leg. RC Anderson & SG Buttress (“dark brown with numerous fine pale dots; edges of [parapodia] and tentacles pale”); 20 mm (MDV/AB/96/13), Fulidhoo Channel, Felidhoo Atoll, 9 m depth, 07 May 1996, leg. RC Anderson & SG Buttress (“no white spots”). – La Réunion, photographs of several individuals http://seaslugs.free.fr/nudibranche/a_intro.htm. – Tanzania: photo of one individual, Mafia Island, shallow water, May 2009, A de Villiers.

#### Description/Remarks.

**Plate 10.**
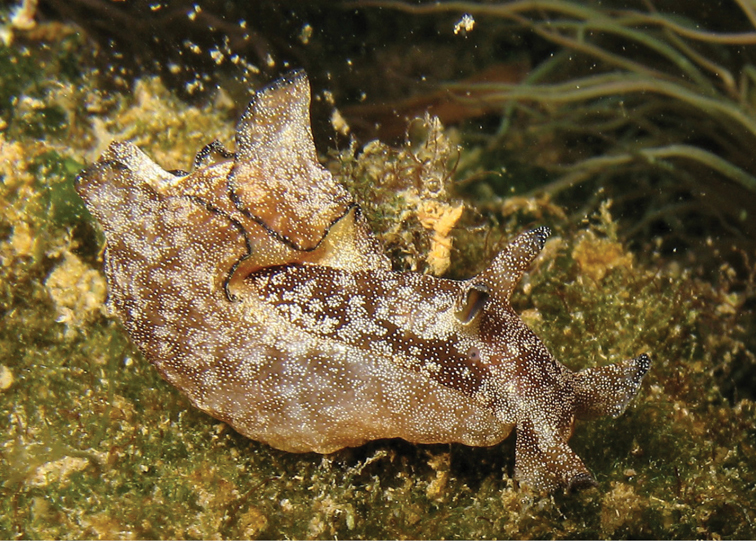
*Aplysia parvula*, 20 mm, La Réunion, photo: P Bidgrain (no spcm).

**Plate 11.**
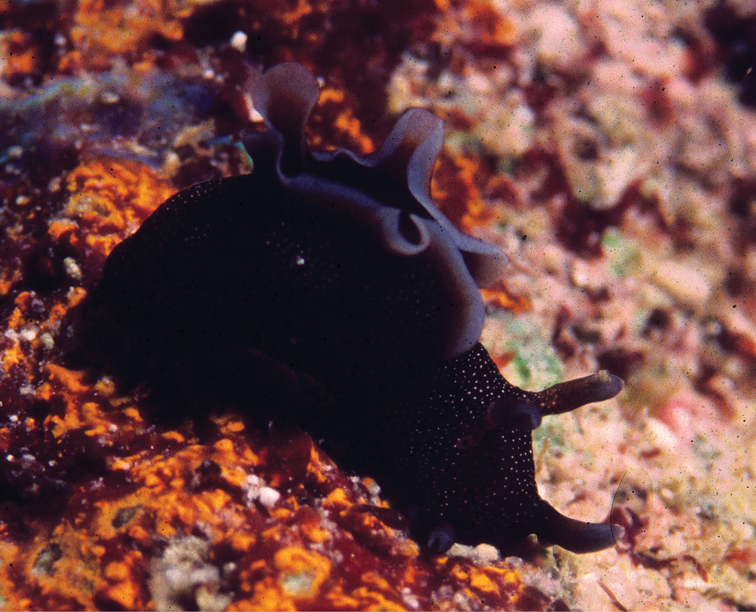
*Aplysia parvula*, 14 mm, Maldives, photo: RC Anderson.

The dark colour form is common in the Red Sea and western Indian Ocean (Yonow 1994a, Yonow et al. 2002, Yonow 2008) and also occurs in the western Pacific (Gosliner et al. 2008), albeit less frequently. The pale Socotra specimen is well relaxed and preserved: the body is cream coloured with black edging to the rhinophores, oral tentacles, foot, and parapodia. Both colour forms are small, and one is the reverse colour pattern of the other. Although pale *Aplysia parvula* are recorded to grow to 120 mm (Marshall and Willan 1999), the largest size recorded for the dark colour formis the Maldives specimen listed above at 20 mm. Consistent differences occur in the colour patterns: *Aplysia parvula* sensu strictu is usually pale green or greenish brown with white marbling and spots, and dark purple-to-black margins on the tentacles and parapodia (Plate 10). The black form is very dark brown or black, sometimes with white speckles, and with pale pink or violet edging to the parapodia and tentacles (Plate 11). The pale form is found in shallow tidal areas with seaweed, while the dark form is found on coral reefs in more exposed areas. Several other colour patterns have been illustrated on the La Réunion website.

The radula of the Socotra specimen has the formula 29 (+2) × 4.7.1.7.4. It is comparable in formula and size to those of the dark form previously examined from the Red Sea (26 (+1) × 4.6.1.6.4: Yonow 2000) and Chagos (26 (+3) × 3.6.1.6.3: Yonow et al. 2002), although it must be stressed that the radulae are not very good diagnostic features for differentiating between species of *Aplysia*. The shell is also similar in both forms but again, it is variable and therefore not a particularly useful character for determining species of aplysiids.

### Dolabella auricularia

(Lightfoot, 1786)

http://species-id.net/wiki/Dolabella_auricularia

Fig. 2 Plate 12

Aplysia rumphii Cuvier. – Rang 1828: 46, pl. 1 (La Réunion).
Aplysia ecaudata Rang 1828: 47, pl. 2 (Papua New Guinea).
Aplysia truncata Rang 1828: 47 (Papua New Guinea).
Aplysia teremidi Rang 1928: 48, pl. 3 figs 1 – 3 (Society Islands).
Aplysia gigas Rang 1828: 48, pl. 3 Fig. 4 (shell only) (“Mer des Indes”; “... this shell differs from the previous species...” transl.) (syn. n.).
Dolabella auricularia . – Bebbington 1974: 73, figs. 7D, E, 8 (Tanzania, Seychelles (Aldabra), Kenya, Zanzibar, Grande Comoro); Gosliner 1987: 48, fig. 28 (South Africa); Yonow and Hayward 1991: 6, figs. 5E, F, 7D, 12A, B (Mauritius); Yonow 1994a: 104, figs. 2B, C, 4H (Maldives); Apte 2009:167: fig. 1j (Laccadive Islands); Richmond 2011: 278 (East Africa).
Dolabella gigas . – Engel 1942: 197, figs. 1-5 (“Indian Ocean” and Mauritius + West Pacific); Eales 1946: 149, figs. 1-8 (Bombay, India) (syn. n.).
Dolabella scapula (Martyn). – Engel 1942: 207, figs. 6-16 (“Indian Ocean,” Mauritius, Mozambique + West Pacific).

#### Material.

Zanzibar: 46 × 28 mm pres., Kizimkazi Dimbani reefs, intertidal, June 1994, leg. Suki/MD Richmond. – La Réunion, Mauritius, and Mayotte: photographs of numerous individuals and two shells http://seaslugs.free.fr/nudibranche/a_intro.htm. – Oman: Muscat, 1-12 April 2009, photo S Kahlbrock. – Seychelles: one individual photographed, Lilôt, NW Mahé, 1988-1989, P Kemp.

#### Description/Remarks.

Examination of the shell (Fig. 2) identifies the Zanzibar specimen as the tropical Indo-Pacific *Dolabella auricularia*; the specimen had rounded tubercles in life, and these remain on the preserved animal. There are only two species of *Dolabella*, but to date no recent specimens of *Dolabella gigas* (Rang) have been recorded. The two species are said to differ in shell morphology and internal characters (Engel 1942, Eales 1946): Bebbington (1974) gave a history of *Dolabella gigas* and *Dolabella auricularia*. Engel (1942) listed both species, with slightly different shell morphologies, long and armed or short and unarmed penises, based on one specimen and 13 shells of *gigas* and many more of *auricularia* (as *scapula*). Eales (1946) examined two specimens of *Dolabella gigas* from India and confirmed the differences in shell morphology but was unable to confirm the presence of spines on the penis.

The radular formulae appear smaller in *gigas* but it is difficult to make size-for-size comparisons from the literature. This study demonstrates that the shells of three specimens from La Réunion and Zanzibar with rounded tubercles, the external morphology of *Dolabella gigas*, prove to be identical to *Dolabella auricularia* (P Bidgrain and M Jay, pers. comm., see http://seaslugs.free.fr/nudibranche/a_intro.htm for shell photos). Looking at the many shells illustrated by Engel (op. cit.) and Eales (op. cit.), there appears to be a clinal variation from one extreme to the other. Smooth and hirsute specimens were recorded together from Mauritius (Yonow and Hayward 1991) and both spiky and warty specimens were recorded from the Maldives (Yonow 1994a); all shells examined were similar, which eliminates the previously divisive external morphologies. It is probable that some of the shells of *Dolabella gigas* illustrated in the literature are extremes or aberrant; there appears to be only one record of penis spines, which remains unconfirmed. If this is considered an error, any differences between the two species are eliminated: it seems extremely unlikely that there would be two very large species of *Dolabella* distributed together in the same regions, and both are here considered the same species.

#### Note.

The Maldives specimen and shell listed in Yonow (1994) are deposited in The Natural History Museum, London (NHMUK 20110444), and not in the Australian Museum, Sydney, as stated in that paper due to subsequent postal security regulations.

### Dolabrifera dolabrifera

(Cuvier, 1817)

http://species-id.net/wiki/Dolabrifera_dolabrifera

Dolabrifera dolabrifera . – Bebbington 1974: 78, figs. 7F, 9 (Tanzania, Seychelles (Aldabra), Kenya, Zanzibar); Yonow and Hayward 1991: 6, fig. 5A-C (Mauritius); Yonow 2008: 102 (Red Sea); Apte 2009: 165, fig. 1i (Laccadive Islands); Richmond 2011: 278 (East Africa).

#### Material.

Socotra: 10 mm curled pres. (St-021, MAP-097), rocks with algae, W of Rhiy di-Diblih, Nogid, S coast, 12 March 1999, leg. M Apel. – Maldives: 60 mm approx. (49 mm pres.), in *Tridacna* culture tank, Marine Research Section, Malé, North Malé Atoll, 30 December 1995, leg. RC Anderson (“mottled olive green; extruded white gelatinous fluid from posterior siphon”); photos only of several individuals, March 1997, March 1998, March 1999, J Hinterkircher. – Mauritius and La Réunion: numerous individuals with various colours and textures http://seaslugs.free.fr/nudibranche/a_intro.htm. – Seychelles: photo of one individual, Lilôt, NW Mahé, 1988-1989, P Kemp.

#### Description/Distribution.

Preserved specimen from the Maldives are relaxed, with rhinophores and tentacles well extended, and a distinct flange present around margin; Socotra specimen very contracted and curled up. Colours of living specimens are extremely variable, and the villi may or may not be present; *Dolabrifera dolabrifera* can grow to 50 mm in length, and is common in the western Indian Ocean and Red Sea in shallow inter- and sub-tidal coastal waters; it has a wide Indo-Pacific distribution.

### Notarchus indicus

Schweigger, 1820

http://species-id.net/wiki/Notarchus_indicus

Notarchus indicus . – Farran 1905: 355 (Sri Lanka); Eales 1944: 12, fig. 12 (Zanzibar); Yonow 2000: 96, pl. 9 (Red Sea); Yonow 2008: 105 (Red Sea).
Notarchus ceylonicus Farran, 1905: 355, pl. 5 figs. 18-23 (Sri Lanka).

#### Material.

Socotra: four specimens 15-20 mm preserved lengths (IT-157, RJ-011), 12°18.698'N, 53°48.285'E, 09 April 1999, leg. R Janssen.

#### Description/Distribution.

Preserved specimens are similar to previously examined material from the Red Sea, severely contracted into spheres with varying amounts of speckling remaining on different specimens. The species is circumtropical, common where it occurs but not often recorded. Records from the western Indian Ocean are summarized by Bebbington (1974); there are no new records apart from those from the Red Sea listed above.

### Stylocheilus longicauda

(Quoy & Gaimard, 1824)

http://species-id.net/wiki/Stylocheilus_longicauda

Plate 13

Aplysia longicauda Quoy & Gaimard, 1824: 421, pl 66 Fig. 8 (New Guinea); Rang 1828: 73, pl. 22 figs 8 - 10 (Quoy & Gaimard specimen, on “*fucus*”).
Aplysia citrina Rang, 1828: 71, pl. 22 figs. 1, 2 (Atlantic Ocean on *Sargassum*).
Aplysia nudata Rang, 1828: 72, pl. 22 figs. 3-5 (Sandwich Islands on “fucus”).
Aplysia striata Quoy & Gaimard, 1832: 315 (New Guinea).
Stylocheilus longicauda . – Bebbington 1974: 87, figs. 7G, 14, 15 (Kenya and Zanzibar); Gosliner 1987: 49, fig. 30 (South Africa); Yonow and Hayward 1991: 5, fig. 3E (Mauritius); Yonow 2008: 104 (Red Sea).
Stylocheilus striatus . – Yonow 2008: 108 (Red Sea); Apte 2009: 167, fig. 1k (Laccadive Islands); Richmond 2011: 278 (East Africa).

#### Material.

Maldives: 6 mm in length pres., 2 m depth on algal-covered stone, Kuredu, Lhaviyani Atoll, 10-24 March 1998, leg. J Hinterkircher; three specimens collected of many, 50-70 mm live lengths (28-32 mm preserved), on algae growing on floating grouper cage near surface, Kiadhu Tila, Felidhoo Atoll, 23 July 1994, leg. RC Anderson (striations still visible on preserved specimens). – Zanzibar: numerous specimens 10-50 mm pres. lengths, Mazzini, intertidal and low water forming chains on sand and rocks in sea grass beds, 09 September 1993, leg. MD Richmond (numerous villi, very long rhinophores and oral tentacles). – Mauritius, La Réunion, Mayotte, and Madagascar: photographs of numerous individuals http://seaslugs.free.fr/nudibranche/a_intro.htm. – Oman: Muscat, 01-12 April 2009, photos of several individuals, S Kahlbrock.

#### Description/Distribution.

Benthic form of this circumglobal species distinguished by fine longitudinal brown-to-black lines usually interrupted by ‘eyespots’ with bright blue centres; tail long, swelling present in middle of body where parapodial lobes enclose large gill and anal siphon; rhinophores and oral tentacles long and slender. Specimens in any given population may have few or numerous villi, and this variation is still visible in the preserved material. It can be locally common in sea grass beds and shallow water, especially during the breeding season, but this does not happen every year (P Bidgrain, pers. comm. and http://seaslugs.free.fr/nudibranche/a_intro.htm).

#### Remarks. 

There is probably only one species of *Stylocheilus* occurring in two forms, the benthic striated form described and illustrated here, which was known as *striatus*, and the yellow pelagic form associated with seaweeds currently known as *longicauda*.

This may be an appropriate location to discuss the problems posed by the aplysiids distributed in the Indo-West Pacific: all species covered in this work are complex and appear to comprise colour forms identified as separate species, as well as having different habitat preferences. *Aplysia parvula* occurs consistently in at least two forms, the larger pale-coloured shallow water one associated with algae and the tiny black reef form although other colour forms are recorded (http://seaslugs.free.fr/nudibranche/a_intro.htm and http://www.nudipixel.net/species/aplysia_parvula/). It is noted that the radulae and shells were similar, with slight differences. *Dolabrifera dolabrifera* has been described under many names but has long been recognised as a single species. Many species of *Dolabella* have been described but were historically resolved to two species, with different external and (possibly) internal morphologies and shells; these are synonymised as one species in this work. The history of *Stylocheilus* is also complicated, with two very different forms inhabiting very different environments. Clearly the aplysiids are in need of revision, entailing comparison of specimens from different regions and habitats as well as a very thorough review of the literature; it is probable that the numerous species names currently listed will dissolve into fewer, variable species.

### Cyerce bourbonica sp. n.

urn:lsid:zoobank.org:act:EF8569BD-D10C-4309-A53B-C1A37F1DEB76

http://species-id.net/wiki/Cyerce_bourbonica

**Plate 14.**
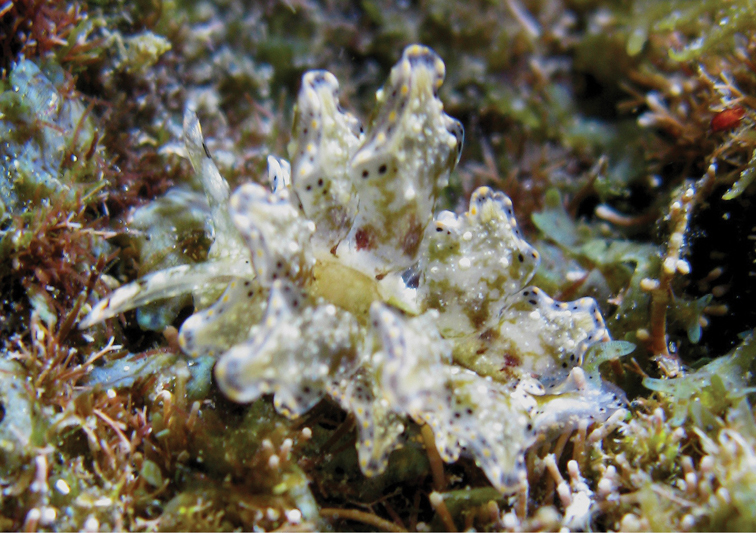
*Cyerce bourbonica* sp. n., 12 mm holotype, La Réunion, photo: H Flodrops.

**Plate 15.**
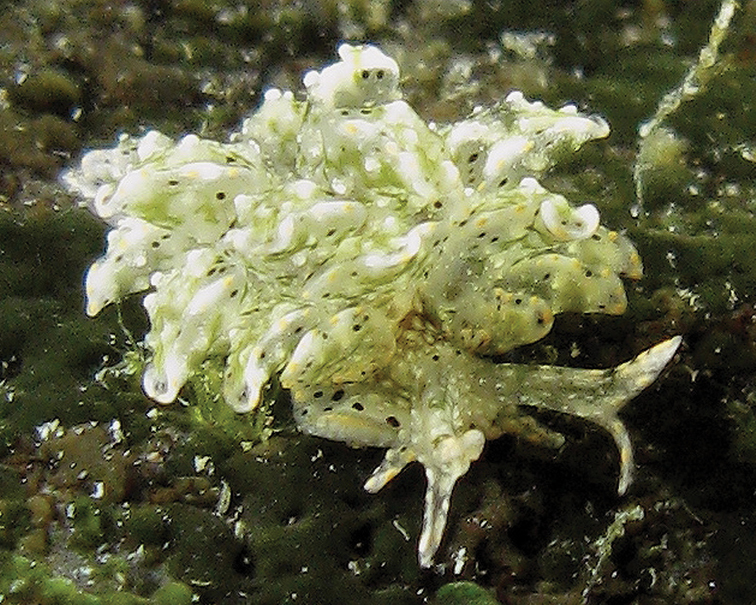
*Cyerce bourbonica* sp. n., 12 mm paratype, La Réunion, photo: H Flodrops.

Fig. 3 Plates 14 , 15

Cyerce sp. 2. – Gosliner et al. 2008: 70 (Tanzania, Madagascar, La Réunion, and Aldabra + Pacific).
Cyerce sp. 3. – http://seaslugs.free.fr/nudibranche/a_intro.htm (La Réunion).
Cyerce sp. – http://www.nudipixel.net/photo/00024541/ (Similan Islands, Thailand).

#### Material.

Holotype: 12 mm alive, 6.5 × 2.5 mm pres. in alcohol, Etang Salé, La Réunion, rocky coast, 1 m depth, 14 December 2009, leg. H. Flodrops, SMF 337104.

Paratypes: approx. 12 mm alive, 4 × 3 mm pres. in formalin (dissected), Etang Salé, La Réunion, rocky coast, 1 m depth, 14 December 2009, leg. H. Flodrops, SMF 337103; 10 mm alive, 4.5 × 2 mm pres. in alcohol, Etang Salé, La Réunion, rocky coast, 1 m depth, 14 December 2009, leg. H. Flodrops, SMF 337205.

#### Diagnosis.

A multi-coloured *Cyerce* with approximately 30 inflated, pustular, angular cerata whose margins are pale violet-blue with creamy orange patches and black spots. The head has an orange band on either side and black spots on its frontal margin. The body and cerata are marbled light green and white. Ventrally, the propodium and metapodium each bear a row of small black dots, the former anteriorly and the latter marginally and posteriorly. Size up to approximately 15–20 mm.

#### Description.

The description of the colour and pattern is based on a series of photographs of the type specimens: body covered in fewer than 30 slightly inflated and tubercular cerata, loosely arranged in transverse rows along each side. Each ceras can be flat or swollen, angular with rounded corners (Fig. 3B, C), semi-translucent beige to green, margins pale blue-violet ‘beaded’ with opaque pale orange pigment, and sub-marginal black spots. The profile is in one plane along the sides but strongly convex at the distal end. Tubercles opaque white, concentrations of larger black spots in lateral swellings. Very few black spots scattered on cerata but yellow spots, olive-green patches, and light green to light brown marbling present. In some photographs, paired dark brown globular patches visible in alcohol-preserved specimens can be seen as circular red-brown patches at bases of cerata (Plate 14, right-hand cerata at level of pericardium). Anterior portion of body marbled: head and rhinophores semi-translucent; eyes clearly visible at bases of rhinophores (Plate 15). Oral tentacles short and recurved. Frontal margin of head with black spots, and behind eyes and rhinophores, coalesced to form a network. Broad orange band on each side of head, outside the black spots. Pericardium immediately behind first two or three rows of cerata, swollen, ochre-coloured, bare of cerata.

The specimens preserved in alcohol are in excellent condition; the paratype in formaldehyde shed 23 larger cerata. It is cream in colour, while the holotype and second paratype are semi-translucent. Dark patch of pigment anterior to swollen pericardium behind rhinophores and faint pigmented patch beyond. Pericardium a large swelling but dissection of paratype in formaldehyde did not reveal vessels and disintegrated with further prodding. Large swollen anal papillae behind right rhinophore near eye in all preserved specimens. Conical penis visible below right rhinophore in alcohol paratype only, no spine apparent at tip. Ventral views well presented in alcohol (Fig. 3A); rhinophores visible, as are head and oral tentacles; propodium with row of black spots along anterior margin, rounded metapodium with a row of black spots all around its edge except anteriorly. Cerata angular with three rounded corners near distal margin. At the base, doughnut-shaped attachment point and small flap beyond (Fig. 3B). Minute pointed papillae located in a band following the contours on both surfaces of the cerata, white in certain angles of light (Fig. 3C). Edges of some preserved cerata appear serrated (Fig. 3C). Brown triangular patch present on flap at base of each ceras in alcohol-preserved specimens. Two dark brown to black bodies inside cerata, formed of very small globules (Fig. 3B,C), near base above and beside attachment point.

Dissection of the formaldehyde-preserved paratype proved impossible as only the outer skin was preserved, the internal organs decomposed. However, the muscular pharynx remained solid and was removed to extract the radula. Radular formula 16 × 0.1.0, nine teeth in the ascending limb and seven teeth in the descending one (fig. 3D). The teeth are typical in shape for *Cyerce* (e.g. Hamatani (1976) for *Cyerce kikutarobabai*), and 170 μm in length. The denticles along the cutting edge are paired, with the first denticles at the tip smaller (fig. 3E). Denticles 1-7 increase in size, while denticles 8-10 decrease, and denticle 11 is just a small bump. The teeth are curved along their long axis and fit closely into the next tooth, the denticles leaving indentations in the underlying shaft. In profile, the shaft is shorter and barely narrower than the cusp (fig. 3D, E).

#### Remarks.

This distinctive species appears to extend throughout the Indo-West Pacific oceans (Gosliner et al. 2008). *Cyerce bourbonica* sp. n. has also been discussed on the Sea Slug Forum based on photographs from Japan, La Réunion, and possibly Hawaii (http://www.seaslugforum.net/find/cyersp3). It is recorded frequently from La Réunion but only from two localities. *Cyerce bourbonica* sp. n. is clearly distinguishable from the named species of *Cyerce* previously recorded from the western Indian Ocean, all three species of which are recorded from Mayotte and/or La Réunion (http://seaslugs.free.fr/nudibranche/a_intro.htm). *Cyerce elegans* Bergh, 1870, is translucent cream to beige (sometimes red) with rounded swollen cerata. The edges of the cerata glisten with faint iridescent scalloped markings and there is usually a blue spot at the base of each. *Cyerce pavonina* Bergh, 1888, has curved swollen cerata covered in pustules on both surfaces and is pale greenish brown in colour, and the outer and inner surfaces of the cerata differ in colour. *Cyerce nigricans* (Pease, 1866) is black and has golden spots and a red band on the cerata.

#### Etymology.

The species is named ‘bourbonica’ after the original name of the island of La Réunion, the type locality. The island was renamed La Réunion in 1793 after the fall of the House of Bourbon (Spain and Luxembourg currently have Bourbon monarchs), but the name was changed back and forth several times until the French revolution in 1848.

### Elysia nigropunctata

(Pease, 1871) cf.

http://species-id.net/wiki/Elysia_cf_nigropunctata

Fig. 4 Plate 16

? Pterogasteron nigropunctatus Pease, 1871: 304, pl. 22 fig. 2 (Tahiti).
Elysia sp. 11. – http://seaslugs.free.fr/nudibranche/a_intro.htm (La Réunion, Mauritius, and Mayotte); http://www.seaslugforum.net/find/elyssp11 (Tanzania, Madagascar, South Africa + W Pacific).
Elysia sp. 12. – http://www.seaslugforum.net/showall/elyssp12 (French Polynesia).
non Elysia nigropunctata . – Bergh 1873: 80, pl. 9 fig. 7, pl. 11 figs. 7-12; Risbec 1928: 284, pl. 12 fig. 9.

#### Material.

Seychelles: 20 × 5 mm (PK-HH, “10 mm when parapodia are opened”), Whale Rock, on coral rubble, 26 April 1992, leg. P Kemp; numerous photographs from La Réunion, Mauritius, and Mayotte http://seaslugs.free.fr/nudibranche/a_intro.htm.

#### Description.

Body pale green with ocellated black spots and orange patches along edges of parapodia where they were thrown into three permanent folds. Rhinophores marbled green with white pigment dorsally, creamy orange submarginal band, and white distal end. Oral tentacles tipped with white.

**Figure 4.**
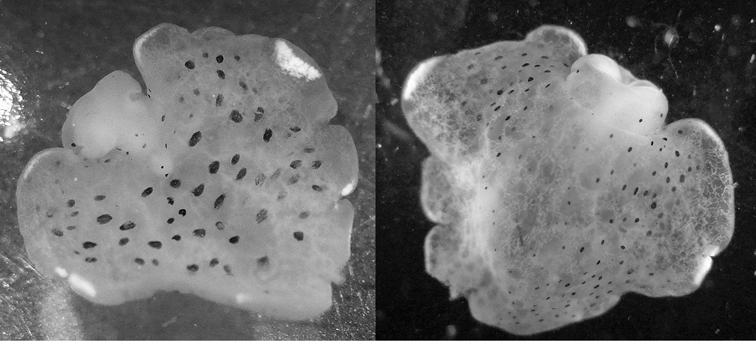
*Elysia cf. nigropunctata*, 20 mm **A** dorsal view of preserved specimen showing irregular parapodial margins and pigmentation **B** ventral view of same specimen.

The single specimen is well preserved, with the parapodia opened completely. Dorsally, there are large black ovate spots (oval along the horizontal axis) on inner surfaces of parapodia and body. Edges of parapodia scalloped in three places, containing opaque white granular pigment spots along the margin (Fig. 4A), presumably the orange marginal pigment in life. The pericardium is elongated, triangular anteriorly and squared posteriorly, with only two un-branched vessels posteriorly. Ventrally, the foot and head are bilobed; there are fewer smaller spots on the foot and more larger spots on the outer surfaces of the parapodia (Fig. 4B).

#### Remarks.

This species is recognisable, as evidenced by the internet discussions (http://www.seaslugforum.net/find/elyssp11) and is most probably Pease’s species, described from Tahiti. The many photographs on the internet show slight variations only, and it is remarkable that it has not been recorded before. This is not only the first record for the Indian Ocean, but the first published record since the species was described in 1861. Another specimen measuring 40 mm from Madagascar is lodged in the Natural History Museum, London (NHMUK 20010521, leg. Lindsey Warren) but was not examined by this author.

### Plakobranchus ocellatus

van Hasselt, 1924

http://species-id.net/wiki/Plakobranchus_ocellatus

Plate 17

Plakobranchus ocellatus . – Yonow 1990: 288, pl. 2 (Red Sea); Apte 2009: 169, fig. 2a (Laccadive Islands); Richmond 2011: 276 (East Africa).
Placobranchus ocellatus . – Rao 1962: 1, figs. 1, 2e, f (India); Jensen 1992: 283, figs. 23, 24d, e (Red Sea and SW Thailand + Guam, Hawaii).

#### Material.

Kenya: 25 × 11 mm preserved, Vipingo, 25 miles N of Mombasa, rock pool ELW, 23 September 1984, leg. J Hognerud. – Maldives: 9 mm in length (MDV/AB/96/7, specimen disintegrated, no radula located), Fulidhoo Lagoon, Felidhoo Atoll, 10 m depth on sand, 04 May 1996, leg. RC Anderson & SG Buttress. – Zanzibar: 14 × 8 mm preserved, Matemwe Lagoon, in *Xenia* sp. and sand near encrusted, partly submerged rock with *Didemnum molle* and *Halimeda* sp., 01 March 1995, leg. MD Richmond. – Seychelles: 30 × 10 mm alive (PK-A, one of four individuals preserved), Source d’Argent, La Digue, 1 m depth on broken *Acropora* sp. behind reef in algal growth, 26 January 1992, leg. P Kemp. – La Réunion and Mayotte: photographs of several individuals http://seaslugs.free.fr/nudibranche/a_intro.htm.

#### Description/Distribution.

*Plakobranchus ocellatus* has been recorded in excess of 35 mm in the Indian Ocean (Rao 1962). The western Indian Ocean specimens examined here conform to the description of Red Sea specimens (Yonow 1990, 2008). Jensen (1992) listed numerous synonyms from the Pacific Ocean, stating that colour pattern and distribution of ocelli are variable in just a single species; however, in 2006 she suggested, “It is possible that a complex of sibling species is involved.”

#### Remarks.

In the Marshall Islands, an interesting commensal association was observed between *Plakobranchus ocellatus* and the sea cucumber *Holothuria atra* (Mercier and Hamel 2005); this is the first report of an opisthobranch occurring on an echinoderm species. The authors provide evidence of a real association between the two animals through a set of well-designed experiments and natural observations. In the Indian Ocean *Holothuria atra* is also a common species, with a similar habitat to *Plakobranchus ocellatus*, but there are no observations of a similar association.

### Thuridilla gracilis

(Risbec, 1928)

http://species-id.net/wiki/Thuridilla_gracilis

Fig. 5 Plates 18 19

Elysia gracilis Risbec, 1928: 278, fig. 93, pl. 10 fig. 5 (New Caledonia).
Thuridilla bayeri Er. Marcus, 1965: 270, figs. 5, 6; Yonow et al. 2002: 835, fig. 2c (Chagos) (syn. n.).
Thuridilla ratna Er. Marcus, 1965: 270, figs. 7, 8; Jensen 1992: 270, figs. 14B, 16A-C, 17A, 18B (syn. n.).
Thuridilla cf.* bayeri*. – Yonow 1994a: 104, figs. 5a, 6a (Maldives).
Thuridilla gracilis . – Apte 2009: 168, fig. 1v (Laccadive Islands).

#### Material.

Seychelles: 15 × 5 mm (PK-P, “stripy black/mauve rhinophores and body, scattered blue spots, orange line edging parapodia”), Lilôt, NW Mahé, 7 m depth on underside of rock, 25 March 1992, leg. P Kemp; 25 × 5 mm (PK-W, “striations along parapodia but with white edge with orange line, vivid blue spots, rhinophores white with longitudinal stripes and brown orange at tips, orange under mouth”), Lilôt, NW Mahé, 20 m depth under rock, 09 April 1992, leg. P Kemp; photo of one individual, Lilôt, NW Mahé, 1988-1989, P Kemp. – Maldives: three pres. specimens 6 mm, 7 mm, and 12 mm. (MDV/AB/96/10), Miyaru Kandhu, Felidhoo Atoll, 05 May 1996, leg. RC Anderson & SG Buttress; 15 mm (MDV/AB/96/12; badly preserved, no radula found), Fulidhoo Channel, Felidhoo Atoll, 10 m on algae, 07 May 1996, leg. RC Anderson & SG Buttress; photos of two individuals, 1986-1994, J Hinterkircher.

#### Description.

**Figure 5.**
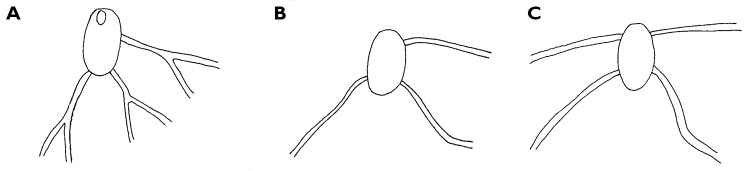
*Thuridilla gracilis*, drawings of pericardia **A** Seychelles 15 mm specimen **B** Maldives 12 mm specimen. **C** Maldives 6 mm specimen.

**Plate 18.**
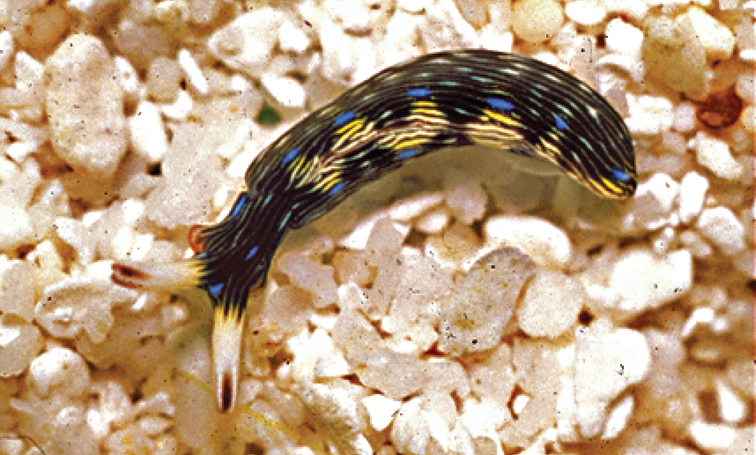
*Thuridilla gracilis* form *bayeri*, Maldives, photo: J Hinterkircher (no spcm).

**Plate 19.**
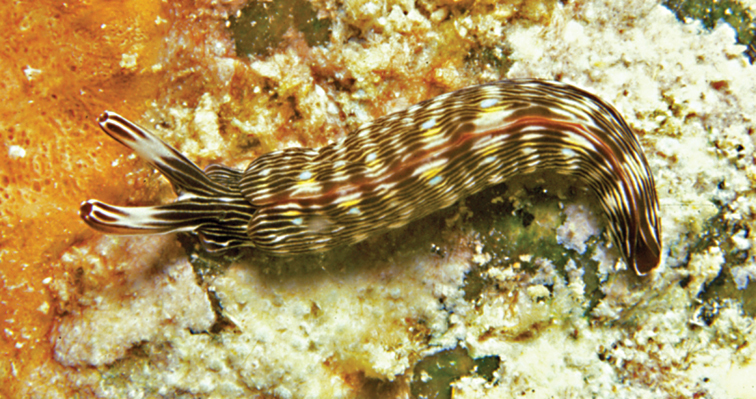
*Thuridilla gracilis* form *ratna*, 25 mm, Seychelles, photo: P Kemp.

Several colour forms exist in the Indo-West Pacific under the names of *bayeri* Er. Marcus (1975) and *ratna* Er. Marcus (1975). Ernst Marcus (1975) named these two species in the same publication based on specimens from the Marshall and Palau Islands: his species were differentiated by the presence or absence of blue spots and the shapes of the radular teeth. As noted in the review by Gosliner (1995), the numerous photographs now available show an overlap in colour pattern; morphologically he thought they had very little to distinguish between them and synonymised *ratna* with *bayeri*. The pericardia of *bayeri* appear to be simpler than those of *ratna*, but Risbec does not describe or illustrate it for *gracilis*. The pericardia of these three specimens are illustrated for information, and are clearly variable (Fig. 5). The sizes of the teeth are also variable, with the range of the teeth of *bayeri* being slightly larger than that of *ratna* (measured from Jensen 1992 and Gosliner 1995). The form with blue spots is the more common in the Indian Ocean, although the form with orange margins is the only one recorded in Mayotte (http://seaslugs.free.fr/nudibranche/a_intro.htm). All the specimens listed above from the Maldives and the Seychelles had bright blue spots in life (Plate 18), and the Seychelles animals additionally had orange margins (Plate 19).

#### Remarks.

*Thuridilla gracilis* (Risbec) was mentioned in a discussion of a new species as being impossible to re-identify (Gosliner 1995), but later, Gosliner (2001) appears to have reconsidered, comparing *splendens* (Baba) with *gracilis*, both species having dark brown to black bodies with cream longitudinal lines and reflective blue pigment spots which may be absent in *gracilis*, and subsequently retaining *gracilis* as a valid species (Gosliner et al. 2008). Risbec’s drawing and description of a 20 mm specimen from New Caledonia is clearly within the cline between *bayeri* and *ratna* in having dark and light green lines on the body and orange tips to the rhinophores, parapodial margins, and oral tentacles. Photographs of many individuals from the type locality, New Caledonia, demonstrate that the specimens occurring at the type locality are well within the extremes shown by *bayeri* and *ratna* (Hervé 2010 and pers. comm.). Risbec’s species is here determined as both recognisable and valid, and the name *Thuridilla gracilis* should take precedence.

### Thuridilla vataae

(Risbec, 1928)

http://species-id.net/wiki/Thuridilla_vataae

Fig. 6 Plate 20

Elysia vataae Risbec, 1928: 281, pl. 12 fig. 7 (New Caledonia).
Elysia vatae . – Gosliner 1987: 53, fig. 43 (South Africa) (misspelling).
Thuridilla vatae . – Jensen 1992: 273 (Western Australia + Guam); Gosliner 1995: 15, figs. 14, 15, 16a (La Réunion + W Pacific); Apte 2009: 169, fig. 1x (Laccadive Islands) (misspellings).

#### Material.

Maldives: approx. 10 mm alive (3 mm pres.), Coral Garden on filamentous green algae, Vadhoo Reef, South Malé Atoll, 5 m depth, 14 October 1994, leg. RC Anderson & SG Buttress; specimen disintegrated (no radula found), 7 mm alive, Bathala Island reef, Ari Atoll, 8 m depth, 28 July 1995, leg. SG Buttress & RC Anderson (“colour dark with pale streaks, rhinophores appeared white with red tips”). – La Réunion and Mayotte: numerous individuals 10-15 mm in length http://seaslugs.free.fr/nudibranche/a_intro.htm.

#### Description.

Ground colour grey with hint of purple, covered with yellow spots and pustules, and round black dots. Marginal bands of the parapodia creamy yellow. Head white, forming X-shape into rhinophores anteriorly and body posteriorly; remainder of head and anterior part of body grey-black. Red-orange band at tip of each rhinophore, a distinctive character of the species; in this specimen, the rhinophores were faintly spotted with creamy yellow.

**Figure 6.**
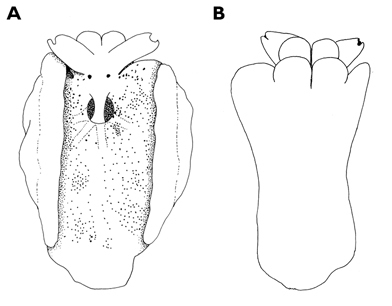
*Thuridilla vataae*, 10 mm (3 mm pres. specimen) **A** dorsal view showing bilobed frontal margin and pericardium with connected vessels **B** ventral view showing bilobed foot and frontal margin.

Preserved specimen semi-translucent with no pigment remaining except black spots on body. Parapodia folded over body and frontal margin bilobed (Fig. 6A). Pericardium unusual in *Thuridilla vataae* in that lateral and posterior vessels are connected. In this specimen, they appear to form a partial ring around the pericardium (Fig. 6A). Ventrally, foot and frontal margin both cleft in preserved specimen (Fig. 6B).

#### Remarks.

*Thuridilla vataae* is a small species recorded sporadically from the Indo-West Pacific. Risbec (1928) almost certainly named this species after the bay south of Nouméa, Baie de l’Anse Vata, of which the north-western tip is the collection locality of his single specimen, Rocher à la Voile. The suffix for commemoratives in this case is *-ae*, hence the spelling *vataae*. The original spelling is correct and should be maintained.

### Nembrotha guttata

Yonow, 1994

http://species-id.net/wiki/Nembrotha_guttata

Plate 21

Nembrotha guttata Yonow, 1994a: 108, figs. 2F, 6E, 8C (Maldives).

#### Material.

Maldives, two specimens, both 35 mm (MDV/AB/96/4), Fulidhoo Channel, Felidhoo Atoll, 9 m depth, 02 May 1996, leg. RC Anderson & SG Buttress; 32 mm (MDV/AB/96/14), Fulidhoo Channel, Felidhoo Atoll, 07 May 1996, leg. RC Anderson & SG Buttress; photo of individual, Maaya Tila, Ari Atoll, 6-8 m depth, March 1994, H Voigtmann.

#### Description.

**Plate 21.**
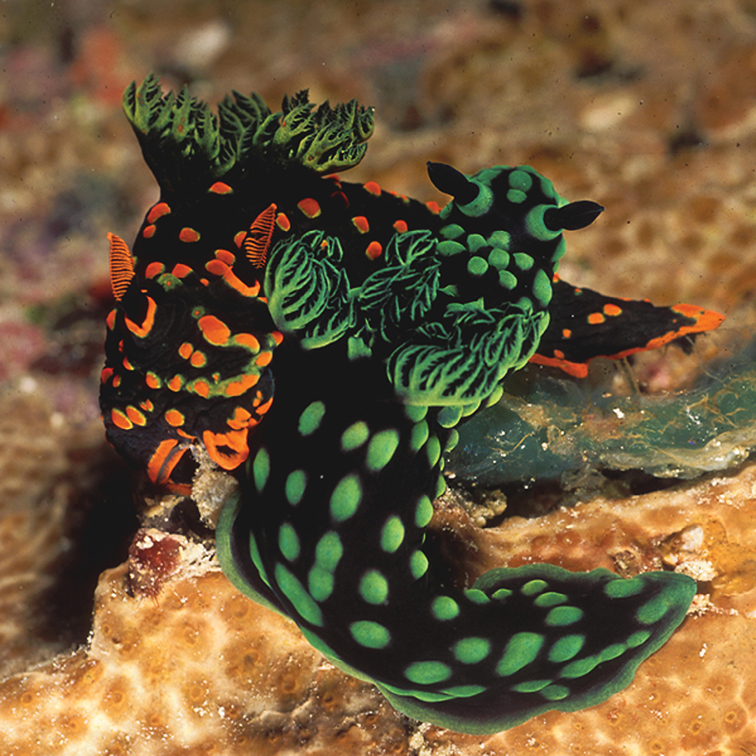
*Nembrotha guttata*, Maldives, photo: H Voigtmann (with *N. cristata*, no spcms).

Easily recognised by velvety black body, large orange pustules edged with green especially along frontal margin, black and orange rhinophores, and green gills. The differences between *Nembrotha guttata* and *Nembrotha cristata* Bergh are clearly visible in Plate 21. *Nembrotha cristata* has been previously recorded from the Indian Ocean, but only in the Maldives (Yonow 1994). A third black and pustulose species known from the region is *Nembrotha kubaryana*, recorded from Mauritius (Yonow and Hayward 1991): it has green pustules but orange gills and rhinophores.

#### Distribution.

*Nembrotha guttata* appears to be endemic to the Maldive islands; paler and consistently differently coloured species are recorded from the Philippines (Pola et al. 2008), Indonesia, Australia, Japan (Gosliner et al. 2008) and New Caledonia (Hervé 2010) but need further investigation. Colour patterns appear to be the best physical characters differentiating species of *Nembrotha*, and the brownish-red spotted pattern with white gills and white pigment between and on the rhinophores currently included in *Nembrotha guttata* is consistent in pattern and colour; it occurs only in the western Pacific Ocean and is most probably a distinct species.

### Roboastra gracilis

(Bergh, 1877)

http://species-id.net/wiki/Roboastra_gracilis

Plate 22

Roboastra gracilis . – Gosliner 1987: 101, fig. 185 (South Africa); Yonow 1994a: 109, fig. 6F (Maldives); Gosliner et al. 2008: 122 (South Africa, Mozambique, Saudi Arabia, and Maldives + Pacific).

#### Material.

Maldives: 8 mm (6 mm pres.), Fulidhoo Channel, Felidhoo Atoll, 12 m depth, 24 July 1994, leg. RC Anderson & SG Buttress; photographs of two individuals 12 mm and 20 mm, March 1999, J Hinterkircher. – Tanzania: photographs of a single individual, M’Nazi Bay, Msimbati, near Mtwara, May 1994, IM Horsfall. – La Réunion: several individuals photographed up to 18 mm in length (http://seaslugs.free.fr/nudibranche/a_intro.htm).

### Tambja amakusana

Baba, 1967

http://species-id.net/wiki/Tambja_amakusana

Plate 23

Tambja amakusana Baba, 1967: 15, fig. 3 (Japan); Marshall and Willan 1999: 56, fig. 87 (Great Barrier Reef); Pola et al. 2006: 510, figs. 10F, 13D, 15A-F (Japan, Papua New Guinea).

#### Material.

Maldives: 11 mm (6 × 2 mm pres.), Old Shark Point, Thilafalhu reef, south end of North Malé Atoll, 10 m depth, 14 August 1995, leg. RC Anderson & SG Buttress.

#### Description.

Animal dark to medium green with some relatively large white spots. Tail extremely long, almost half body length, and body appears to have been smooth. Five small multi-pinnate gills beige with purple tips; rhinophores beige suffused with purple; margins of rhinophore sheaths pale yellow. Flattened oral tentacles visible when crawling.

**Plate 23.**
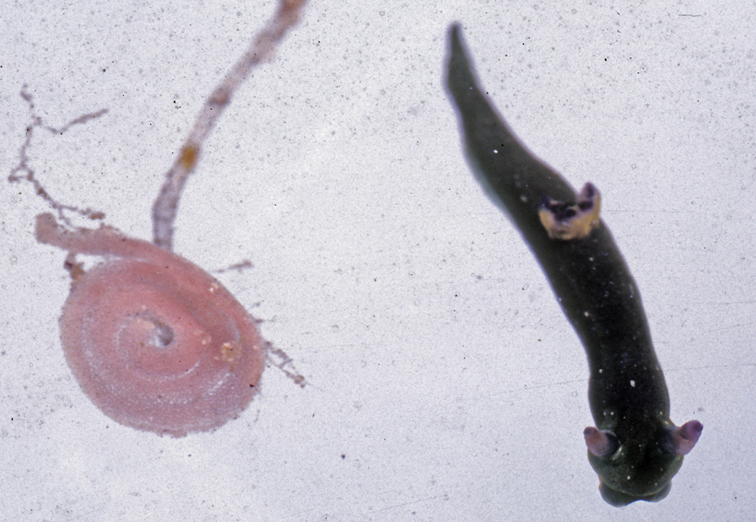
*Tambja amakusana*, 11 mm with egg mass, Maldives, photo: RC Anderson.

The preserved specimen is well relaxed: body light blue-green, gills and rhinophores white, rhinophores with 12 lamellae. Marshall and Willan (1990) state that there is no rhinophoral sheath; none is visible on the specimen but the rim of the right rhinophore is visible in Plate 23 where it catches the light. Baba (1967) described and illustrated the genital opening just below the right rhinophore whereas Pola et al. (2006) located it halfway between the gills and the rhinophore; in this specimen, an orange puckered pore also occurs halfway. The collectors noted that the animal laid a light pink spiral egg mass 4 mm in diameter its second night in captivity; it has barely more than three complete whorls laid close together (Plate 23).

#### Distribution.

*Tambja amakusana* is recorded for the first time from the Indian Ocean; all literature records to date are listed in the synonymy. The only similarly small species with purple tips to the gills and rhinophores is *Tambja limaciformis* (Eliot), but this species is usually brick to orange red, with many more white spots, occurring throughout the tropical Indo-West Pacific (e.g. Yonow 2008).

### Tambja morosa

(Bergh, 1877)

http://species-id.net/wiki/Tambja_morosa

Nembrotha morosa Bergh, 1877: 457 (Philippines).
Trevelyana morosa Bergh, 1873: pl. 25 fig. 9, pl. 33 fig. 7 (Philippines).
Tambja kushimotoensis Baba. – Yonow and Hayward 1991:12, figs. 7F, 12D (Mauritius).
Tambja morosa . – Gosliner 1987: 100, fig. 183 (South Africa); Pola et al. 2006: 493, figs.1A, B, I, fig. 2 (Tanzania + West Pacific).

#### Material. 

Mauritius: 22 × 8 × 10 mm, Japan Wreck, 25 m depth, 04 March 1990, leg. H Debelius and (poor) photograph of two mating individuals; photographs of three individuals http://seaslugs.free.fr/nudibranche/a_intro.htm.

#### Description/Distribution.

Preserved specimen translucent but retains blue pigment in creases on tail, gills, and rhinophores. The species was previously recorded from Mauritius by the author, and appears to be an infrequently encountered species in the western Indian Ocean (see NudiPixel for three records from India, Kenya, and Madagascar).

### Notodoris gardineri nigerrima

Yonow, 1994

Plate 24

Notodoris gardineri var. *nigerrima* Yonow, 1994a: 106, figs. 6C, 8A, B (Maldives); Debelius and Kuiter 2007: 93 (Maldives).

#### Material.

Maldives: 40 mm (35 × 7 mm pres.), Banana Reef, North Malé Atoll, 10 m depth on yellow sponge, night dive, 28 August 1993, leg. RC Anderson & SG Buttress; one individual photographed/described but not preserved, 63 mm (MDV/AB/96/16), Fulidhoo Channel, Felidhoo Atoll, 10 m depth on bare coral rock, 08 May 1996, RC Anderson & SG Buttress; photographs of one individual, 1986-1994, J Hinterkircher.

#### Remarks.

This dark colour form of *Notodoris gardineri* appears to be endemic to the Maldives island group (only one photo from more than 50 available on http://www.nudipixel.net/species/aegires_gardineri/ for example). Otherwise, *Notodoris gardineri* is common in and restricted to the Pacific, with no records from the western Indian Ocean other than form *nigerrima* from the Maldives.

### Notodoris minor

Eliot, 1904

http://species-id.net/wiki/Notodoris_minor

Plate 25

Notodoris minor Eliot, 1904b: 84, pl. 3 fig. 1 (Zanzibar); Yonow et al. 2002: 842, fig. 2f (Chagos).

#### Material.

Socotra: 40 × 13 mm preserved (IT-157, RJ-011), 12°18.698'N, 53°48.285'E, 09 April 1999, leg. R Janssen. – Tanzania: photographs of two individuals, Mafia Island, shallow water, May 2009, A de Villiers. – La Réunion, Rodriguez, and Mauritius: several individuals photographed http://seaslugs.free.fr/nudibranche/a_intro.htm. – Seychelles: one photographed individual, Lilôt, NW Mahé, 1988-1989, P Kemp.

### Hexabranchus sanguineus

(Rüppell & Leuckart, 1828)

http://species-id.net/wiki/Hexabranchus_sanguineus

Plates 26 , 27

Hexabranchus sanguineus . – Yonow & Hayward 1991: 15, fig. 3D (Mauritius); Valdés 2002: 291, figs. 1A, C, 2-4 (South Africa, Mozambique Channel, Madagascar, Philippines, Hawaii) incl. extensive synonymy.
Hexabranchus flammulatus (Quoy & Gaimard). – Narayanan 1968: 378, fig. 2 (India).
Hexabranchus marginatus (Quoy & Gaimard). – Edmunds 1971: 340 (Tanzania); Richmond 2011: 280 (East Africa).
Hexabranchus sp. – Rudman 1986: 347, figs. 1, 19, 20 (Tanzania and Christmas Island).

#### Material.

Socotra: 55 mm pres. length (WPU Rostock, MAR 85, RC-N57), Hadibo, late 1980s, leg. W Wranik. – Yemen: 85 × 60 mm pres., distorted and flattened (WPU Rostock, MAR 85, RC-N16), Al Mukalla, late 1980s, leg. W Wranik. – Kenya: two specimens 60 and 95 mm in length, pres., Vipingo, 25 m N of Mombasa, ELW in rock pools on exposed reef, 23 September 1984, leg. J Hognerud. – Tanzania: photographs of one individual, M’Nazi Bay, Msimbati, near Mtwara, May 1994, IM Horsfall. – Maldives: one juvenile specimen 13 mm (MDV/AB/96/17), Yacht Tila, South Malé Atoll, 25 m depth, 09 May 1996, leg. RC Anderson & SG Buttress; photos of 15 mm juvenile individual, 1986-1994, J Hinterkircher. – La Réunion, Mauritius, Mayotte: photographs of numerous individuals, including juveniles similar to the Maldives specimen examined here (http://seaslugs.free.fr/nudibranche/a_intro.htm) and slides of 27 individuals varying from yellow to mottled pink, M Parmantier. – Seychelles: photos of several individuals including juvenile and sub-adult, Lilôt, NW Mahé, 1988-1989, P Kemp. – Sri Lanka: photographs of one large individual, Unawatuna, S of Galle, on sediment-covered rock, 27 December 2011, S Kahlbrock.

#### Description.

**Plate 26.**
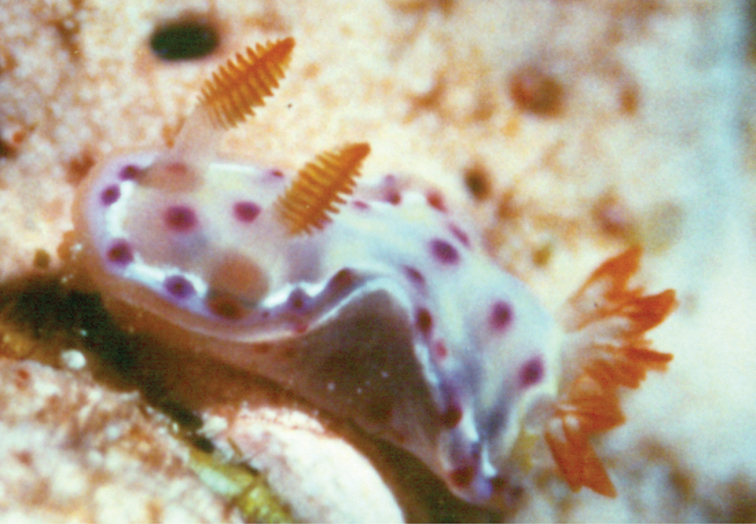
*Hexabranchus sanguineus*, juvenile 13 mm, Maldives, photo: RC Anderson.

**Plate 27.**
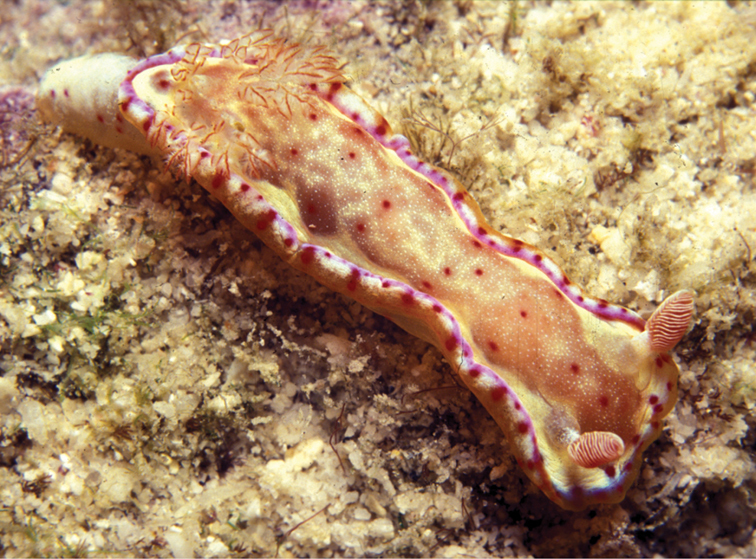
*Hexabranchus sanguineus*, sub-adult, Seychelles, photo: P Kemp (no spcm).

The large specimens and individuals were of the typical Indo-West Pacific colour pattern, blotchy red and cream, especially along the margins. The 13 mm juvenile specimen from the Maldives illustrated here (Plate 26) is well relaxed, and the six gills can be seen to insert into separate openings. The oral tentacles are large rounded lobes, clearly the precursors to the lappets of the adult: these tentacles are huge in comparison to those of a chromodorid (or dorid) of similar size. The rhinophores have 13 lamellae; note that the white edges present in the adults have not yet developed. Another 15 mm juvenile individual was identical in colour pattern, while a slightly larger animal from the Seychelles demonstrated the developing adult colour pattern already had white edges to the 17+ rhinophore lamellae (Plate 27).

The radular formulae of the larger specimens are tabulated below: the sizes listed are of the same dimension of a large lateral tooth – from the tip of the cusp to the flange where the cusp meets the base. There is no relationship between the radular formula and maximum tooth size, nor are they correlated with preserved animal size. A giant Hong Kong specimen is included for comparison, and has the largest teeth but not the largest radula. It had a bubbly texture and was pinkish yellow in life (M Collard pers. comm.; specimen, radula, notes and photographs lodged in the Natural History Museum, London: NHMUK acc. no. 2337 with the “Red Sea Giant”).

 Socotra 55 mm pres. 41 × 64.0.64 500 μm

 Yemen 85 mm pres. 53 × 93-83.0.83-93 550 μm

 Kenya 90 mm pres. 47 × 77.0.77 700 μm

 Kenya 95 mm pres. 46 × 79.0.79 450 μm

 Hong Kong 190 mm pres. 51 × 67.0.67 800 μm

#### Remarks.

Yonow (2001) suggested that the uniformly red species in the Red Sea should be assigned to *Hexabranchus sanguineus* (Rüppell & Leuckart), and that it was distinct from the widespread Indo-Pacific *Hexabranchus marginatus* (Quoy & Gaimard). The study mentioned in that paper was never published, since the following year a paper analyzing the same problem was published by Valdés (2002): Valdés examined material from the western Indian Ocean and the Pacific, but no Red Sea specimens were included in his analysis, nor were any ‘giants’ but, despite this, he concluded that all Indo-Pacific species were the same, and that only the Caribbean species *Hexabranchus morsomus* Ev. Marcus & Er. Marcus was distinct.

### Carminodoris grandiflora

(Pease, 1860)

http://species-id.net/wiki/Carminodoris_grandiflora

Fig. 7 Plate 28

Hoplodoris grandiflora . – Fahey & Gosliner 2003: 170, figs. 1-16 (La Réunion, Madagascar, and Tanzania + W Pacific); Yonow 2008: 158 (Red Sea).
Carminodoris grandiflora . – Dayrat 2010: 184 (in discussion of *Asteronotus raripilosa*).

#### Material.

Zanzibar: 30 mm pres., Matemwe, in sponge, lagoon on east coast, 01 March 1995, leg. MD Richmond. – La Réunion: photographs of one individual, 1990s, M Jay, pers. comm. and http://seaslugs.free.fr/nudibranche/a_intro.htm.

#### Description.

Numerous photographs of living specimen depict an oblong animal slightly broader posteriorly, with pinkish beige gill clump spanning width of animal. Darker central patch extended from just in front of rhinophores to gills, very dark brown where the tubercles were not ringed by white but lighter along the midline where seven large and numerous small tubercles were ringed in white. Many tubercles “frosted” with white pigment, which appears to be a characteristic of living specimens of *Hoplodoris grandiflora*. Remainder of mantle consisted of paler band of beige tubercles outlined in white; extended white areas formed patches perpendicular to the margin.

**Figure 7.**
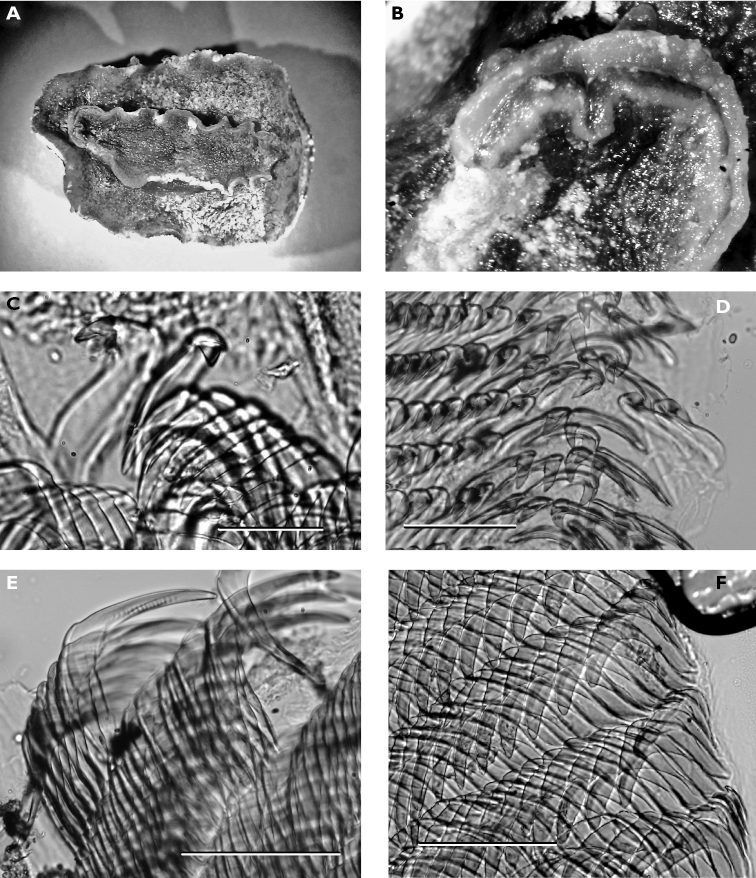
*Carminodoris grandiflora*, 30 mm **A** ventral view of preserved specimen showing small tubercles on hyponotum **B** view of propodium, head, and tentacles showing notched bilaminate foot and damaged on right side **C** median pair of radular teeth, new row, scale bar 50 µm **D** central lateral teeth, scale bar 100 µm. **E** middle lateral teeth showing denticles on cusp and apical projection, scale bar 150 µm **F** outer lateral teeth showing rapid reduction in cusp size, scale bar 150 µm.

Dorsally the preserved specimen still bears distinct hard beige and brown tubercles. The central ones are the largest, and tubercles decrease in size towards the margins. The six gills are multipinnate, and the sheath is scalloped. The rhinophores extend from a raised irregular sheath bearing pinkish beige tubercles. The ventral surfaces of both sole and hyponotum are creamy beige with light brown pigment in the creases, lighter than dorsal tubercles (Fig. 7A). There are small pustules covering the hyponotum but not the sole. The foot is bilaminate for the entire anterior margin extending laterally for a short distance down each side, both laminae are notched, and the oral tentacles are triangular (Fig. 7B).

The radula of the 30 mm preserved specimen has the formula 38-39 × 107.0.107. The first lateral on each side has a long base and a short triangular cusp with a small denticle on each side (Fig. 7C). The remaining teeth have an equally long base with a small flange, but the cusp increases in length (Fig. 7D) and develops serrations along the side (Fig. 7E). The last two or three teeth in each row are very reduced (Fig. 7F).

#### Distribution.

This species has a widespread distribution in the western Indian Ocean, the Red Sea (Yonow 2008), and the western Pacific (Fahey and Gosliner 2003).

### Halgerda formosa

Bergh, 1880

http://species-id.net/wiki/Halgerda_formosa

Plate 29

Halgerda formosa . – Yonow & Hayward 1991: 18, fig. 9 (Mauritius); Fahey and Gosliner 1999: 366, figs. 1A-B, 2, 3A (Tanzania); Debelius and Kuiter 2007: 235 (La Réunion).
Halgerda punctata . – Rudman 1978: 67, figs. 3A, 7, 8 (Zanzibar) (non *Halgerda punctata* Farran).
Halgerda punctata . – Gosliner 1987: 69, fig. 90 (South Africa) (non *Halgerda punctata* Farran).
non Halgerda formosa . – Gosliner 1987: 69, fig. 89 (South Africa) (= *Halgerda dichromis* Fahey & Gosliner).

#### Material.

Zanzibar: 10 × 5 mm pres., leg. MD Richmond (California Academy of Sciences, CASIZ 169953). – Kenya: photographs of two individuals, Malindi National Park & Watamu National Park, 5-8 m depth, MD Richmond. – La Réunion, Mauritius, and Mayotte: photographs of numerous individuals http://seaslugs.free.fr/nudibranche/a_intro.htm.

#### Description.

Body colour translucent white, yellow to orange-yellow ridges, and several dark spots anteriorly and posteriorly near mantle edge. Foot translucent white with at least one black spot near tip and broad pale yellow margin. Rhinophores long, translucent white with one dark brown spot on stalk; yellow on proximal third, dark brown on distal two-thirds of lamellate portion, white on elongated tip. Six gills white with black band across inner side of each rachis from base to tips of pinnules.

#### Distribution.

This small species was first recognised by Yonow and Hayward (1991), based upon specimens collected from Mauritius and La Réunion, the type locality. Three specimens from Western Australia (Fahey and Gosliner 2001) extend the range of this species to the eastern Indian Ocean.

### Halgerda punctata

Farran, 1905

http://species-id.net/wiki/Halgerda_punctata

Fig. 8 Plate 30

Halgerda punctata Farran, 1905: 339, pl. 3 figs. 4-7 (Sri Lanka); Debelius and Kuiter 2007: 232 (Sri Lanka); Gosliner et al. 2008: 177 (Sri Lanka).
non Halgerda punctata . – Rudman 1978: 67, figs. 3A, 7, 8 (Zanzibar) (= *Halgerda formosa* Bergh).
non Halgerda punctata . – Gosliner 1987: 69, fig. 90 (South Africa) (= *Halgerda formosa* Bergh).

#### Material.

Sri Lanka: three pres. specimens 9 × 5 mm, 17 × 10 mm, 20 × 12 mm, and two pres. curled 15 × 10 mm, 15 × 8 mm, Unawatuna, S of Galle, 27-30 December 2010, leg. S Kahlbrock.

#### Description.

The photographs accompanying these five specimens from the type locality are all clearly of the same species: body semi-translucent white with numerous yellow-capped tubercles and few round black spots, tubercles in some specimens arranged in lines approximating ridges of other species of *Halgerda*. Rhinophores long, tapering; long translucent stalk with black pigment on its posterior surface continuing to lamellate portion, which was black with black knob at tip. First 4-6 lamellae yellow on anterior face and remaining 20-24 lamellae black on all sides. Black spot always present at base of rhinophoral sheath posteriorly in line with black line on stalk, often another black spot laterally or anteriorly. At least one yellow tubercle on rhinophoral pocket rim. The gills distinctive in this species with only four gill branches. Anterior two gills larger than posterior two, inner rachides marked with brown-black line. The anal papilla protruded in some photographs of living specimens, and was identical to Farran’s description of “rather long and tubular, white with a black crenulated margin.”

**Figure 8.**
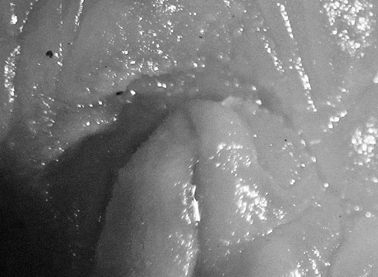
*Halgerda punctata*, 15 mm pres. specimen, ventral view showing head and very reduced oral tentacles.

**Plate 30.**
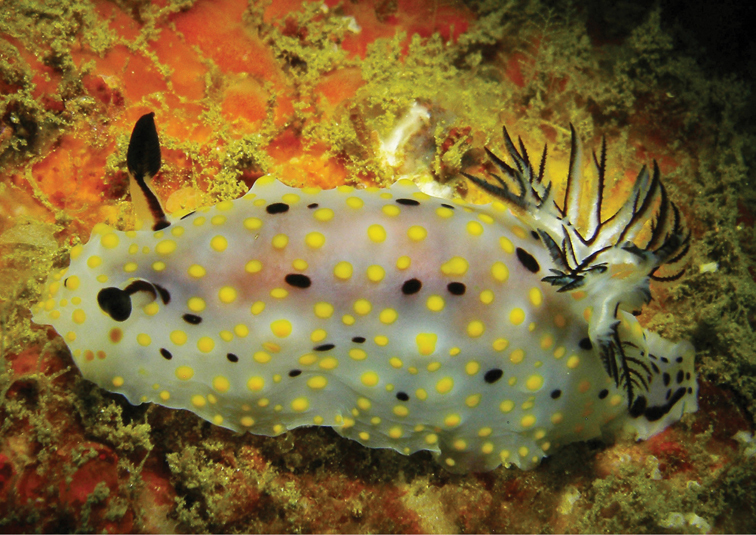
*Halgerda punctata*, 17–20 mm pres., Sri Lanka, photo: S Kahlbrock.

The preserved specimens are firm, grey-white with pale yellow spots on low tubercles in all but one specimen, which has pale orange spots. All specimens have round black spots. The rhinophoral pockets have a posterior black spot on the rim extending to the mantle on all specimens and an anterior black spot in four specimens. There are black spots on the top of the metapodium, which also has an orange spot at the tip fading into a line along the middle. The branchial pocket is faintly tuberculate and there is always a large round or oval black spot placed anteriorly on the mantle in each specimen, also visible in Plate 30. The gills, retain their black and white markings. The hyponotum is narrow with a few small spots. There are larger spots on the sides of the foot but the sole is bare. The head is reduced, and the mouth is visible with very small rounded tentacles (Fig. 8).

#### Distribution.

This appears to be the first record in the scientific literature of this species since its description. It was originally described from Sri Lanka, and most photographic records are from the type locality, with a few from Thailand and Burma to the east (NudiPixel and Sea Slug Forum). The species described as *Halgerda punctata* by Rudman (1978) and Gosliner (1987) were re-identified as *Halgerda formosa* by Fahey and Gosliner (1999), so it appears no one has found or redescribed Farran’s species.

### Halgerda tessellata

(Bergh, 1880)

http://species-id.net/wiki/Halgerda_tessellata

Dictyodoris tessellata . – Eliot 1905: 229 (Madagascar).
Halgerda tessellata . – Rudman 1978: 65, figs. 4C, D, 6 (Kenya); Debelius and Kuiter 2007: 236 (Maldives, South Africa); Apte 2009: 171, fig. 2p (Laccadive Islands).
Halgerda tesselata . – Yonow et al. 2002: 844, fig. 11a (Chagos) (misspelling).

#### Material.

Kenya: two specimens 15 × 15 mm and 9 × 5 mm, Vipingo, 25 miles N of Mombasa, rock pools at ELW, 23 September 1984, leg. J Hognerud (California Academy of Sciences CASIZ 169945). – Tanzania: photograph of single individual, Mafia Island, shallow water, May 2009, A de Villiers. – Maldives: two specimens 15 mm and 25 mm (MDV/AB/96/11), Foteo Channel, Felidhoo Atoll, 15 m depth, 05 May 1996, leg. RC Anderson & SG Buttress (California Academy of Sciences CASIZ 169996); photos of two individuals, 1986-1994, J Hinterkircher. – Mayotte: photographs of two individuals http://seaslugs.free.fr/nudibranche/a_intro.htm. – Seychelles: photos of two individuals, Lilôt, NW Mahé, 1988-1989, P Kemp.

#### Description.

Easily recognised by pattern of orange ridges enclosing brown depressions speckled with white. Both specimens from Kenya were examined when they were received in 1984: the 15 mm specimen was still yellow-orange, as was the foot; the brown reticulated pattern also remained. Very few spots were present on hyponotum but between the hyponotum and foot, there was a band of brown dots/patches. The smaller specimen retained an orange edge to the mantle and the foot: the dorsum was yellow-orange with the brown pattern remaining; there were more spots on the hyponotum than in the larger animal, and there were spots and patches present in a band between the hyponotum and the foot. The preserved Maldives specimens were also clearly recognizable several years after collection, with the brown reticulated pattern remaining between the ridges as well as the brown spots scattered on the hyponotum.

#### Distribution.

*Halgerda tessellata* has a wide Indo-Pacific distribution although, like some species with such a large range, it has a consistently different colour form in the Pacific: a band of white dots is present around the margin and there is dense white pigmentation on the gills (Carlson and Hoff 1993; Yonow in prep.).

### Cadlinella ornatissima

(Risbec, 1928)

http://species-id.net/wiki/Cadlinella_ornatissima

Fig. 9 Plate 31

Cadlina ornatissima Risbec, 1928: 163, fig. 47, pl. 8 fig. 4 (New Caledonia).
Cadlinella ornatissima . – Rudman 1984: 247, figs. 1, 8, 95-97 (Tanzania and Heron Island, Australia); Yonow et al. 2002: 850, fig. 11c (Chagos).

#### Material.

Sri Lanka: 13 × 6 mm pres., Unawatuna, S of Galle, SW coast, 28 December 2010, leg. S Kahlbrock. – Gulf of Oman: photos of two individuals, Muscat, Oman, 01-12 April 2009, S Kahlbrock. – La Réunion and Mayotte http://seaslugs.free.fr/nudibranche/a_intro.htm.

#### Description.

The collected specimen was deep yellow in life with a white foot, long white rhinophores, and five white pinnate gills. The tubercles were elongated, and some were rounded whilst others were somewhat pointed; they were yellow basally and pink-red distally.

**Figure 9.**
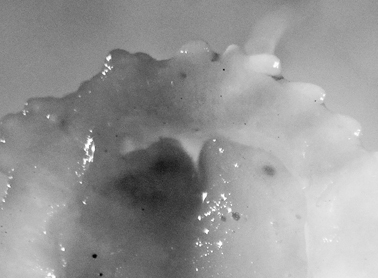
*Cadlinella ornatissima*, 13 mm pres. specimen, ventral view of propodium showing divided foot margin.

The specimen is well preserved, with some yellow remaining on the dorsum several months after collection. There is a tubercle in front of and behind each rhinophore, and three tubercles located just in front of the gill pocket. The spicules on the dorsum and in the tubercles are visible, as are the yellow tubercle bases; the distal parts are now white. Ventrally the hyponotum has an orange submarginal band and the submarginal bands of mantle glands are visible. The foot is deeply divided anteriorly, and the oral tentacles are so contracted as to be non-existent (Fig. 9).

#### Distribution.

This appears to be only the third record of *Cadlinella ornatissima* in the scientific literature from the Indian Ocean, in addition to photographic records from the Gulf of Eilat (Yonow 2008).

### Ceratosoma miamirana

(Bergh, 1875)

http://species-id.net/wiki/Ceratosoma_miamirana

Plate 32

Fracassa tuberculosa Eliot, 1903b: 371 (Zanzibar).
Orodoris miamirana . – Vayssière 1912: 25, pl. 5 figs. 71-77, pl. 6 figs. 87, 88 (Djibouti, Gulf of Aden); Yonow 2008: 207 (Red Sea).
Ceratosoma miamirana . – Valdés and Gosliner 1999: 37, figs. 1D, 4, 5 (Japan, Philippines, Papua New Guinea); Debelius and Kuiter 2007: 103 (Maldives).

#### Material.

Zanzibar: 36 × 34 mm pres., in *Sargassum* off reef crest, Matemwe Lagoon, 01 March 1995, leg. MD Richmond (examined 1995: dorsum cream with rusty/orange-brown patches and pigment in creases, knobbly; rhinophores very close together, located far anteriorly; gills located far posteriorly, pocket with six lappets; ventrally with bright rusty ‘rings’ of pigment on hyponotum) (examined 1998: no pigment left except faint marks in creases and tucked-in gills; rings still bright on hyponotum). – Tanzania: photograph of two individuals, Mafia Island, shallow water, 31 December 2003 and 16 October 2005, A de Villiers. – Maldives: 65 mm (47 × 44 mm preserved, MDV/AB/96/19), 13 m depth, Yacht Tila, South Malé Atoll, 09 May 1996 (examined 1998: very well extended, very soft, completely bleached: no marks or colour left; morphology of foot, oral tentacles, and dorsum as above), leg. RC Anderson & SG Buttress. – La Réunion: photographs of several individuals http://seaslugs.free.fr/nudibranche/a_intro.htm.

#### Description/Distribution.

Recognised by its oval shape with scalloped margin, and blurred green, beige, and blue colour pattern. Ventrally, green reticulations and ocelli located in junction between foot and mantle are distinctive. Widespread if infrequently recorded in the tropical Indo-West Pacific.

### Chromodoris africana

Eliot, 1904

http://species-id.net/wiki/Chromodoris_africana

Plate 33

Chromodoris africana Eliot, 1904a: 392, pl. 24, fig. 4 (Zanzibar); Rudman 1977: 372, pl. 1A, figs. 12C, D, 13, 17C, 18D (Tanzania); Gosliner 1987: 74, fig. 105 (South Africa); Yonow 1989: 293, pl. 1 (Red Sea); Richmond 2011: 280 (East Africa).

#### Material.

Kenya: 25 mm in length, preserved, Vipingo, 25 m N of Mombasa, ELW in rock pools on exposed reef, 23 September 1984, leg. J Hognerud (Australian Museum, Sydney, C431128**)**. – Madagascar: 50 × 20 mm (PK-F), Ampangorina, Nosy Komba, 3 m depth in live *Acropora*, 02 March 1992, leg. and photos P Kemp; photographs of two individuals, Nosy Bé, October 2007, J Hinterkircher. – Tanzania: photographs of two individuals 1994 and Pegasus Wreck, 10 m depth, March 1995, MD Richmond; photographs of two individuals, M’Nazi Bay, Msimbati, near Mtwara, May 1994 and May 1995, IM Horsfall.

#### Description/Distribution.

A large species, growing to 80 mm in length. Ground colour black, two thick or thin white lines running length of dorsum and joined behind gills forming a loop, not extending to pale orange mantle border anteriorly or posteriorly in any of these specimens but may do so anteriorly in the species. Broad, somewhat crumpled, pale orange tending-to-yellow mantle skirt, separated from the black dorsum by a broad white line. No white line at the margin, visible on the preserved specimen from Madagascar which retains its black, white, and orange bands fifteen years after collection. Gills and rhinophores darker orange than border, arising from pockets with raised orange rims. This species has been recorded from the Red Sea and south along the East African coast only (Yonow 2008); surprisingly, it is not recorded from the Gulfs of Aden or Oman, which show close faunal affinities with the Red Sea.

### Chromodoris annulata

Eliot, 1904

http://species-id.net/wiki/Chromodoris_annulata

Plates 34 , 35

Chromodoris annulata Eliot, 1904a: 389, pl. 24, figs. 1-3 (Zanzibar); Edmunds 1971: 371, fig. 15 (Tanzania); Rudman 1973a: 189, pl. 1A (Zanzibar); Gosliner 1987: 74, fig. 106 (South Africa); Yonow 1989: 293, pl. 2 (Red Sea); Debelius and Kuiter 2007: 145 (Oman and South Africa); Richmond 2011: 280 (East Africa).

#### Material.

Persian Gulf: photograph of one individual, Dahwat ad Daffi, Jubail, Saudi Arabia, April 1992, F Krupp; photographs of numerous individuals, 2000-2011, GT Smith; photos of several individuals, Qaruh Island, Kuwait, 2008-2011, M Nithyandanan. – Gulf of Oman: photos of two individuals, Muscat, Oman, 01-12 April 2009, S Kahlbrock; photographs of one individual less than 10 mm, Inchcape 2 (a ship wreck), 20 m depth, 08 January 2010, GT Smith; two individuals, approx. 15 mm, Sharm Rock, 09 January 2010, GT Smith. – Mayotte and La Réunion: photographs of numerous individuals http://seaslugs.free.fr/nudibranche/a_intro.htm.

#### Description.

**Plate 34.**
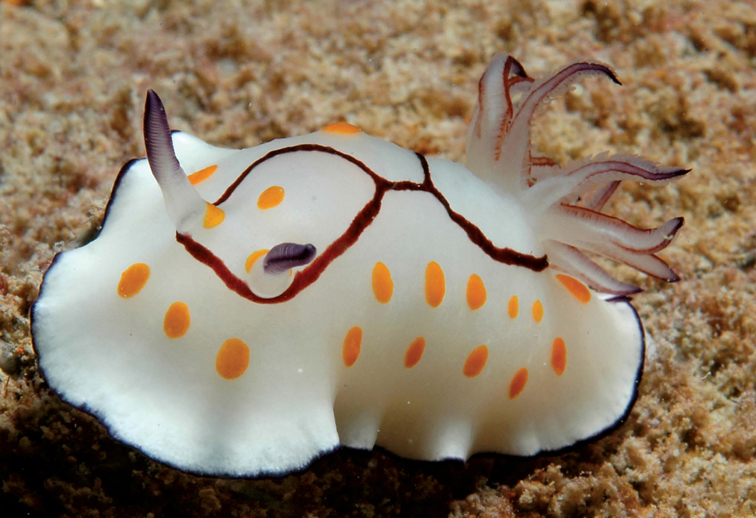
*Chromodoris annulata*, Persian Gulf, photo: G Smith (no spcm).

**Plate 35.**
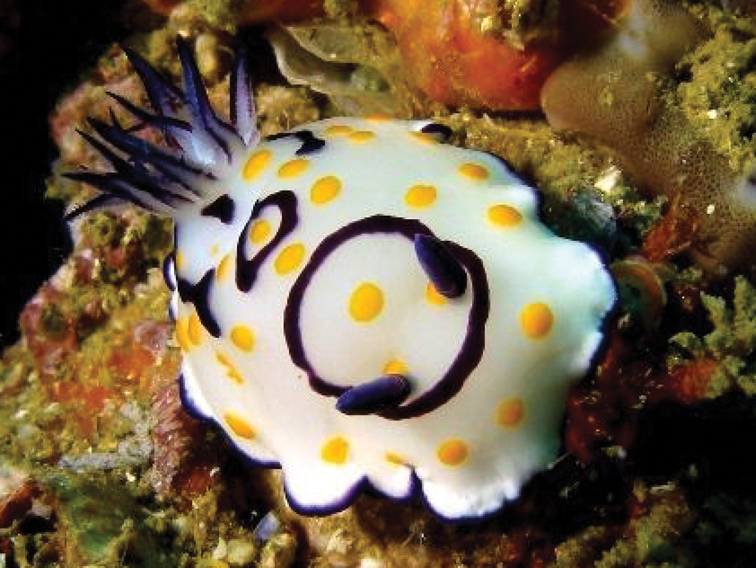
*Chromodoris annulata*, Persian Gulf, photo: S Kahlbrock (no spcm).

Easily recognised, not to be confused with any other species found in the western Indian Ocean and Red Sea. Large and fleshy with raised, medium-sized bright orange pustules, deep purple rings around rhinophores and gills joined by broken line in the Dahwat ad Daffi individual and the majority of photographs of individuals from the Persian Gulf (Plate 34). In the Gulf, the purple rings are not perfect as in the Red Sea, presenting with gaps, dots, dashes, and extra markings (Plate 35). Mantle margin is of the same purple pigment, foot without purple margin but does have few orange spots on its upper surface; rhinophores and gills purple.

#### Distribution.

*Chromodoris annulata* is known from the Red Sea (Yonow 2008) and the east African coastline to South Africa, and is recorded from the Persian Gulf, the Gulf of Oman, La Réunion, and Mayotte for the first time.

### Chromodoris boucheti

Rudman, 1982

http://species-id.net/wiki/Chromodoris_boucheti

Plate 36

**Figure 10.**
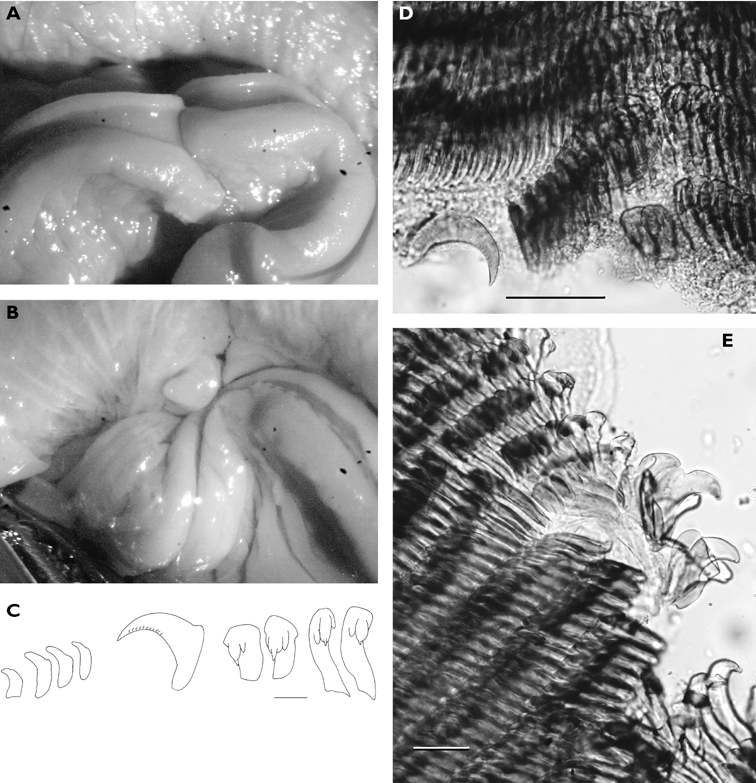
*Chromodoris cavae* **A** ventral view of 76 mm specimen showing bilaminate propodium **B** ventral view of 75 mm specimen showing conical oral tentacles. Radular teeth of 76 mm specimen **C** first two lateral teeth on each side of median, lateral 16, and last 4 teeth (from new rows), scale bar 40 µm **D** central lateral teeth of first row and one displaced early middle lateral tooth showing denticles on cusp, scale bar 100µm **E** outer lateral teeth from an old row showing reduction in cusp, scale bar 100 µm.

**Figure 11.**
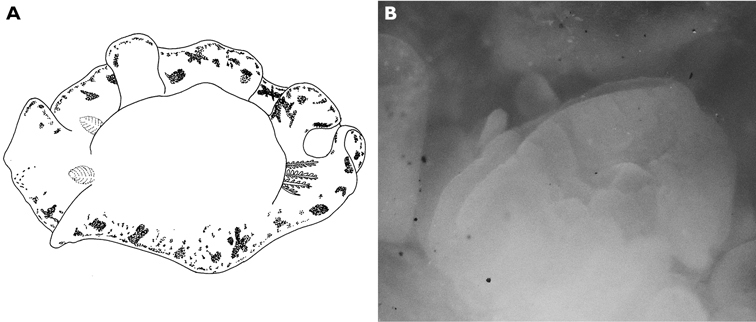
*Chromodoris tennentana*, 8 mm pres. specimen **A** drawing showing mantle glands located in a band around the mantle skirt **B** photograph of bilaminate propodium and oral tentacles.

Chromodoris boucheti Rudman, 1982: 190, figs. 1B, 4, 5 (Mayotte); Yonow 1994a: 110, figs. 10A, 11B-E, J (Maldives): Debelius and Kuiter 2007: 176 (Maldives, Kenya, South Africa).
Chromodoris sp. – Yonow 1994a, 111, figs. 10B, 11A, F-I (Maldives).

#### Material.

Maldives: 30 mm, Bathala Island, Ari Atoll, 28 July 1995, 10 m depth, leg. RC Anderson & SG Buttress (“colour duller and darker than usual”); photographs of several individuals incl. one 5 mm juvenile, March 1997, March 1998, March 1999, J Hinterkircher. – Tanzania: photo of one individual, Mafia Island, shallow water, May 2009, A de Villiers.

#### Description/Distribution.

Pale blue body with three black lines, black sub-marginal line forming a ring, paler blue to white margin; white and orange rhinophores and six gills with distinctive black marks at base on both sides. There may be a general orange wash dorsally. Recent published records of *Chromodoris boucheti* are only from the Maldive Islands and Mayotte: *Chromodoris boucheti* was not found in the Chagos archipelago despite similar sampling techniques, nor has it been recorded from Mauritius, and there is only one record from La Réunion (http://seaslugs.free.fr/nudibranche/a_intro.htm). However, photographs on websites show individuals from Kenya, Tanzania, and South Africa, and this extended distribution is supported by a photograph from Mafia Island, Tanzania (A de Villiers, pers. comm.).

### Chromodoris cavae

Eliot, 1904

http://species-id.net/wiki/Chromodoris_cavae

**Plate 37.**
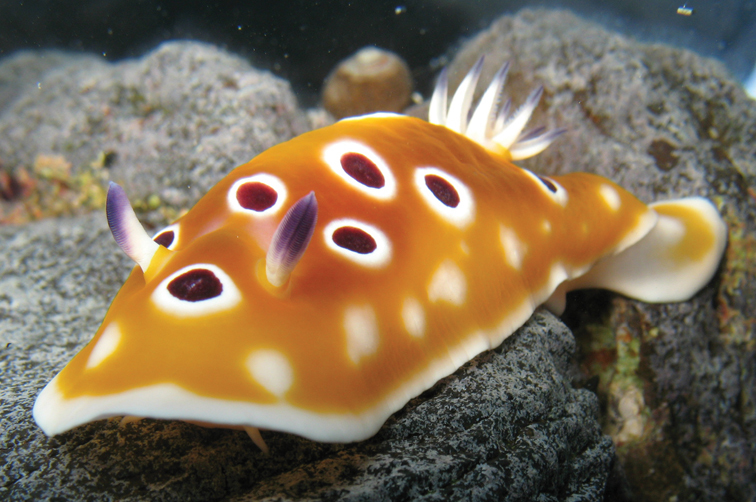
*Chromodoris cavae*, 75 mm, La Réunion, photo: P Bidgrain.

**Plate 38.**
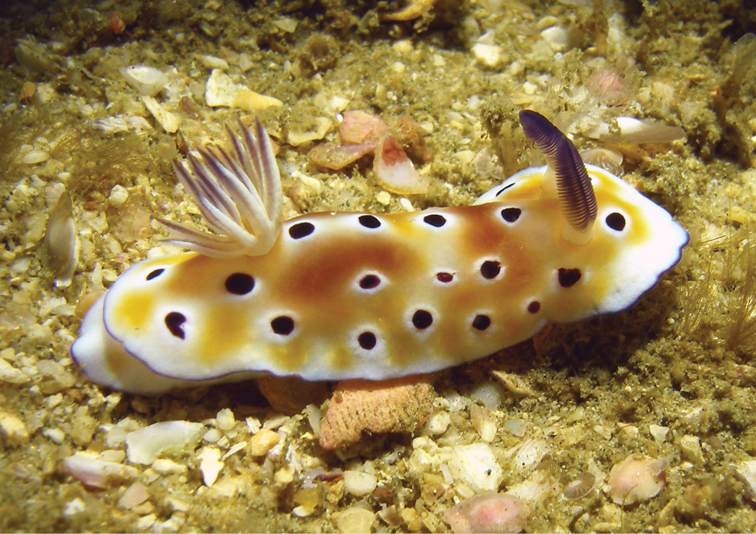
*Chromodoris cavae*, Sri Lanka, photo: S Kahlbrock (no spcm).

Fig. 10 Plates 37 , 38

Chromodoris cavae Eliot, 1904a: 388, pl. 23 figs. 7, 8 (Zanzibar): Debelius and Kuiter 2007: 141 (South Africa).
Chromodoris vicina Eliot. – Gosliner 1987: 75, fig. 109 (South Africa) (non *Chromodoris tennentana* Kelaart).
Chromodoris cf. *leopardus* Rudman. – Yonow et al. 2002: 854, figs. 4f, 10b, 12C, D, 13b (Chagos).
Chromodoris sp. 1. – Debelius & Kuiter 2007: 141 (Oman, Kenya, Mozambique, South Africa).
Chromodoris leoparda (sic. in error for *leopardus*). – Debelius & Kuiter 2007: 141 (Oman, Thailand: middle left and lower two photos only).

#### Material.

La Réunion: 60 mm (25 × 15 mm pres., #1), Bassin des Hirondelles, St. Gilles les Bains, 05 November 2006, 1-2 m depth, leg. P Bidgrain; 76 mm (37 × 20 mm pres., #2), Bassin des Hirondelles, St. Gilles les Bains, 17 September 2009, 1-2 m depth, leg. P Bidgrain; 75 mm (34 × 17 mm pres., #3), Bassin des Hirondelles, St. Gilles les Bains, 17 September 2009, 1-2 m depth, leg. P Bidgrain [all in permanent shallow pool on granite coast]; numerous photographs as *Chromodoris cf. geminus* on http://seaslugs.free.fr/nudibranche/a_intro.htm. – Sri Lanka: photographs of two individuals, Unawatuna, S of Galle and Negombo, N of Colombo, 30 December 2010 and 12 January 2011, S Kahlbrock.

#### Description.

The three specimens are well preserved, accompanied by 10-20 photographs each. They were all similar in life, with an ochre dorsum extending towards a white band around the edge of the mantle; the purple margin normally present in the species was lacking in all three. The first specimen (60 mm) was rusty orange, deeper along central dorsum and fading toward edge; creamy white band along margin but no purple edge. Some photographs are of the hyponotum as the animal flapped its margin, and there was no purple crescent or line ventrally. Six large wine-red patches with white annulus outside and white speckles inside. Around edge of rust-coloured area was a ring of small round wine red spots, each also with white ring. Rhinophores white, distal part of frontal surfaces and tips purple: 35 lamellae on right rhinophore, very faint purple wash at tips remaining on preserved specimen. 25 pinnate gills arranged with two ends of an arc spiralling inwards: each is triangular, flat surface white and translucent pinnae on other two surfaces. Both branchial and rhinophoral pockets slightly raised, orange in life. Foot and digitate oral tentacles white, but upper lamina of foot very faintly purple; no spots ventrally.

Specimen 2 (76 mm) was deeper orange in life, with more brown centrally and more yellow-orange marginally than specimen 1; faint submarginal band of yellow-orange before white margin. 13 large brown-red spots encircled with white, few smaller ones arranged irregularly around central patch. Additionally, this specimen had some white patches outside and amongst the small spots. 22 gills all had purple tips; rhinophores had more extensive purple pigment than those of specimen 1. Foot yellow-orange posteriorly followed by white band around margin. Ventrally foot bilaminate for its entire anterior margin (Fig. 10A). This specimen was dissected to remove the radula: body wall of foot thick and rose-red internally, digestive gland dark brown-pink while buccal mass white with pink tint. Muscles were very strong, glistening pink; reproductive system opaque white. The radula is large, but the teeth are minute: the largest laterals measure 100 μm (Fig. 10C). The formula is 58 (+2) × 56.1.56. There is a much reduced median thickening in some of the older rows (Fig. 10D). The first lateral bears a small rounded denticle on each side of a sharp cusp. The next 12 laterals have 10-12 weak denticles along the cusp (Fig. 10D). The remaining teeth are simply hook-shaped, blunt and rounded at the old end of the radula (Fig. 10E) but with extremely long sharp cusps at the newer end. Most of the jaw elements are simple unicuspid structures, with a single slightly curved cusp; very few are bicuspid.

Specimen 3 (75 mm) was dark like specimen 2, with 19 gills tipped in purple; rhinophores dark violet, with many closely spaced lamellae (Plate 37). Orange raised rhinophoral pocket rims can be seen in the plate. Ventrally, top of foot white with dark spots, violet margin on upper lamina of foot, white oral tentacles; orange-yellow marking posteriorly but no purple margin; faint orange line in crease between hyponotum and foot. Conical oral tentacles of preserved specimen visible in Fig. 10B, although left one is difficult to see. Margin of anterior foot distinctive, with the two laminae quite separated and extending across entire margin.

#### Remarks.

Many photographs of additional individuals from La Réunion as well as those from Sri Lanka (Plate 38) clearly belong to the same species. Variations range from large to small spots with solid to diffuse pigmentation, wine-red to almost black-red in colour, all with white ocelli; the presence or absence of smaller spots with a very broad to ‘normal’ white marginal band; a violet margin is very rarely present in the La Réunion individuals but present on the Sri Lanka individuals. Ventrally, there is also some variability, from pure white to having a (faint or dark) rusty orange line along the crease between the foot and the hyponotum, from no spots to few spots on the foot below this line, and there may or may not be an orange patch on the tail. The Sri Lanka individuals had a very faint purple line on the foot margin.

The specimens are all distinctive in preservative, either alcohol or formaldehyde: they are violet-purple to plum red-violet, and all have a thick undulating mantle skirt (including that from Chagos, see Yonow et al. 2002: 854). The gills spiral inwards at their ends and are triangular in section, with lamellae on two sides. The foot is broad anteriorly. No mantle glands are visible in any of the specimens.

Rudman (1987) initially synonymised Eliot’s species *Chromodoris cavae* with Kelaart’s *Chromodoris tennentana* (p. 44) but subsequently separated them into two species (http://www.seaslugforum.net/showall/chrotenn). Gosliner et al. (2008) retain this species as distinct from both *Chromodoris tennentana* and *Chromodoris leopardus*. Willan, based on 21 photographs on NudiPixel (http://www.nudipixel.net/species/chromodoris_tennentana/) decided that all variations belong to one species which is simply very variable from the western Indian Ocean to the West Pacific. However, the preserved specimens (including the one from Chagos, Yonow et al. 2002) are violet when preserved while all preserved *tennentana* (p. 44 and Yonow et al. 2002: 845) and *leopardus* (Yonow 2001: 23) are translucent. The gills in *tennentana* are flattened and simply pinnate (see p. 44) or sub-quadrangular (Rudman 1987: 364) while the gills of *leopardus* are triangular (Rudman 1987: 388); the gills of *cavae* are triangular. The gills and rhinophores of *tennentana* are coloured ochre with white tips, those of *leopardus* are tipped in violet (the gills rarely have an ochre rachis), and those of *cavae* are violet or tipped with violet. No mantle glands are visible in the preserved specimens of *cavae*, whereas they are obvious in *tennentana* (p. 44 and Fig. 11) and form a band around the mantle in *leopardus* (Rudman 1987).

Comparison of the radulae of *tennentana*, *vicina*, *leopardus*, cf. *leopardus*, and one La Réunion specimen show only slight variations in morphology but large differences in tooth size; the sizes listed are of the same dimension of a lateral tooth – from the tip of the cusp to the flange where the cusp meets the base.

 20 mm alive (Rudman, *tennentana*) 40(+2) × 37.0.37 40 µm

 36 mm alive (Edmunds, *vicina = tennentana*) 52 × 49.1.49 60 µm

 59 mm alive (Rudman, *leopardus*) 74(+2) × 61.0.61 90 µm

 34 mm alive (Yonow et al., cf. *leopardus = cavae*) 52 × 62.1.62 25 µm

 80 mm alive (this paper, *cavae*) 58(+2) × 56.1.56 100µm

The three specimens examined from La Réunion belong to a form of *Chromodoris cavae* in which the violet mantle margin is lacking; of the additional photographs of a further 25 individuals from La Réunion and Mauritius (P Bidgrain, pers. comm., H. Flodrops, pers. comm., and http://seaslugs.free.fr/nudibranche/a_intro.htm) all but one individual lack the violet margin on both the mantle and the foot. The Chagos specimens both had a pale purple margin to the mantle (Yonow et al. 2002) as did the individuals from Sri Lanka (Plate 38). *Chromodoris cavae* differs from both *Chromodoris tennentana* and *Chromodoris leopardus* in numerous features, all discussed above, and it is here removed from the synonymy of *Chromodoris tennentana*. *Chromodoris cavae* is recorded from Chagos, Sri Lanka, La Réunion, Zanzibar, and South Africa, indicating a western Indian Ocean distribution.

### Chromodoris conchyliata

Yonow, 1984

http://species-id.net/wiki/Chromodoris_conchyliata

Plates 39 40

Chromodoris cf. geometrica Risbec. – Rudman 1973a: 187, pl. 1B, figs. 7A, B (Kenya).
Chromodoris conchyliata Yonow, 1984: 216, fig. 2 (Sri Lanka); Debelius and Kuiter 2007: 148 (South Africa and Thailand).
Chromodoris geometrica Risbec. – Gosliner 1987: 76, fig. 111 (South Africa) (non *Chromodoris geometrica* Risbec).

#### Material.

Seychelles: 20 × 7 mm (PK-Z), 5 m depth under rock, off Ade’s house, Lilôt, NW Mahé, 10 April 1992, leg. P Kemp (specimen retains orange gills and rhinophores, examined 2009) and photos of one larger individual, P Kemp. – La Réunion and Mayotte: photographs of numerous individuals http://seaslugs.free.fr/nudibranche/a_intro.htm.

#### Remarks.

**Plate 39.**
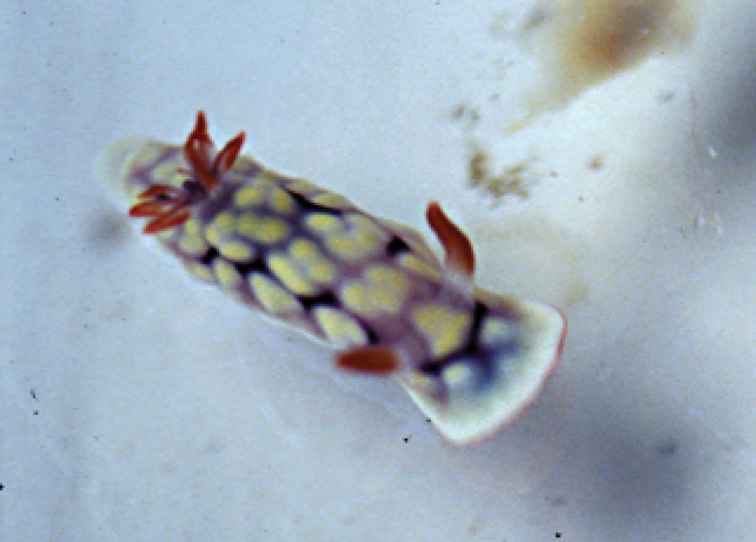
*Chromodoris conchyliata*, juvenile 20 mm, Seychelles, photo: P Kemp.

**Plate 40.**
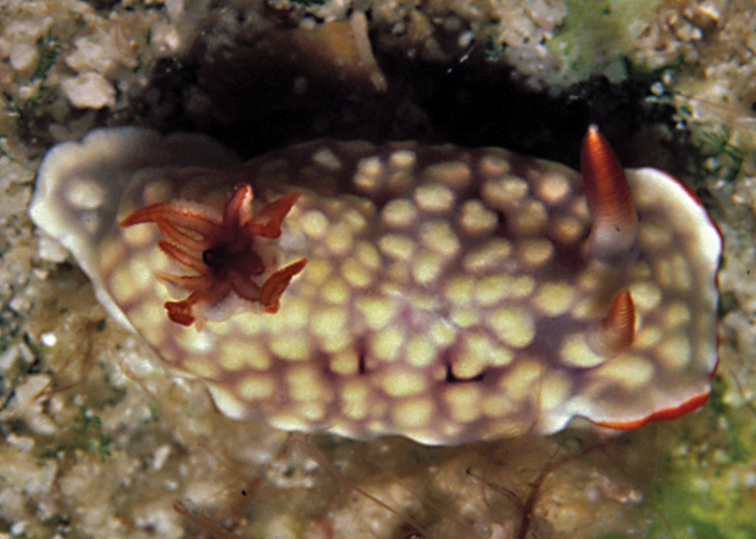
*Chromodoris conchyliata*, Seychelles, photo: P Kemp (no spcm).

It is exciting to be recording this species once more; there is one other published record from South Africa as *Chromodoris geometrica* (Gosliner 1987), and many photographs from La Réunion and Mayotte (http://seaslugs.free.fr/nudibranche/a_intro.htm), where it has been observed growing to 40 mm and spawning. This species displays ontogenetic changes: larger specimens are very irregularly pustulose, and the symmetrical violet markings around the large, regularly distributed, creamy yellow pustules of the juveniles (Plate 39) are sometimes reduced to just short dashes on each side in the large animals (Plate 40). In the larger specimens, the anterior ventral violet crescent of the smaller animals becomes a fiery orange-red (Plate 40). Most internet records are from the western Indian Ocean; a couple of photographs from west Thailand need confirmation (NudiPixel and Sea Slug Forum).

### Chromodoris decora

(Pease, 1860)

http://species-id.net/wiki/Chromodoris_decora

Plates 41 , 42

Doris decora Pease, 1860: 29 (Hawaii) and illustrated by Bergh 1880: 25, pl. B figs. 5-8.
Chromodoris decora . – Rudman 1986: 329, figs. 1, 9-11 (Christmas Island (Indian Ocean), Australia, New Caledonia); Debelius and Kuiter 2007: 149 (Oman, Sri Lanka + W Pacific).

#### Material.

Maldives: 10 mm (8 × 3 mm pres.), 12 m depth, Banana Reef, North Malé Atoll, 19 November 1995, leg. SG Buttress & RC Anderson (“dorsal surface creamy green with white dots, spots, and faint lines; margin orange with purple spots and white dots; larger purple spots inside margin; rhinophores and gills cream”). – Oman: photographs of one individual, Muscat, 01-12 April 2009, S Kahlbrock. – Sri Lanka: photographs of one individual, Unawatuna, S of Galle, 28 December 2010, S Kahlbrock.

#### Description.

**Plate 41.**
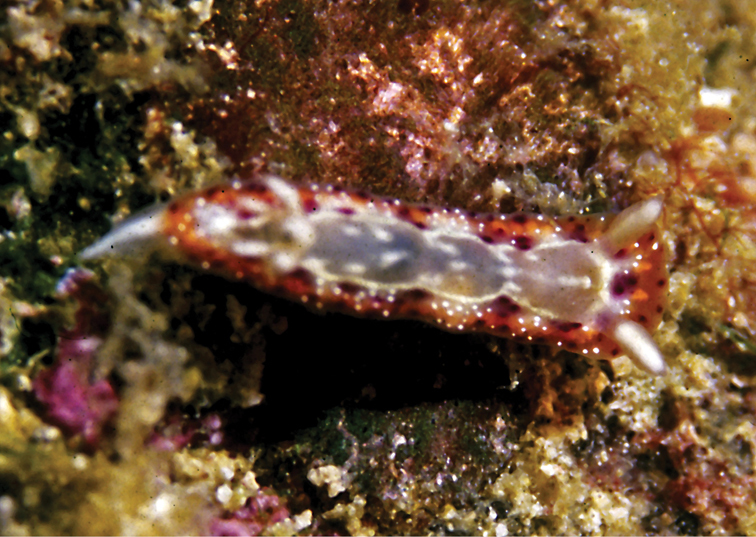
*Chromodoris decora*, 10 mm, Maldives, photo: RC Anderson.

**Plate 42.**
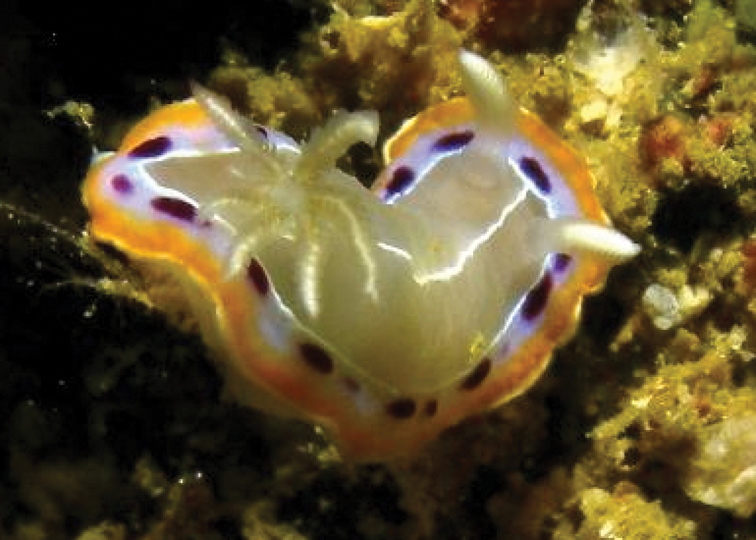
*Chromodoris decora*, Oman, photo: S Kahlbrock (no spcm).

The single specimen is accompanied by a photograph reproduced here (Plate 41) and is not of the typical colour pattern. The white lines were represented by a series of dots and dashes, forming an irregular scalloped line around the central dorsal hump, contiguous to an orange band containing larger purple spots and smaller white dots. Purple spots coalesced to form small patches. Some white dots and dashes within this central region, but only vaguely indicate a median. The five simply pinnate gills, rhinophores, and top of the tail all bore opaque white markings: the gills and the rhinophores as a core, and the tail as a white band centrally tapering on the long metapodium. Two individuals photographed in Oman and Sri Lanka have an identical pattern of distinct marginal bands of orange and lilac, the lilac bearing rounded purple spots (Plate 42). There is one white line dorsally splitting around the branchial pocket. One photograph shows the red-violet spots located between the foot and the hyponotum, characteristic of this species.

The preserved specimen is well extended but badly preserved. It is translucent white, and the black digestive gland is visible through the thin epidermis, as is the white intestine loop. The translucent white metapodium is long and pointed.

#### Distribution.

This is the first literature record of the species from the western Indian Ocean. There have been photographs made available on Sea Slug Forum and NudiPixel only from Sri Lanka, with all other records being further east from Christmas Island and the western Pacific.

### Chromodoris geometrica

Risbec, 1928

http://species-id.net/wiki/Chromodoris_geometrica

Plate 43

Chromodoris geometrica Risbec, 1928: 149, fig. 41, pl. 6 fig. 10 (New Caledonia); Yonow 1994a: 111, figs. 2H–L, 10C,12A, B (Maldives); Debelius and Kuiter 2007: 148 (Maldives + W Pacific).
non Chromodoris geometrica . – Gosliner 1987: 76, fig. 111 (South Africa) (= *Chromodoris conchyliata* Yonow).

#### Material.

Maldives: 15 mm, Banana Reef, North Malé Atoll, 12 m depth, 19 November 1995, leg. SG Buttress & RC Anderson, and photos of another individual, RCA. – Mayotte: photographs of numerous individuals http://seaslugs.free.fr/nudibranche/a_intro.htm.

#### Description/Remarks.

**Plate 43.**
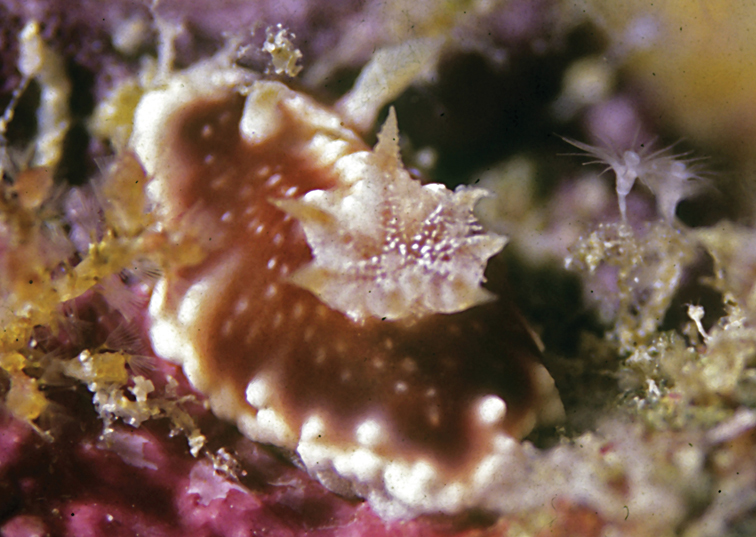
*Chromodoris geometrica*, 15 mm, Maldives, RC Anderson.

The small specimen illustrated in Plate 43 was very dark brown, with a few large pustules distributed centrally and marginally, and speckled greenish-yellow gills and rhinophores: Maldives specimens are usually darker brown than individuals from other localities. It is noteworthy that the only Indian Ocean specimens available are once again from the Maldive Islands. Currently, the only photographic records from anywhere else in the Indian Ocean are from Mayotte (http://seaslugs.free.fr/nudibranche/a_intro.htm), although there are other internet photos of this Indo-West Pacific species from Tanzania and South Africa (Sea Slug Forum) and the Maldives, Kenya, and Madagascar (NudiPixel).

### Chromodoris gleniei

(Kelaart, 1858)

http://species-id.net/wiki/Chromodoris_gleniei

Plate 44

Doris gleniei Kelaart, 1858: 95 (Sri Lanka).
Chromodoris gleniei . – Rudman 1987: 391, figs. 39, 47-49 (Tanzania and Maldives); Yonow 1994a: 115, fig. 10E (Maldives); Yonow et al. 2002: 854, fig. 11d (Chagos); Debelius and Kuiter 2007: 146 (Maldives, Kenya, South Africa, Andaman Sea).

#### Material.

Maldives: 25 mm, Fulidhoo Channel, Felidhoo Atoll, 10 m depth, 24 April 1994, leg. RC Anderson & SG Buttress; photographs of one individual 40 mm, March 1999, J Hinterkircher. – Sri Lanka: photographs of mating pair, Negombo, N of Colombo, 16 January 2011, S Kahlbrock.

### Chromodoris hamiltoni

Rudman, 1977

http://species-id.net/wiki/Chromodoris_hamiltoni

Plate 45

Chromodoris hamiltoni Rudman, 1977: 374, pl. 1A, num figs. (Kenya, Tanzania); Gosliner 1987: 74, fig. 104 (South Africa); Debelius and Kuiter 2007: 172 (Mauritius, Mozambique, South Africa); Richmond 2011: 270 (East Africa).

#### Material.

Kenya: six specimens 15-20 mm pres. lengths, Vipingo, 25 miles N of Mombasa, ELW in rock pools on exposed reef, 23 September 1984, leg. J Hognerud (Australian Museum, Sydney, C431127). – Tanzania: photographs of two individuals, 1994, and reef flat, Matemwe, August 1993, MD Richmond; photo of one individual, Mafia Island, shallow water, May 2009, A de Villiers. – La Réunion: photograph of single individual, P Bidgrain, pers. comm.

#### Description/Distribution.

The preserved specimens were blue in life with a broad pale orange mantle skirt and a distinctive orange patch in the centre of the dorsum, bisected by a longitudinal black line. All published records are listed above, and indicate that this species is known only from eastern Africa: *Chromodoris hamiltoni* has not been recorded from any of the island groups except La Réunion. It is similar only to *Chromodoris quadricolor* (see below), but usually lacks the distinctive white line between the black outermost line and the orange margin, the orange margin is paler and slightly more pink, and usually it has additional patches of orange and/or black dorsally. It is a much more variable species than *Chromodoris quadricolor*.

### Chromodoris quadricolor

(Rüppell & Leuckart, 1830)

http://species-id.net/wiki/Chromodoris_quadricolor

**Figure 12.**
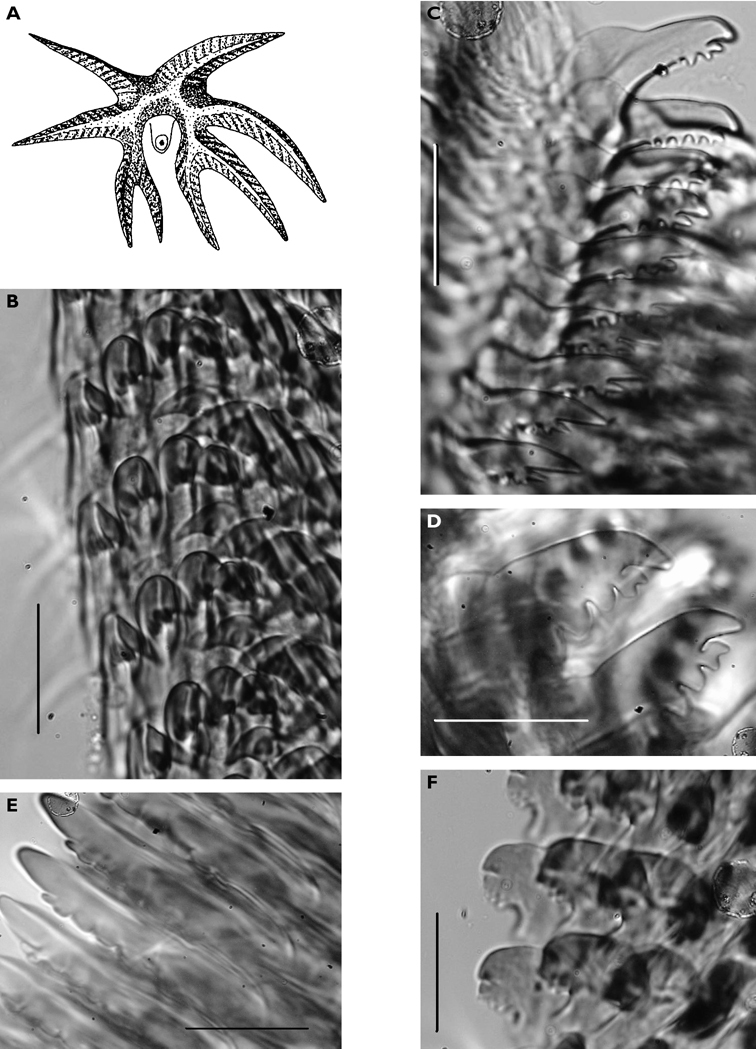
*Risbecia bullockii* **A** view of gills showing their arrangement (compare with Plate 35 “fig. 17”). Radular teeth of 16 mm pres. Sri Lankan specimen **B** first lateral teeth from right half of radula **C** view of one row of lateral teeth showing divided first denticle on each cusp **D** higher magnification of two lateral teeth **E** several laterals from the newest row, also with divided first denticles **F** outermost laterals showing very reduced cusps but still with divided first denticles. Scale bars 50 µm.

Doris quadricolor Rüppell & Leuckart, 1830: 31, pl. 9 (Red Sea).
Chromodoris quadricolor . – Rudman 1977: 370, pl. 1B, figs. 19, 20 (Tanzania); Yonow 1989: 296, pl. 8 (Red Sea); Yonow et al. 2002: 852 (part), fig. 11f only (Chagos); Debelius and Kuiter 2007: 166 (Red Sea); Yonow 2008: 176 (Red Sea).
non Chromodoris quadricolor . – Yonow et al. 2002: 852 (part), figs. 11e, 12A, B (Chagos) [= *Chromodoris elisabethina* Bergh].

#### Material.

Socotra: 20 × 9 mm pres. (St-190, F-59), Abd al-Kuri, Khaisat en Naum, western tip, 10 April 1999, leg. U Zajonz.

#### Description.

There are no photographs accompanying the Socotra specimen, but the preserved specimen is semi-relaxed (examined 2009), with dark orange rhinophores and gills. The mantle is black with two white stripes fading to cream with a faint orange tinge on the dorsum. There is a relatively broad white band between the black dorsum and the orange marginal band (cf. *Chromodoris hamiltoni* above, which normally does not have this white separation, and *Chromodoris elisabethina* Bergh, which has a fine white line). The white line at the very edge of the margin can still be seen (cf. *Chromodoris africana* p. 34 which does not have this white line). There are two black stripes on the sides of the foot, and the foot retains an orange margin. The preserved specimen is identical to specimens collected from the Red Sea in 1990: *Chromodoris quadricolor* appears to hold its colour extremely well in formaldehyde.

#### Remarks/Distribution.

Originally described from the Red Sea, its presence in Socotra is not unexpected: Rudman (1977) had specimens from Tanzania and Yonow et al. (2002) subsequently reported specimens from Chagos. Yonow et al (2002) discussed the similarities with *Chromodoris elisabethina*: five specimens from that collection were correctly attributed to *Chromodoris quadricolor* (Chag96/42, 96/51, 96/52a, 96/52b, 96/52c, and 96/87, colour figure 11f); however, two specimens are here reassigned to *Chromodoris elisabethina* (Chag96/16 and 96/87, colour figure 11e, and radular preparation, fig. 12a, b). Re-examination of the specimens and additional colour slides revealed that these animals both had darker patches located centrally as well as behind the rhinophores, clearly visible in the figure of specimen Chag96/87e in Yonow et al. 2002 (fig. 11e) and typical of *Chromodoris elisabethina*. The yellow margin differs in colour from that of the more orange rhinophores and gills, another characteristic of *Chromodoris elisabethina* and visible in the illustrations. *Chromodoris elisabethina* is more likely to have more black lines than *quadricolor*: *Chromodoris quadricolor* normally only has three black lines on the dorsum, and the Red Sea specimens with more black lines have been identified as *Chromodoris strigata* Rudman (Yonow 2008). *Chromodoris quadricolor* is restricted to the Red Sea, where it is very common, apart from isolated specimen records from Socotra, Tanzania, and Chagos.

### Chromodoris tennentana

(Kelaart, 1859)

http://species-id.net/wiki/Chromodoris_tennentana

Fig. 11 Plate 46

Doris tennentana Kelaart, 1858: 268 (Sri Lanka).
Chromodoris vicina Eliot, 1904a: 392 (Zanzibar); Edmunds 1971: 372, fig. 16, pl. 1A (Tanzania).
non Chromodoris vicina . – Gosliner 1987: 75, fig. 109 (South Africa) (= *Chromodoris cavae* Eliot).
Chromodoris tennentana . – Rudman, 1987: 364, figs. 23, 32, 35, 36 (Tanzania); Yonow et al. 2002: 855, fig. 13a (Chagos); Debelius and Kuiter 2007: 151 (South Africa); Richmond 2011: 280 (East Africa).

#### Material.

Seychelles: 8 × 5 mm pres. (PK–BB), Lilôt, NW Mahé, 17 m depth on algal-encrusted rock, 26 April 1992, leg. P Kemp (mantle glands obvious on preserved specimen). – Madagascar: photos of one juvenile, 10 mm in length, Nosy Bé, October 2007, J Hinterkircher.

#### Description.

The Seychelles specimen has the typical colour pattern: a central patchy ochre-brown dorsum with ocellated violet spots, a creamy sub-marginal band with orange and purple spots, a blue-violet margin, and ochre rhinophores and gills. The specimen preserved in formaldehyde is translucent, with a rosy-orange and black gut. The flat translucent mantle skirt is extended, white mantle glands visible in a band along the edge: most are irregular in shape, some are stellate (Fig. 11A). Gills translucent, flat, and very simply pinnate; oral tentacles digitiform (Fig. 11B). The similarities and differences between this species and *Chromodoris cavae*, removed from synonymy of *Chromodoris tennentana*, are discussed under that species (see p. 36).

### Glossodoris cincta

(Bergh, 1888)

http://species-id.net/wiki/Glossodoris_cincta

Plate 47

Glossodoris cincta . – Yonow 1994a: 120, fig. 10H (Maldives); Yonow et al. 2002: 856, fig. 13c (Chagos); Debelius and Kuiter 2007: 195 (Red Sea, Mozambique + W Pacific); Apte 2009: 170, fig. 2h (Laccadive Islands); Richmond 2011: 280 (East Africa); Yogesh Kumar et al. 2011: 109, fig. 2a (India).

#### Material.

Seychelles: 30 × 15 mm (PK-N), Horseshoe Rock, Lilôt, NW Mahé, 15 m depth on algal-encrusted rock, 24 March 1992, leg. P Kemp (“pink with dark blue edging”; the preserved specimen is brick red with a green margin). – Maldives: 15 mm (7 mm pres.), 5 m depth on coral encrusted with algae and sponge, Maayafushi, South Malé Atoll, March 1997, leg. J Hinterkircher. – Persian Gulf: photograph of one individual, Dahwat ad Daffi, Jubail, Saudi Arabia, April 1992, F Krupp. – Kenya: 30 mm long pres., Vipingo, 25 m N of Mombasa, ELW in rock pools on exposed reef, 23 September 1984, leg. J Hognerud (preserved animal more orange and purple than red and blue-green) (Australian Museum, Sydney, lost?). – Tanzania: photographs of two individuals, Mafia Island, shallow water, May 2009, A de Villiers. – Sri Lanka: photographs of one individual, Unawatuna, south of Galle, 01 January 2011, S Kahlbrock.

### Glossodoris pallida

(Rüppell & Leuckart, 1830)

http://species-id.net/wiki/Glossodoris_pallida

Plate 48

Doris pallida Rüppell & Leuckart, 1830: 33, tab. 10, fig. 1 (Red Sea).
Glossodoris pallida . – Rudman 1984: 145, figs. 1, 18-20, 27 (Red Sea, Tanzania, Great Barrier Reef); Yonow 1989: 300 (Red Sea); Yonow 2000: 131 (Red Sea); Debelius and Kuiter 2007: 185 (Oman, Mozambique, Myanmar, Viet Nam); Yonow 2008: 191 (Red Sea).

#### Material. 

Maldives: approx. 20 mm alive (11 × 5 mm pres.), Bathala Island, Ari Atoll, 11 m depth, night dive, 27 July 1995, leg. SG Buttress & RC Anderson. – Persian Gulf: photographs of two individuals, Dahwat ad Daffi, April 1992, and N of Abu Ali, 26 May 1992, Jubail, Saudi Arabia, F Krupp; photo of three individuals, Muscat, Oman, 01-12 April 2009, S Kahlbrock. – La Réunion: photos of several individuals 6-20 mm http://seaslugs.free.fr/nudibranche/a_intro.htm. – Seychelles: photographs of one individual, Lilôt, NW Mahé, 1988-1989, P Kemp. – Sri Lanka: photographs of two individuals, Unawatuna, S of Galle, 03 January 2011, S Kahlbrock.

#### Description.

The preserved specimen is well relaxed: it is opaque white, and no markings remain; the digestive system is not visible through the skin. Twelve gills are extended: the rachis is flat on the outside and lamellae face inwards on the two other sides. In life, the lamellae can be seen to extend laterally. One photograph from Oman (S Kahlbrock, pers. comm.) depicts the individual beginning to lay an egg mass, a flat white ribbon laid on its edge.

#### Distribution.

**Plate 48.**
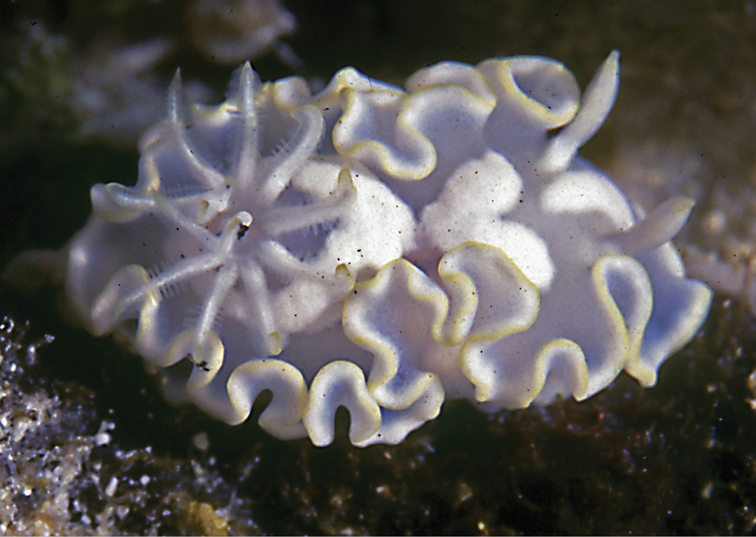
*Glossodoris pallida*, 20 mm, Maldives, photo: RC Anderson.

This species appears to be present but rarely recorded in the western Indian Ocean: Rudman (1984) had one only specimen from Tanzania; the specimen above is a first record for the Maldives (Plate 48), and new photographic records are presented here from the Persian Gulf, Gulf of Oman, Seychelles, La Réunion (http://seaslugs.free.fr/nudibranche/a_intro.htm), and Sri Lanka.

### Glossodoris undaurum

Rudman, 1985

http://species-id.net/wiki/Glossodoris_undaurum

Plate 49

Glossodoris undaurum Rudman, 1985: 248, figs. 1C, 3B, 5 (SW Australia).
Glossodoris undarum . – Debelius & Kuiter 2007: 189 (South Africa and W Australia); Gosliner et al. 2008: 239 (South Africa, Oman, W Australia) (all misspellings).

#### Material.

Sri Lanka: 13 × 5 mm pres., Unawatuna, S of Galle, 27 December 2010, leg. and photos S Kahlbrock.

#### Description.

This description of the live animal is based on many photographs of the specimen: dorsum broad with narrow mantle overlap; skirt thick with single pair of permanent undulations. Dorsum grey to off-white with irregularly shaped white, slightly raised pustules. Mantle skirt whiter than dorsum with only a few small white pustules; margin lemon-yellow. Rhinophores very small: distal half translucent lemon-yellow, proximal half paler translucent yellow, posteriorly an opaque line brighter yellow towards the tip. Simply pinnate gills translucent white with opaque white line on each side of rachis, red tips, and short red line running down from tips.

The preserved specimen is semi-translucent grey with irregular opaque white spots, slight crumpling of mantle around each. Mantle skirt with thickened margin, no coloured or thickened margin on skirt or foot. Long rhinophores translucent grey with white line and white tip, lamellae indistinct. Gills retracted but the red ends visible though translucent skin and open pocket.

#### Distribution.

This is the first record in the scientific literature of *Glossodoris undaurum* from the western Indian Ocean. There are book and internet records from South Africa, Kenya, Oman, and La Réunion. There are several named and un-named species occurring in the Indian Ocean, but these differ externally in the combination of margin, rhinophore, and gill colouration: collection of specimens will enable comparisons. It may be that some are colour variations but this cannot be established at present.

### Hypselodoris maculosa

(Pease, 1871)

http://species-id.net/wiki/Hypselodoris_maculosa

Plate 50

Hypselodoris maculosa . – Rudman 1986: 340, figs. 1, 13, 16-18 (Red Sea, Tanzania, Christmas Island + W Pacific); Yonow 1989: 302, pl. 21 (Red Sea); Yonow et al. 2002: 858, fig. 13e (Chagos); Apte 2009: 170, fig. 2i (Laccadive Islands).

#### Material.

Seychelles: 15 × 5 mm (PK-DD), Lilôt, NW Mahé, 17 m depth on granite wall, 26 April 1992, leg. P Kemp. – Maldives: 15 mm length, Maayafushi, Ari Atoll, 3m depth, on coral rubble encrusted with algae and sponges, March 1997, leg. J Hinterkircher. – La Réunion, Mauritius, and Mayotte: numerous photographs http://seaslugs.free.fr/nudibranche/a_intro.htm. – Sri Lanka: photographs of one individual, Unawatuna, S of Galle, 27 December 2010, S Kahlbrock.

#### Description.

Elongated body with spatulate head. Translucent body with opaque white markings comprising several longitudinal white lines on dorsum and small white dots on orange-red margin. Magenta spots and patches between dorsal white lines. Foot translucent with magenta spots and patches, and small white dots. Rhinophores distinctive with white core bearing two red bands. Gills small, held upright, red proximally and white distally.

#### Remarks.

Small specimens are similar to several species of *Thorunna*, such as *Thorunna australis* (Risbec), recorded only from South Africa in the western Indian Ocean: it also has orange bands on the gills and rhinophores but the white markings are limited to one or two lines on the dorsum. *Thorunna florens* (Baba) also has similar gills and rhinophores but has purple spots around the margin as well as an orange border to the frontal margin, and it is limited to the western Pacific. Several un-named species are illustrated in Gosliner et al. (2008) but at present they are only known from few localities in the western Pacific.

### Risbecia bullockii

(Collingwood, 1881)

http://species-id.net/wiki/Risbecia_bullockii

**Plate 51.**
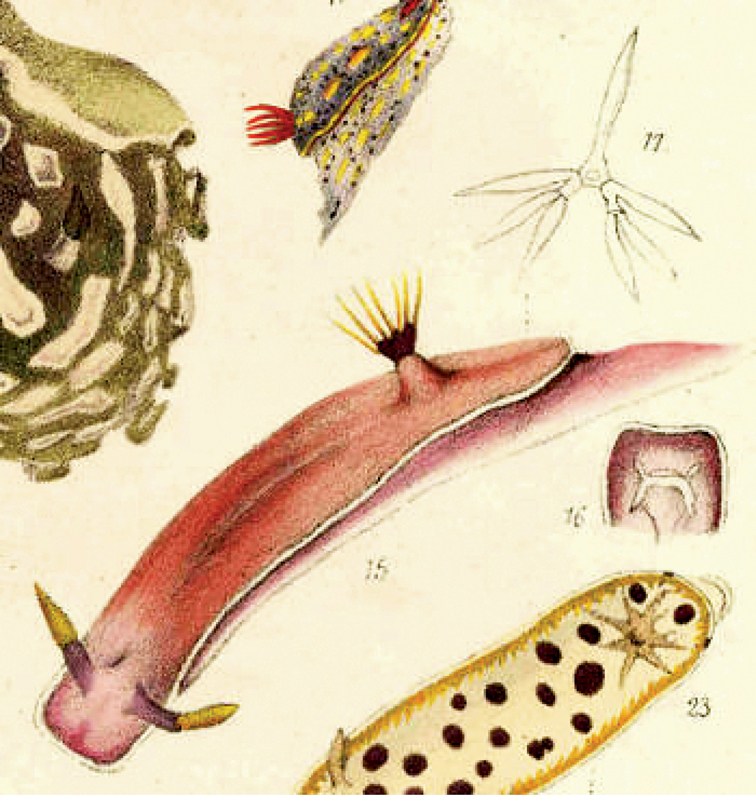
*Chromodoris bullockii* Collingwood, 1881: figs 15, 16, 17.

Fig. 12 Plates 51 52 53

Chromodoris bullockii Collingwood, 1881: 128, pl. 9, figs. 15-17 (South China Sea); Yonow 2001: 9, pl. 1, fig. 6 (Indonesia).
Hypselodoris bullockii . – Marshall & Willan 1999: 106, figs. 191, 19 (Great Barrier Reef); Debelius and Kuiter 2007: 116 (South Africa + W Pacific); Yogesh Kumar et al. 2011: 109, fig. 2b (India).

#### Material.

La Réunion: 30 mm (24 × 10 mm pres.) on wall of cave, Etang Salé les Bains, 38 m depth, 21 December 2010, leg. H Flodrops; 35 mm (23 × 10 mm pres.), Sec Jaune, Saint-Leu, 12 m depth (night dive), 30 December 2010, leg. H Flodrops. – Mauritius: photographs only of three individuals, 1990’s, M. Parmantier pers. comm. and http://seaslugs.free.fr/nudibranche/a_intro.htm. – Sri Lanka: three specimens 11 × 5 mm, 13 × 4 mm, and 16 × 7 mm all pres., Unawatuna, S of Galle, 29 December 2010, and Negombo, N of Colombo, 16 January 2011, leg. S Kahlbrock; photo of one individual, Pigeon Island, Trincomalee, 16 m depth, April 1995, RC Anderson.

#### Description.

**Plate 52.**
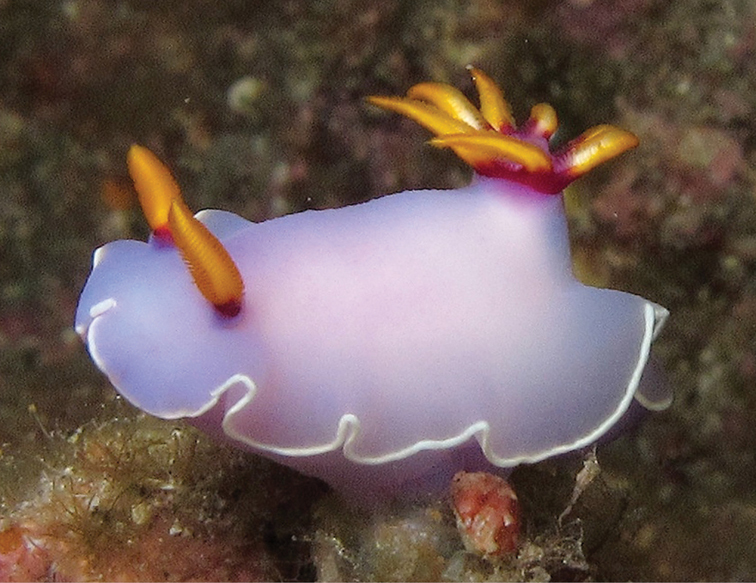
*Risbecia bullockii*, 35 mm, La Réunion, H Flodrops.

**Plate 53.**
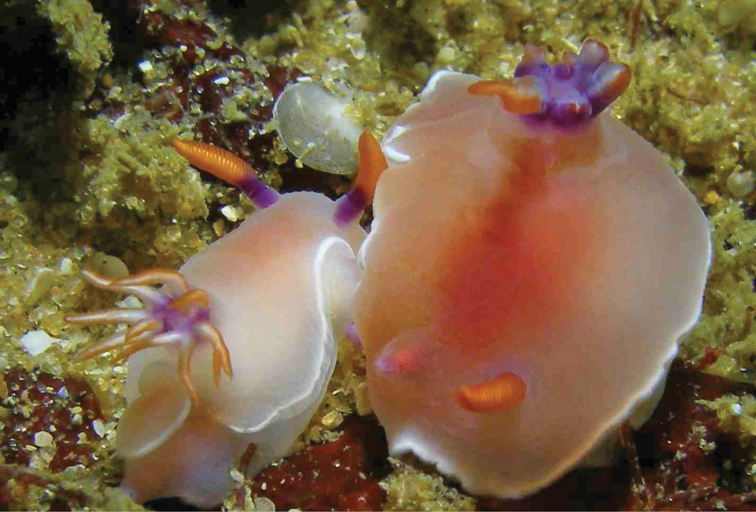
*Risbecia bullockii*, 11 and 16 mm pres., Sri Lanka, S Kahlbrock.

All five specimens are of the original colour form as described and illustrated by Collingwood (1881) and reproduced on Plate 51: dorsum mauve with central orange tinge and distinct narrow white margin; gills and rhinophores bright orange with magenta bases, gills raised high on a peduncle. La Réunion specimens pale violet with white margin, bicoloured gills and rhinophores (Plate 52). Smaller Sri Lanka specimens with rosier orange central dorsum (Plate 53). Seven gills in La Réunion specimens and nine in Sri Lanka specimens. In some photographs, the peculiar arrangement described and illustrated by Collingwood (Plate 51, "fig. 17") is visible, in which the gills are connected: the last two or three gills on each side are joined at the base and there are three single central ones (Fig. 12A). Anal papilla protruded as a tube in most images showing open gills, same colour as mantle. Gills bright orange but deep magenta where connected basally. Pockets of both rhinophores and gills uniformly coloured with mantle; raised rim translucent. White marginal line present on both surfaces as well as edge of mantle.

The specimens are well preserved, solid with a high profile, the metapodium extending beyond the posterior mantle margin. All five specimens retain the white line around the margin but no mantle glands are evident. The gills are extended in the La Réunion specimens, and each gill is almost round in section. Those of the 35 mm specimen are still pink, especially at the base (April 2011). The head is rounded with small oral tentacles identical to that figured for *Risbecia apolegma* Yonow (2002, fig. 11). The rosy hue remains on the dorsum, rhinophores, and gills of the Sri Lanka material several months after collection. The radula of the 16 mm Sri Lankan specimen examined had a formula of 72 × 100.0.100. All teeth are denticulate: there is no median thickening; the first 10 laterals in each row are recurved: the main cusp has a strong “brow ridge” with 1 or 2 very small denticles on each side (Fig. 12B). The majority of the laterals rapidly become straighter with a large cusp bearing 5 or 6 small denticles along its edge; the largest cusp measures at least 100 μm. In the last 5-7 teeth of this group, the first denticle next to the main cusp is doubled (Fig. 12C, D), even in the newest row (Fig. 12E). The final 10 teeth of each row rapidly reduce in size of cusp and denticles to become almost rounded; however, the double first denticle persists (Fig. 12F). The jaw rodlets of *Risbecia bullockii* have a slightly curved point with a swollen base.

#### Remarks.

Much controversy surrounds the identity of this species, here recorded from the Indian Ocean for the second time: photographs from La Réunion and Mauritius are also amethystine violet, intensifying anteriorly and posteriorly, a thin, bright white line is present around the mantle but not around the foot, and the gills and rhinophores are orange with magenta at the base (http://seaslugs.free.fr/nudibranche/a_intro.htm); these are identical to Collingwood’s illustration. Controversy also surrounded the description of *Risbecia apolegma* Yonow, 2002: Rudman stated on Sea Slug Forum that he could “find no anatomical grounds to place this ‘species’ in the genus *Risbecia*” while also querying its distinction from *Hypselodoris* “*bullocki*” (sic). However, in the numerous pages showing variations in colour pattern he stated that “unpublished studies of the anatomy show that it should probably be placed in the genus *Risbecia*.” Examination of the radula of the La Réunion specimen, re-examination of all micrographs of the radulae of *Risbecia apolegma* and of *Risbecia pulchella* (see below) show that they are all very similar and bear the diagnostic features as described by Rudman (1984). None of these teeth has the bicuspid laterals typical of the genus *Hypselodoris*; another character they have in common is that there are fewer rows than teeth per half row, in contrast to a statement by Gosliner and Johnson (1999) to the opposite effect. The teeth of *Risbecia apolegma* have a single large cusp with 5-6 denticles, the first of which faintly shows the division that is so obvious in *bullockii* and *pulchella*. Whether one assigns *apolegma* to *Risbecia* or *Hypselodoris*, it is clearly different from *bullockii* and a valid species based on radular morphology and consistent colour pattern.

Coincidentally, a recent paper by Johnson (2010) presented a phylogenetic tree placing *Hypselodoris bullockii* with *Risbecia imperialis* and *Risbecia tryoni*. Several species of *Hypselodoris* have a high body profile and an elevated gill peduncle, of which *Hypselodoris regina* Ev. Marcus & Er. Marcus is found along the East African coastline: the gills in this species nearly always form a goblet shape unlike those of *Risbecia bullockii*, which are similar to those of *Risbecia apolegma*.

### Risbecia pulchella

(Rüppell & Leuckart, 1830)

http://species-id.net/wiki/Risbecia_pulchella

Fig. 13 Plate 54

Doris pulchella Rüppell & Leuckart, 1830: 32, tab. 9, fig. 5 (Red Sea).
Risbecia pulchella . – Rudman, 1984: 203, fig. 65, 67, 68 (Kenya, Tanzania, and Mayotte, Comoro Islands); Gosliner 1987: 83, fig. 132 (South Africa); Yonow 1989: 303, pl. 23-25 (Red Sea); Debelius and Kuiter 2007: 138 (Red Sea, Mozambique, and South Africa); Yonow 2008: 201 (Red Sea); Yogesh Kumar et al. 2011: 109, fig. 2e (India).

#### Material.

Seychelles: 50 × 25 mm, one of two animals on algal-encrusted rock, Bug Rock, Brissare, 05 April 1992, leg. P Kemp; photographs of several individuals, Lilôt, NW Mahé, 1988-1989, P Kemp. – Gulf of Oman: photos of two individuals trailing, Muscat, 01-12 April 2009, S Kahlbrock; photographs of pairs, Musandam, 13 May 2009, trailing pair, Siri Island, 15 m depth, 13 May 2009, trailing pair, Anemone Garden, just off Khorfakkan, 8 Jan 2012, GT Smith. – La Réunion, Mauritius, Mayotte: photographs of several individuals http://seaslugs.free.fr/nudibranche/a_intro.htm.

#### Description.

**Figure 13.**
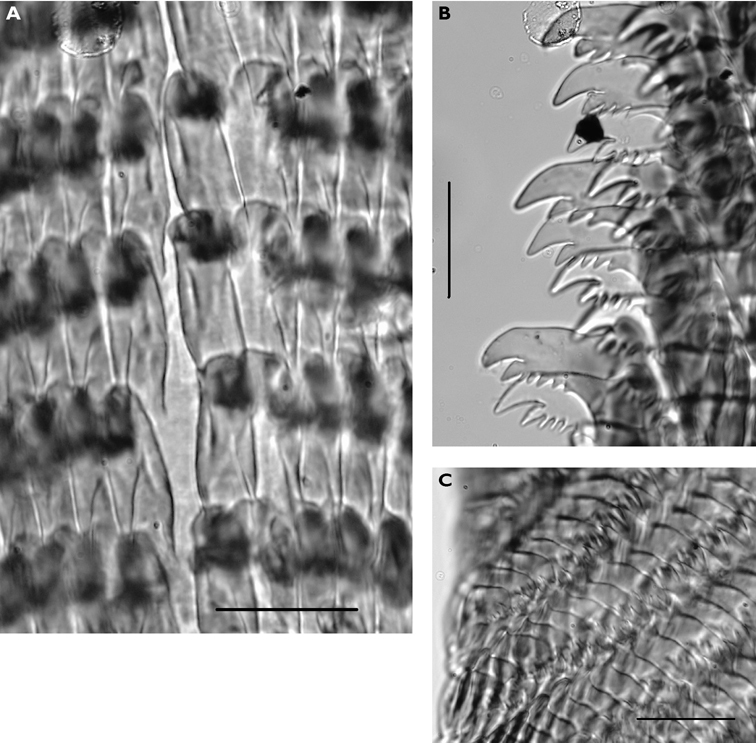
*Risbecia pulchella*, 50 mm **A** first 3–5 laterals on both sides of the midline, no central thickening or rhachidian **B** mid-lateral teeth showing single main cusp and divided first denticle **C** outermost laterals in newish row with reduction in cusp. Scale bars 50 µm.

The radular formula is 83 (+3) × 90-95.0.95-90: there is no median tooth. The first 7-8 laterals are duck bill shaped with a large denticle on the outside (Fig. 13A). The central laterals increase rapidly in size to a cusp length of approximately 75 μm bearing 4-5 denticles, of which the first is divided (Fig. 13B). The last 3-4 laterals in each row are very reduced with a rounded main cusp and multiple denticles (Fig. 13C).

### Thorunna horologia

Rudman, 1984 cf.

http://species-id.net/wiki/Thorunna_cf_horologia

Fig. 14 Plate 55

Thorunna horologia . – Debelius & Kuiter 2007: 199, right image only (South Africa).
Thorunna sp 3. – Gosliner et al. 2008: 253 (Indonesia).

#### Material.

Sri Lanka: 5 × 4 mm pres., Pigeon Island, Trincomalee, 16 m depth, April 1995, leg. SG Buttress & RC Anderson.

#### Description.

The single specimen appears to have been stocky with little mantle overlap in life. Margin permanently convoluted with three indentations: one behind rhinophores, largest in centre of body, one just beyond gills. Colour opaque white with violet pigment concentrated around edges and on head around and between rhinophores. Thin sub-marginal white line around the violet, followed by translucent margin with orange in patches and in permanent folds, most especially the central one in which there was orange pigment overlying the submarginal white line. Rhinophores very large with translucent stalk and white core covered in 13 orange-red lamellae; rhinophoral pockets with low violet rim. Eight gills simply pinnate, arranged in circle around anal papilla; white ‘core’ and orange lamellae; orange line along both inner and outer edges of each gill. Foot pale violet, darker concentration of violet in band around metapodium.

**Figure 14.**
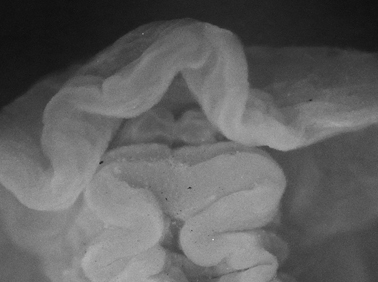
*Thorunna cf. horologia*, 5 mm pres. specimen, ventral view of propodium showing bilaminate edge and oral tentacles.

The preserved specimen is translucent cream, with a thin mantle skirt. The rhinophores are retracted but some of the gills are extended: they are simply pinnate. The foot is convoluted laterally but anteriorly it is relaxed. The bilaminate margin spans the entire width and the upper lamina is notched. The oral tentacles are plump rounded structures (Fig. 14).

#### Remarks.

This specimen differs from *Thorunna horologia*, also found in the western Indian Ocean: *horologia* is opaque white with red marginal and orange-yellow sub-marginal lines, a single deep indentation which is red, and a white foot with a violet band on the margin (Rudman 1984, Gosliner et al. 2008).

### Dendrodoris fumata

(Rüppell & Leuckart, 1830)

http://species-id.net/wiki/Dendrodoris_fumata

Plate 56

Doris fumata Rüppell & Leuckart, 1830: 29, pl. 8, fig. 2 (Red Sea).
Dendrodoris rubra (Kelaart). – Edmunds 1971: 386, fig. 22 and references therein (Tanzania).
Dendrodoris sp. – Yonow & Hayward 1991:26, figs. 8G-I (Mauritius).
Dendrodoris fumata . – Brodie et al. 1997: 413 and references therein except black form (= *arborescens* Collingwood) (Tanzania); Yonow 2008: 211 (Red Sea); Apte et al. 2010: 18, fig. 2g (India).

#### Material.

Socotra: 17 × 10 mm pres. (IT-177, RJ-032), 12°41.326'N, 54°05.175'E, 14 April 1999, leg. R Janssen. – Persian Gulf: four pres. specimens 17 × 10 mm, 15 × 12 mm, 16 × 10 mm, and 9 × 5 mm, Rescue Centre, Jubail, Saudi Arabia, 09 December 1991, leg. D Fiege; photographs of one individual, Dahwat al Musallamiya, Jubail, Saudi Arabia, 25 November 1991, F Krupp. – La Réunion: photographs of four individuals http://seaslugs.free.fr/nudibranche/a_intro.htm.

#### Description.

**Plate 56.**
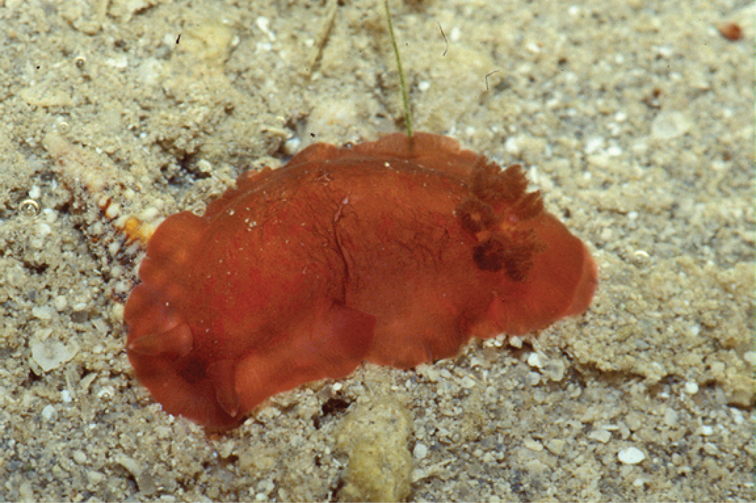
*Dendrodoris fumata*, Saudi Arabia, photo: F Krupp (no spcm).

Both *Dendrodoris nigra* and *Dendrodoris fumata* are found in similar habitats in the western Indian Ocean, and both species are present in these collections. Two colour forms occur in the Indo-West Pacific, a grey form and an orange/red form, the latter being much more common in the Indian Ocean (pers. obs., Brodie et al. 1997). As there were no colour photographs accompanying these specimens but photographs of uncollected individuals from the same region (Plate 56), identification is based on the morphology and colour in preservative, considerably different from that of *Dendrodoris nigra* (see also discussion for *Dendrodoris nigra* below) and other dendrodorids (see below). The *Dendrodoris fumata* specimens are contracted and semi-translucent; one is slightly lumpy but they are otherwise smooth and domed with a wide mantle skirt.

A third species of *Dendrodoris* is present in the Persian Gulf material: the preserved animals are very different from both *Dendrodoris fumata* and *Dendrodoris nigra*, but there are no notes or photographs of the living material for adequate comparison or identification (Abu Ali, 28 March 1992). In preservative, these two specimens measure 10 × 8 mm and 15 × 9 mm. They are both translucent beige-yellow and may have been pustular (? possibly *Dendrodoris coronata* Kay & Young). Both have very prominent raised rhinophoral and branchial sheaths; the smaller specimen has 9 extended simply pinnate gills and the rhinophoral pockets are crumpled in both specimens. Ventrally, the foot is broad and squared anteriorly with two laminae, and the ridge-like oral tentacles meet medially as in all dendrodorids.

### Dendrodoris nigra

Stimpson, 1855

http://species-id.net/wiki/Dendrodoris_nigra

Plates 57 , 58

Dendrodoris nigra . – Edmunds 1971: 383, fig. 21 and references therein (Tanzania); Gosliner 1987: 87, fig. 144 (South Africa); Debelius and Kuiter 2007: 259 (Oman, Persian Gulf + W Pacific); Yonow 2008: 212 (Red Sea); Apte 2009: 173, fig. 3e (Laccadive Islands); Richmond 2011: 282 (East Africa).

#### Material.

Socotra: two pres. specimens 18 × 13 mm and 16 × 9 mm (IT-177, RJ-032; the smaller has a white submarginal band), 12°41.326'N, 54°05.175'E, 14 April 1999, leg. R Janssen. – Maldives: two specimens 16 × 4 mm and 19 × 5 mm (MDV/AB/96/6), found separately under rocks, Fulidhoo Lagoon, Felidhoo Atoll, 04 May 1996, leg. RC Anderson & SG Buttress; photo of one red individual, 1986-1994, J Hinterkircher. – Zanzibar: 30 × 15 mm pres. (“black, frilly skirt, domed dorsum, no white specks”), in coral rock, 1994, leg. MD Richmond. – Gulf of Oman: photo of three individuals, Muscat, 01-12 April 2009, S Kahlbrock. – Seychelles, one individual photographed, Lilôt, NW Mahé, 1988-1989, P Kemp. – La Réunion, Mauritius, and Mayotte: photographs of several individuals http://seaslugs.free.fr/nudibranche/a_intro.htm.

#### Description.

**Plate 58.**
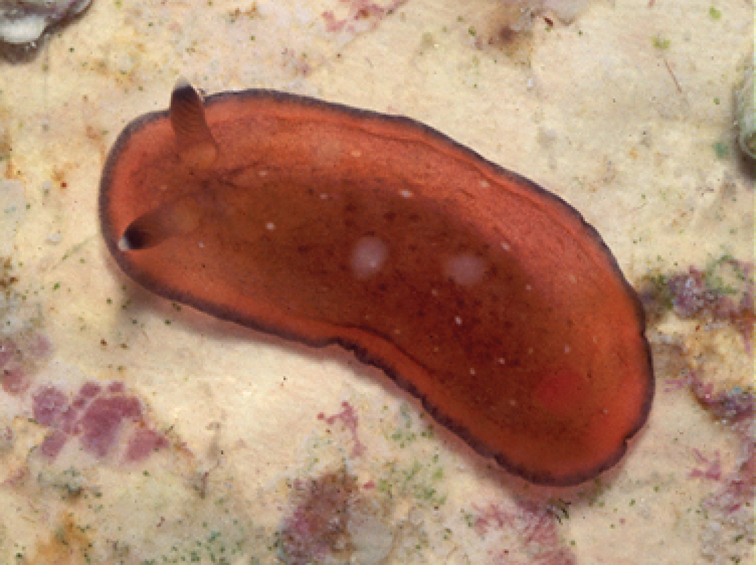
*Dendrodoris nigra*, red juvenile, Maldives, photo: J Hinterkircher (no spcm).

The preserved specimens of *Dendrodoris nigra* are all black, a little longer and thinner, and much firmer than *Dendrodoris fumata*, which has a thinner but more extensive mantle skirt. In the two Socotra specimens of *Dendrodoris nigra*, black or dark grey in preservative ten years later, the large white mantle glands are still clearly visible; additionally, in the smaller specimen, there is a substantial and distinct white submarginal band, presumably red in life. The two Maldives specimens of *Dendrodoris nigra* are relaxed and also retain their small white spots (examined 15 years later). The gills and rhinophores are extended and the gill clump is relatively small. The photograph of the only non-black *Dendrodoris nigra* from the Maldives is clearly a juvenile: it had a narrow mantle skirt and was pale translucent red with small white spots and flecks as well as a black marginal band (Plate 58). The gill clump was small (but translucent red), the tri-coloured rhinophores were translucent red basally, black distally, with a large white knob at the tip; these are characters typical of *Dendrodoris nigra*, reviewed by Brodie et al. (1997) based on Pacific specimens. *Dendrodoris fumata* is never black, nor does it have white spots or glands on the dorsum (Brodie and Calado 2006).

### Doriopsilla nigrocera sp. n.

urn:lsid:zoobank.org:act:5ADE579C-AC59-4EBB-8456-0AEE2F204176

http://species-id.net/wiki/Doriopsilla_nigrocera

Fig. 15 Plate 59

#### Material.

Holotype: Persian Gulf (Saudi Arabia): 25 × 17 mm pres., Jubail, Dahwat ad Daffi, 03 April 1992, leg. F Krupp, SMF 337105.

#### Diagnosis.

Creamy yellow elongate oval body with ochre gills and charcoal black rhinophores. Variously sized spiculose tubercles are covered in opaque white lines, with some fainter ones extending from the tubercles to the mantle.

#### Description.

The photograph depicts an elongate oval animal with a wrinkled and damaged margin in life. Dorsum covered in tubercles, themselves covered and sometimes faintly linked to each other with fine white lines. Body was creamy yellow, plumose gills ochre in colour, darker than dorsum, but the rhinophores were black: the stalk was translucent cream and the approximately 15 fine lamellae were charcoal black. The midline on each side and the tip were dusty white. The gut was visible through the skin, opaque creamy orange with black patches.

**Figure 15.**
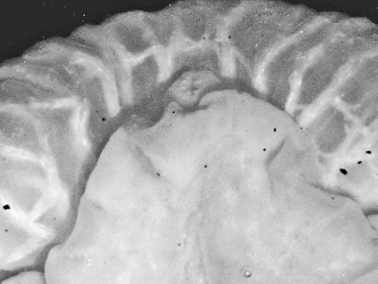
*Doriopsilla nigrocera*sp. n., holotype, ventral view of propodium, head, and oral tentacles.

**Plate 59.**
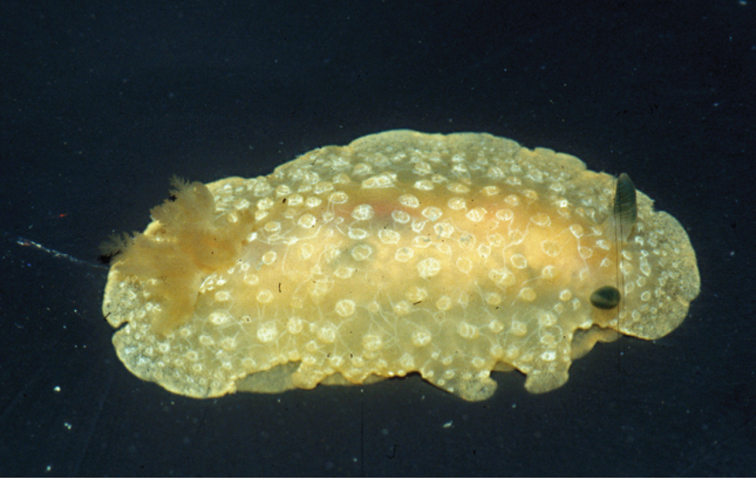
*Doriopsilla nigrocera* sp. n., 25 mm pres., Saudi Arabia, photo: F Krupp.

The preserved specimen has a pink cast and is semi-translucent. Surprisingly, the retracted rhinophores are no longer black, and not visible by translucence through the pockets or the body wall. The tubercles are full of spicules. Tubercles are largest centrally and become progressively smaller toward the margin (Plate 59); in preservative, many are mushroom-shaped. The margin itself is rather thick (and damaged on the right side). Ventrally, the anterior margin of the foot is simple, not bilaminate, and there is a fold under the mouth (Fig. 15).

#### Remarks.

The single specimen is unlike any species of *Doriopsilla* published to date, and different from the photographs available in books and on the internet. The most similar species is *Doriopsilla miniata* (Alder & Hancock), described from India and occurring throughout the Indo-West Pacific in a wide range of colours from nearly white to bright orange. Usually, there are tracings of white lines crossing the dorsum transversely but these may be absent. More critically, the gills and rhinophores of *Doriopsilla miniata* are the same colour as the dorsum or a little darker and this, amongst other characteristics, distinguishes this new species. An un-named species of *Doriopsilla* similar to *Doriopsilla nigrocera* sp. n. is illustrated in Gosliner et al. (2008), Apte (2009), and Apte et al. (2010) in which only the tubercles are covered in white lines; however, this species has rhinophores and gills the same colour as the mantle and not black and may simply be a colour form of *Doriopsilla miniata*. These animals were recorded from South Africa and India, but remain unidentified.

#### Etymology.

The specific epithet refers to the very distinctive black rhinophores, unique to this species of *Doriopsilla*.

### Phyllidia (Fryeria) marindica

(Yonow & Hayward, 1991)

http://species-id.net/wiki/Phyllidia_marindica

Plate 60

Fryeria rueppelii (Rüppell & Leuckart). – Edmunds 1972: 84, fig. 4A (Seychelles) (non *Fryeria rueppelii* Rüppell & Leuckart).
Reyfria marindica Yonow & Hayward, 1991: 23, figs. 10C, D, 13E (Mauritius).
Fryeria marindica . – Brunckhorst 1993: 45, pl. 5B, C (Christmas Island, La Réunion, SW Thailand, and Western Australia); Yonow 1994: 509, fig. 13, tab. 3 (Maldives); Yonow et al. 2002: 860, fig. 16a (Chagos); Yogesh Kumar et al. 2011: 109, fig. 2f (India).
Phyllidia marindica . – Apte 2009: 172, fig. 2u (Laccadive Islands).

#### Material.

Maldives: 35 mm in length (MDV/AB/96/20), 14 m depth, Banana Reef, North Malé Atoll, 10 May 1996, leg. RC Anderson & SG Buttress; photos of several individuals 30-40 mm, March 1999, J Hinterkircher. – La Réunion and Mauritius: numerous photographs of many individuals http://seaslugs.free.fr/nudibranche/a_intro.htm. – Tanzania: photographs of three individuals, Mafia Island, shallow water, March 2004 and May 2009, A de Villiers. – Seychelles: photo of one individual, Lilôt, NW Mahé, 1988-1989, P Kemp.

#### Description/Remarks.

The single specimen is not of the typical western Indian Ocean colour pattern, but more like the animals found in the eastern Indian Ocean and is therefore illustrated here. All other photos are of the typical colour form (as described by Yonow 1994), and range in size from 22 mm to 60 mm in length. The longitudinal pattern of black lines is lacking in this specimen and only transverse rays to the margin are present, connected to each other and crossing the dorsum. There are usually no tubercles on the black lines but if present, these are only small pustules. Ground colour granular blue-white, with more blue pigment present around margin; larger tubercles orange-yellow and arranged in three lines of partially contiguous tubercles along centre of dorsum; in the marginal area, row of single orange-yellow tubercles on each side followed by smaller ones.

### Phyllidia (Fryeria) picta

(Pruvot-Fol, 1957)

http://species-id.net/wiki/Phyllidia_picta

Fig. 16

Phyllidia picta Pruvot-Fol, 1957: 110, figs. 5-12 (no locality).
Fryeria menindie Brunckhorst, 1993: 47, fig. 26B, pl. 4G, 5A (western Pacific).
Fryeria picta . – Yonow 1996: 511, fig. 14, tab. 3 (Christmas Island, Indian Ocean + Indonesia).

#### Material.

Socotra: two specimens, both 27 × 12 mm pres. (St. 018, GR-22), Qualansiyah Bay 12°41.026'N, 53°28.309'E, 10 March 1999, leg. G Reinicke.

#### Description/Remarks.

**Figure 16.**
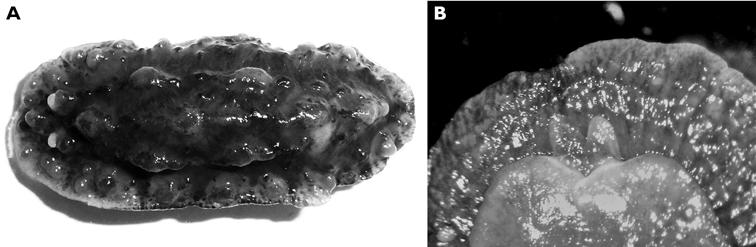
*Fryeria picta*, 27 mm pres. specimen **A** dorsal view **B** ventral view of propodium, head, and tentacles.

This is the first record of *Phyllidia (Fryeria) picta* in the western Indian Ocean, identified by colour pattern and ventral anus. The colour pattern does not appear to differ from that found in the western and central Pacific: the white-in-life areas around the tubercles are now grey-white, and the orange-in-life tubercles are a dirty yellow colour. There are three rows of individual tubercles which are not contiguous (as in *marindica*, see above) although the white bases of two or three tubercles may merge along the central line (Fig. 16A). Ventrally, the anterior foot margin is divided and the oral tentacles are triangular (Fig. 16B). The two specimens differ from preserved specimens of *marindica* (above) and *Phyllidia (Fryeria) rueppelii* Bergh, from the Red Sea: the black pigment covers much more of the dorsum in *Phyllidia (Fryeria) picta*, extending to the margin in rays. The black areas in *Phyllidia (Fryeria) picta* are smooth, while they are tuberculate in *Phyllidia (Fryeria) rueppelii* and small pustules may be present in the black areas of *Phyllidia (Fryeria) marindica*. All three species, however, are similar in having three central lines of orange tubercles, orange rhinophores, and a ventral anus.

### Phyllidia alyta

Yonow, 1996

http://species-id.net/wiki/Phyllidia_alyta

Fig. 17 Plate 61

Phyllidia alyta Yonow, 1996: 500, fig. 9A-G, tab. 2 (Maldives, Mauritius); Apte 2009: 171, fig. 2t (Laccadive Islands); Yogesh Kumar et al. 2011: 111, fig. 3a (India).
Phyllidia elegans . – Eliot 1906: 562 (Maldives) (non *Phyllidia elegans* Bergh).

#### Material.

Maldives: 25 mm in length, Dhigu Tila, near Gulhi, South Malé Atoll, 12 m depth, 14 October 1994, leg. SG Buttress & RC Anderson; 28 × 12 mm, Bathala Island, Ari Atoll, 6 m depth, 28 July 1995, leg. SG Buttress & RC Anderson; 15 × 7 mm pres. (“light grey with black lines, rhinophores and last tubercle yellow”), Bathala Island, Ari Atoll, 10 m depth, 27 July 1995, leg. SG Buttress & RC Anderson; photographs of many individuals, 25-40 mm, March 1997, March 1998, March 1999, J Hinterkircher. – Sri Lanka: photos of two individuals, Negombo, N of Colombo, April 1995, RC Anderson; photos of one individual, Unawatuna, S of Galle, 27 December 2010, S Kahlbrock.

#### Description.

**Figure 17.**
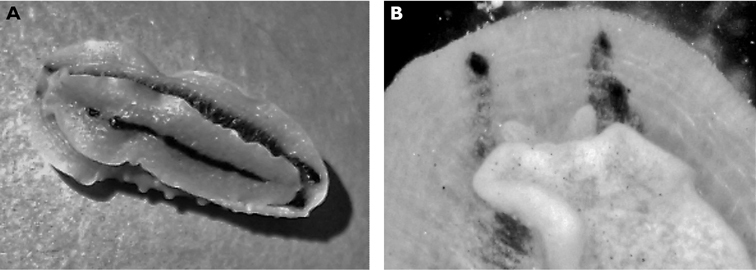
*Phyllidia alyta*, 15 mm pres. specimen **A** ventral view showing line on sole of foot **B** view of propodium, head, and oral tentacles.

**Plate 61.**
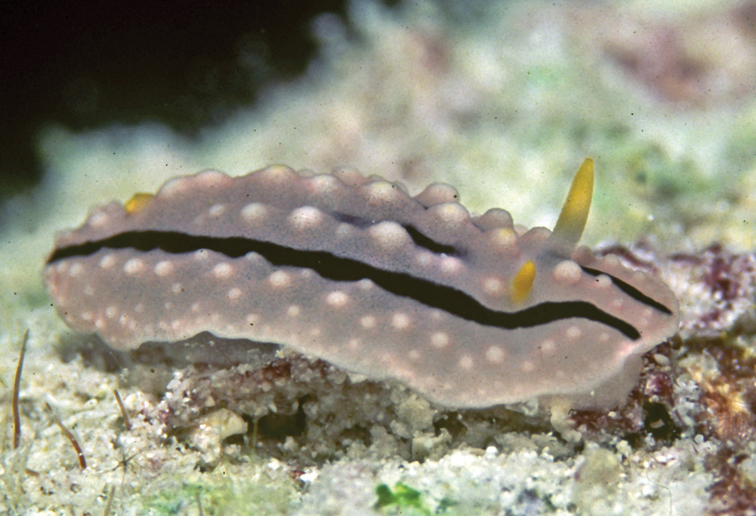
*Phyllidia alyta*, 15 mm, Maldives, photo: RC Anderson.

The preserved 15 mm juvenile is in perfect condition and easily recognised as *Phyllidia alyta*. Broken black line present on sole of foot despite its small size (Fig. 17A), but only two of the four longitudinal dorsal black lines are complete (Plate 61). Three rows of tubercles on midline between start of two lines: central row of tubercles joined by low ridge and only the last tubercle orange. Black crossbar between rhinophores lacking. Two larger specimens typical, with four dorsal black lines, black bar between rhinophores, and ventral broken black line. Foot thickened and concave anteriorly, and triangular tentacles bear groove along each outer side (Fig. 17B). Hyponotum hatched with spicules, dorsal black pigment showing through.

#### Distribution.

It is probable that the species described by Eliot (1906) is *Phyllidia alyta*: he distinguishes it from *Phyllidia varicosa* (he had specimens of both), and *Phyllidia alyta* is the only other species in the Indian Ocean with linearly-arranged orange tubercles and black lines dorsally in combination with a black line on the sole of the foot. This species appears to be restricted to the western Indian Ocean; there are only two internet records from south western Thailand on NudiPixel.

### Phyllidia coelestis

Bergh, 1905

http://species-id.net/wiki/Phyllidia_coelestis

Plate 62

Phyllidia coelestis . – Yonow et al. 2002: 862, fig. 16b (Chagos) and references therein; Apte 2009: 171, fig. 2r (Laccadive Islands).
Phyllidia elegans . – Edmunds 1972: 82, fig. 4b (Seychelles) (non *Phyllidia elegans* Bergh).
Phyllidia varicosa . – Gosliner 1987: 90, fig. 152 (South Africa) (non *Phyllidia varicosa* Lamarck).
Phyllidia alia Yonow, 1984: 224 figs. 6C, D, 7A, 8F, G (Sri Lanka).

#### Material.

Madagascar: 65 × 25 mm (PK-C), Ampangorina, Nosy Komba, 1 m depth on *Acropora*, 30 January 1992, leg. P Kemp. – Tanzania: photographs of two individuals, Mafia Island, shallow water, 15 January 2005 and 04 July 2005, A de Villiers. – Seychelles: 32 × 12 mm (PK-FF), Lilôt, NW Mahé, 15 m on encrusted coral, 26 April 1992, leg. P Kemp; 32 × 15 mm preserved (NHMUK acc. no. 2222), slightly curled, east side of East Channel, Aldabra, 1 m depth in coral, 25 September 1967, leg. JD Taylor; 49 × 15 mm preserved (NHMUK acc. no. 2222), Passe Femme, Aldabra, in shallow water beneath coral, 29 November 1967, leg. JD Taylor. – Maldives: 23 × 11 mm preserved (NHMUK ref. M/02/B/42), Gan, 04 September 1964, PSD Maldive Islands Expedition. – La Réunion and Mayotte: numerous individuals photographed, 20-45 mm in length http://seaslugs.free.fr/nudibranche/a_intro.htm.

#### Description.

Ground colour granular blue-white with three black lines: median line containing orange-tipped tubercles, two smooth lateral lines. Lateral lines normally meet anteriorly, and extend to margin usually as U-shape, but remain separated posteriorly. Outside the black lines, mantle bears tubercles, black flecks, and smaller scattered pustules toward edge; larger tubercles may have orange tips. Rhinophores bright orange, up to 16 lamellae; row of orange tubercles originates behind each rhinophore. Ventrally, propodium notched or deeply concave; all specimens have long tapering oral tentacles, grooved laterally and bearing black pigment on dorsal surfaces extending ventrally onto bases.

#### Distribution/Remarks.

These specimens and photographed individuals belong to the typical form of *Phyllidia coelestis*, a commonly recorded species in the western Indian Ocean and occurring as far south as South Africa. Another group of specimens also identified as *Phyllidia coelestis* (Brunckhorst 1989, 1993; Yonow 2011 as dark form) has a central oval region where the ground colour is black and only a marginal band around it is blue-white. The edge of the central black area is irregular and may form short rays extending a little way into the blue-white margin, identical to that of *Phyllidia (Fryeria) picta* (p. 56), but this form has not been found in the western Indian Ocean.

### Phyllidia exquisita

Brunckhorst, 1993

http://species-id.net/wiki/Phyllidia_exquisita

Fig. 18 , Plate 63

Phyllidia exquisita Brunckhorst, 1993: 38, pl. 3C (SW Thailand + W Pacific); Debelius and Kuiter 2007: 267 (Maldives).

#### Material.

Maldives: single specimen 15 mm (11 × 6 mm pres.), Dhigu Tila, near Gulhi, South Malé Atoll, 12 m depth, 14 October 1994, leg. SG Buttress & RC Anderson. – Madagascar: photo of one individual, Nosy Bé, 12-14 m depth, 25 July 2010, S Bachel (http://seaslugs.free.fr/nudibranche/a_intro.htm). – Mayotte: photo of one individual, Ilot Blanc de Mtsamboro, 4 m depth, 30 Dec 2010, M Deuss (http://seaslugs.free.fr/nudibranche/a_intro.htm).

#### Description.

**Plate 63.**
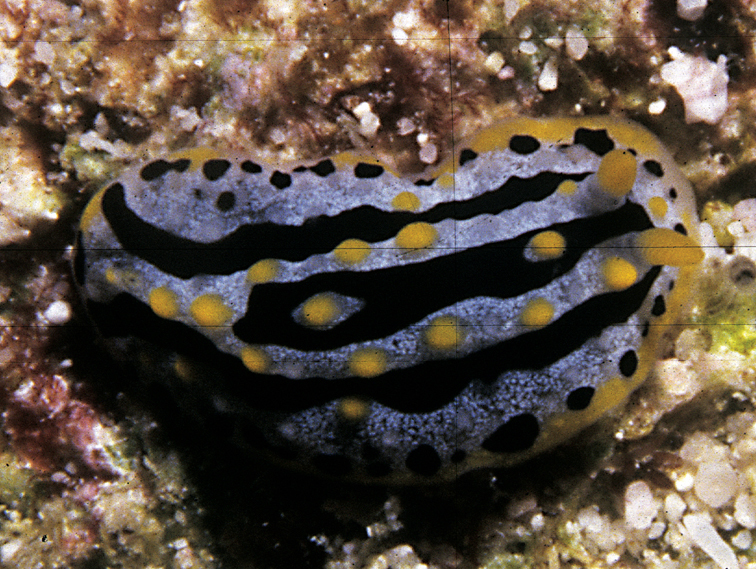
*Phyllidia exquisita*, 15 mm, Maldives, photo: RC Anderson.

Elongated oval animal with granular white ground colour and three black lines connected anteriorly around rhinophores but not posteriorly; central one shortest and widest. Tubercles present in black areas. Three main rows of orange-tipped tubercles in life, middle ones located along central black line, lateral ones between black lines. Several isolated orange-tipped tubercles outside black lines within marginal band of granular white, which also contains non-orange tubercles. Ring of rounded black spots at edge of granular white and bordering the orange margin. Rhinophores same orange colour as tubercles and margin; anal papilla faintly orange. When the colour images of the living specimen are magnified, a completely separate band is visible around the margin, which is translucent when not pigmented with orange (Plate 63).

**Figure 18.**
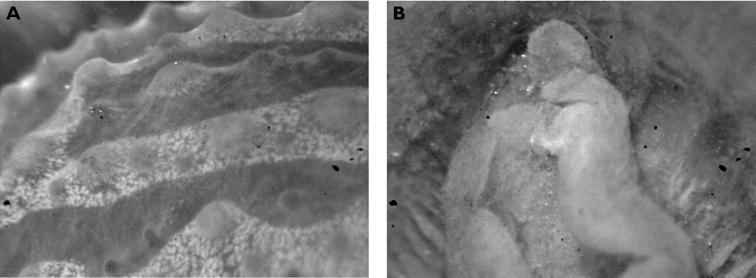
*Phyllidia exquisita*, 11 mm pres. specimen **A** dorsal view showing granular white pigmentation and spiculose black areas **B** ventral view of propodium, head, and oral tentacles (left oral tentacle folded over).

The preserved specimen does not retain any orange pigmentation. The tubercles located on the black lines are clearly spiculose, while those located on white areas are covered by granular pigment (Fig. 18A). The anus is very difficult to see, located far posteriorly and not on a tubercle. Ventrally, the anterior margin of the foot appears to be concave; the oral tentacles are conical (Fig. 18B).

#### Distribution.

Brunckhorst (1993) described this species from one juvenile specimen (15 mm) from the eastern Indian Ocean and nine specimens (11-30 mm) from the West Pacific but it has been recorded to 45 mm in eastern Australia (Cobb and Willan 2006). This first record from the Maldives records its distribution in the western Indian Ocean, and photographs from the Maldives and Kenya are available on NudiPixel. *Phyllidia schupporum* Fahrner & Schrödl (2000) was originally described from the Red Sea and compared to *Phyllidia exquisita*; it is the most similar species but it has very high spiculose tubercles (see also Yonow 2008). The numerous photographs now available on the internet show that there are several similar species but, of those with three to four black lines and granular white colour pattern most similar to the holotype of *Phyllidia exquisita*, variations occur from no orange margin to fragmented and complete orange margins, and these can in all probability be assigned to *exquisita*. Until specimens of the other illustrated forms are examined, their identities must remain unconfirmed.

### Phyllidia koehleri

Perrone, 2000

http://species-id.net/wiki/Phyllidia_koehleri

**Figure 19.**
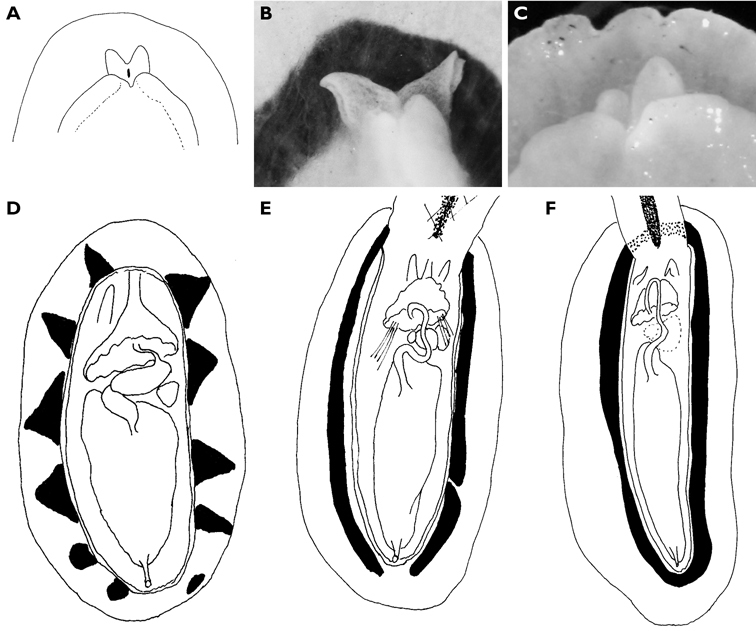
*Phyllidia koehleri* **A** ventral view of propodium, head, and oral tentacles of 11 mm pres. specimen. **B** ventral view of propodium, head, and oral tentacles of 14 mm pres. specimen **C** ventral view of propodium, head, and oral tentacles of 17 mm pres. specimen **D** digestive anatomy of 11 mm pres. specimen **E** digestive anatomy of 14 mm pres. specimen **F** digestive anatomy of 17 mm pres. specimen.

**Plate 64.**
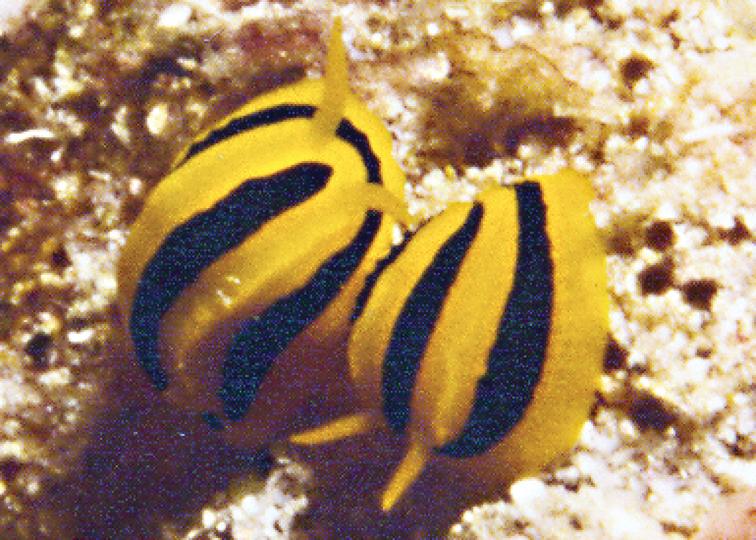
*Phyllidia koehleri*, 10 mm, Maldives, photo: J Hinterkircher.

**Plate 65.**
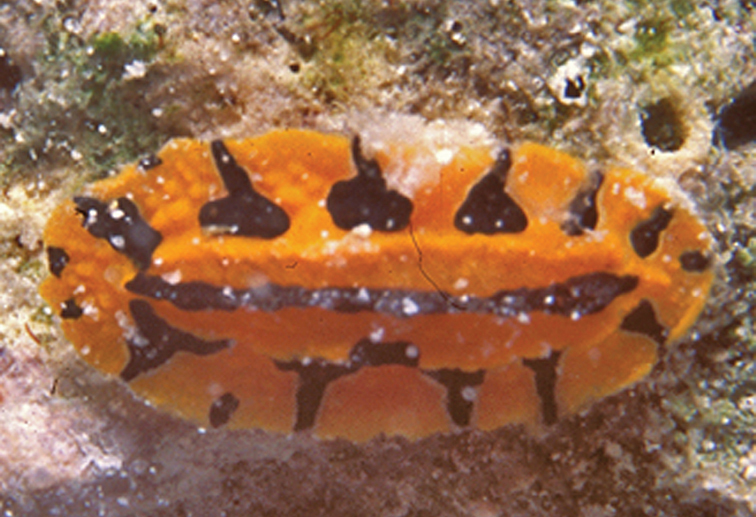
*Phyllidia koehleri*, 11 mm pres., Maldives, photo: N Yonow.

Fig. 19 , Plates 64 , 65

Phyllidia koehleri Perrone, 2000: 89, figs. 1-4 (Maldives).

#### Material.

Maldives: 11 × 6 mm pres. (NY-142, orange with black triangles), Fulidhoo House Reef, Felidhoo Atoll, 20 m depth, 12 January 1991, leg. N Yonow; 14 × 7 mm pres. (SH-65, “bright yellow” with black ring), Maaya Tila, Ari Atoll, 16 m depth, August 1991, leg. S Harwood; 17 × 7 mm pres. (“yellow with black” ring), Hans’ Place, North Malé Atoll, 23 m depth, 3 November 1994, leg. SG Buttress & RC Anderson; photographs only of two individuals together, 10 mm approx. lengths (yellow with black ring), 12 m depth, July 1992, J Hinterkircher.

#### Description.

*Phyllidia koehleri* is a small species (the largest recorded size is 17 mm preserved length, above), bright yellow or orange, with a central longitudinal black line and a black ring, solid or broken, encompassing the anus and rhinophores. Body oval with single raised ridge behind each rhinophore, either side of central black line. Apart from two strong central ridges and tiny single tubercles covering skirt, the mantle is smooth. Broad central black band extending from behind the rhinophores to the anus, black ring then encloses central area (Plate 64); colour in life of these specimens yellow. Sometimes, as in one specimen, the black ring is broken into dashes which form bases of black triangular rays extending to margin (Plate 65); colour of this specimen orange but both patterns occur in both colours in images on the internet. There may be a few black spots in the marginal band and the orange form appears more tuberculate than the yellow ringed form. Rhinophores same colour as body, yellow or orange depending on body colour, extremely long and thin, bearing little more than 10 diagonal lamellae. Anal papilla translucent, extending from raised rim at junction of two ridges very near the posterior border, surrounded by some small tubercles. Ventrally, foot white in life, but hyponotum and gills yellow or orange in life. Anterior margin of foot notched, head white, and large oral tentacles triangular, grooved on outer margins, tipped with orange in life (Fig. 19A-C).

Internally, all specimens are similar, and illustrated here (Fig. 19D-F). The pharyngeal bulb is conical and slightly asymmetrical. Its posterior surface is glandular, the ventral projections a little longer than the dorsal ones. The oesophagus is very long, exiting the bulb posteriorly and bending anteriorly to pass through the nerve ring sitting on the bulb.

#### Distribution/Remarks.

This small species appears to be endemic to the Maldives; a dozen photographs on various websites are recorded only from the Maldive archipelago. Colour variation appears standard: either animals have a black ring or they have black triangles, and both patterns occur in yellow and orange. Although they appear rather different, they are consistent in external (two strong ridges, scattered marginal tubercles, rhinophores with few diagonal lamellae) and internal (asymmetrical buccal bulb, a long oesophagus bending anteriorly before passing through the nerve ring) morphologies.

### Phyllidia multituberculata

Boettger, 1918

http://species-id.net/wiki/Phyllidia_multituberculata

Fig. 20 , Plate 66

Phyllidia multituberculata Boettger, 1918: 129, pl. 8 figs. 4a–c (Aru Islands, Indonesia); Yonow 1996: 487, figs. 2A–E, 3A–G, 4B, tab. 1 (Seychelles and Mauritius).
Phyllidia ocellata . – Yogesh Kumar et al. 2011: 111, fig. 3b (India) (non *Phyllidia ocellata* Cuvier).

#### Material.

Typical form: Socotra: 30 × 16 mm pres. (St. 064, SAM1), Quatub Bay, 18 March 1999, leg. S Al-Moghrabi.

Black form: Maldives: single specimen 14 × 8 mm crawling (NY-167), Kudhiboli Tila, Felidhoo Atoll, 21 m depth, 15 January 1991, leg. N Yonow; two specimens together 28 × 20 mm and 30 × 21 mm both preserved, Maayafushi House Reef, Ari Atoll, 34 m depth, 18 December 1991, leg. H Voigtmann; 14 mm (MDV/AB/96/2), Cocoa House reef, South Malé Atoll, 16 m depth, 01 May 1996, leg. RC Anderson & SG Buttress; photos of one individual, 1986-1994, J Hinterkircher. – Sri Lanka: two pres. specimens 21 × 15 mm and 25 × 18 mm and photographs of a juvenile, Unawatuna, S of Galle, 26-28 December 2010, S Kahlbrock.

#### Description.

**Figure 20.**
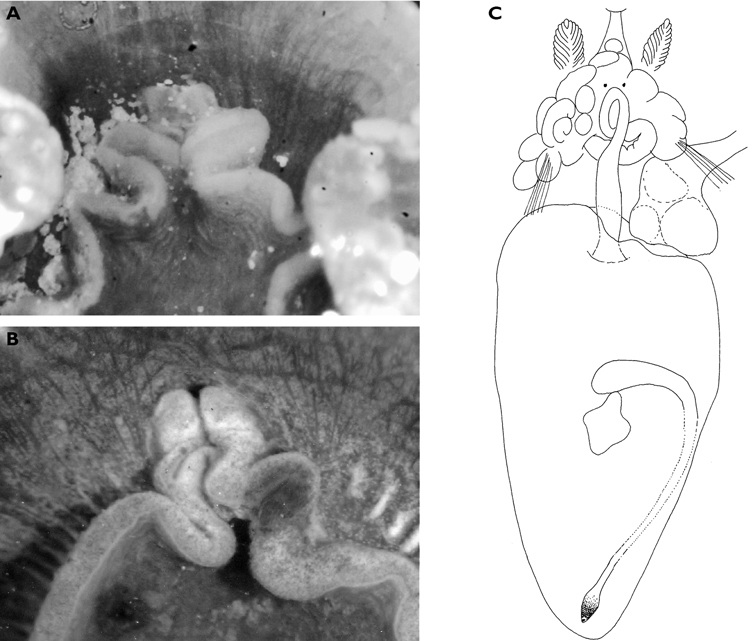
*Phyllidia multituberculata* **A** ventral view showing propodium, head, and oral tentacles, 30 mm pres. Socotra specimen **B** ventral view showing head and oral tentacles, 28 mm pres. Maldives specimen **C** anatomy of 28 mm pres. Maldives specimen.

The Socotra specimen is of the typical form described and illustrated by Boettger, with five black patches on each side. This is the commonly occurring species in the Indian Ocean; see Yonow (1996) for descriptions, illustrations, and discussions. The white ring surrounding the black patch is visible in the preserved specimen and the oral tentacles are illustrated for comparison (Fig. 20A). The anterior margin of the foot is bilaminate, the head is visible, and the conical oral tentacles have a groove on their outer sides.

**Plate 66.**
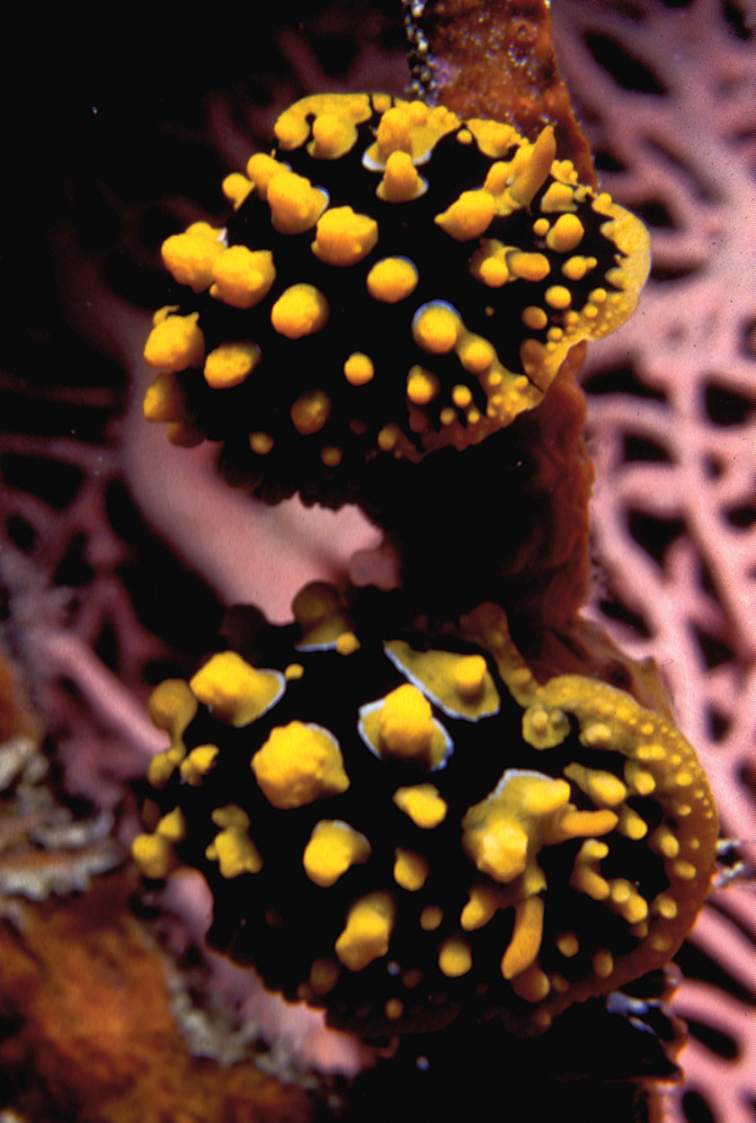
*Phyllidia multituberculata* black form, 28 and 30 mm pres., Maldives, H Voigtmann.

The Maldives and Sri Lankan specimens differ in colour pattern from the Socotra specimen but are identical to each other, completely black with numerous orange mushroom-shaped tubercles: some had a white ring at the base when alive (Plate 66), and still visible in the preserved specimens. Tubercles vaguely organized in longitudinal rows over dorsum, tubercles in central row largest. Rhino-tubercles present as well as single very large tubercle behind and between them. Margin orange, somewhat scalloped along inner margin bordering black, with single small tubercles aligned along edge. In Sri Lankan specimens, white submarginal line. Rhinophores deeper shade of orange than tubercles and margin, raised pocket rims also orange. Ventrally, foot of black and orange specimens deeply divided (convoluted for its entire margin in the largest specimen), head bipartite, separated oral tentacles digitiform (Fig. 20B), most similar to those of *Phyllidia multituberculata* form *undula* (fig. 5B in Yonow 1996). In smaller, more relaxed, Maldive specimen (NY-167), thickened anterior margin of foot concave, head barely visible, oral tentacles conical.

#### Remarks.

Dissection of the 28 mm Maldive animal shows that the digestive anatomy is typical for the genus (Fig. 20C). Significantly, it has a segmented asymmetrical pharyngeal bulb as described and illustrated for *Phyllidia multituberculata* and *Phyllidia multituberculata* form *undula*. This feature, in addition to the colour patterns, separates the Indian Ocean *Phyllidia multituberculata* from the Pacific *Phyllidia ocellata* Cuvier, which has a symmetrical pharyngeal bulb (e.g. Yonow 1996, Domínquez et al. 2007, Yonow 2011). Molecular studies are revealing cryptic species with more limited distributions for what were previously thought to be widely distributed variable species (e.g. for *Bulla* see Malaquias and Reid 2008, for *Melanochlamys* see Krug et al. 2008, and for *Thuridilla* see Carmona et al. 2011), and it is probable that molecular studies will reveal many more phyllidiid species than are currently accepted. It is therefore preferable to describe geographical and colour forms separately and clearly.

### Phyllidia varicosa

Lamarck, 1801

http://species-id.net/wiki/Phyllidia_varicosa

Phyllidia varicosa . – Edmunds 1971: 388, fig. 23 (Tanzania); Edmunds, 1972: 75 (Seychelles); Rao et al. 1974: 119, fig. 2a, b (Laccadive Islands); Fahrner and Schrödl 2000: 164, figs. 1-4 (Red Sea and Indonesia); Apte 2009: 171, fig. 2s (Laccadive Islands); Richmond 2011: 282 (East Africa); Yogesh Kumar et al. 2011: 111, fig. 3c (India).
Phyllidia arabica Ehrenberg. – Yonow 1996: 495, figs. 7A-F, 8A-C, tab. 2 and references therein (Indian Ocean).
non Phyllidia varicosa . – Gosliner 1987: 90, fig. 152 (South Africa) (= *Phyllidia coelestis* Bergh).

#### Material.

Socotra: two pres. specimens 65 × 31 mm and 42 × 12 mm curled, (St. 064, SAM1), Quatub Bay, 18 March 1999, leg. S Al-Moghrabi; single pres. specimen 48 × 18 mm (St. 063, GR-57), 12°38.945'N, 53°56.028'E, off Quatub fishing village, in front of airport, 18 March 1999, leg. G Reinicke; 33 × 15 mm pres. (St. 068, MAP-138), 12°41.062'N, 54°04.508'E, Hawlaf, off jetty, 19 March 1999, leg. M Apel. – South Africa: three specimens (Natal Museum refs 51894, 5908). – Kenya: 35 × 15 mm and 15 × 8 mm both preserved (completely bleached dorsally and ventrally, notch on foot anteriorly), Turtle Bay, subtidal on rock, July 1969, leg. K Brander (nudibranch XII, TE Thompson collection, NHMUK acc. no. 2364). – Tanzania: photographs of two individuals, 1994, MD Richmond; photographs of four individuals, M’Nazi Bay, Msimbati, near Mtwara, May 1994, IM Horsfall; photographs of several individuals, Mafia Island, shallow water, 12 February 2004 and 06 January 2008, A de Villiers. – Zanzibar: photo of one individual, MD Richmond. – Mauritius: 65 × 32 mm pres. (dissected), MNHN, Paris (no label other than ‘Mauritius’). – La Réunion, Mauritius, Rodrigues, and Mayotte Islands: http://seaslugs.free.fr/nudibranche/a_intro.htm. – Seychelles: 53 × 25 mm pres., Victoria Harbour, 12 May 2005, leg. MD Richmond; photo of one individual, Lilôt, NW Mahé, 1988-1989, P Kemp. – Maldives: numerous individuals documented and/or photographed but not preserved, 8-80 mm, Maldives Expedition, N Yonow (see Yonow 1994, 1996) and 1997-1999 by J Hinterkircher. – Sri Lanka: photos of two individuals (1 adult & 1 very small juvenile), Negombo, N of Colombo, April 1995, RC Anderson; photos of two large individuals, Negombo, N of Colombo, 16 January 2011, S Kahlbrock.

### Phyllidiella meandrina

(Pruvot-Fol, 1957)

http://species-id.net/wiki/Phyllidiella_meandrina

Fig. 21 , Plate 67

Phyllidia (Phyllidiella) nobilis . – Eliot 1904: 282, pl. 16 fig. 1 (East Africa) (non *Phyllidiella nobilis* Bergh).
Phyllidia meandrina Pruvot-Fol, 1957: 113 (Mauritius).
Phyllidiella meandrina . – Yonow & Hayward 1991: 21, figs. 10E, 13B (Mauritius); Yonow et al. 2002: 863, fig. 16d (Chagos).
Phyllidia sp. 1. – Gosliner 1987: 90, fig. 153 (South Africa).

#### Material.

Socotra: 40 × 21 mm curled, pres. (St. 018, GR-23), Qualansiyah Bay 12°41.026'N, 53°28.309'E, 10 March 1999, leg. G Reinicke. – Kenya: three pres. specimens 26 × 15 mm (bent and curled), 17 × 11 mm, and 17 × 8 mm (slightly curled), Vipingo, 25 miles N of Mombasa, rock pool ELW, 23 September 1984, leg. J Hognerud; four pres. specimens curled and faded, approx. 15 mm, Kibirijini, intertidal pool, 27 August 1969/ nudibranch IXX, Turtle Bay, subtidal rock, 12 July 1969/nudibranch XXIII, Mida 30’ rock, 25 July 1969/nudibranch XX, Turtle Bay, intertidal rock, July 1969, all leg. K Brander (TE Thompson coll. NHMUK acc. no. 2364). – Tanzania: photographs of two individuals, Mafia Island, shallow water, May 2009, A de Villiers; photographs of two individuals, M’Nazi Bay, Msimbati, near Mtwara, May 1994 and May 1995, IM Horsfall. – Zanzibar: photo of few individuals, MD Richmond and A de Villiers. – South Africa: two specimens (Natal Museum specimen ref. nos. S1773 and E4898 + photo slide ref. 0061). – Madagascar: 40 × 15 mm (PK-D2), Ampangorina, Nosy Komba, 1 m depth (“cruising over sand”), 30 January 1992, leg. P Kemp; 50 × 15 mm (PK-E), Ampangorina, Nosy Komba, 1 m depth (“on algal-encrusted stag horn coral”), 20 February 1992, leg. P Kemp. – Sri Lanka: 47 × 17 mm pres. (curled), via Tropical Marine Centre, London (fish importers), 14 August 1990. – Mauritius: photos of several individuals, M Parmantier, and La Réunion http://seaslugs.free.fr/nudibranche/a_intro.htm.

**Figure 21.**
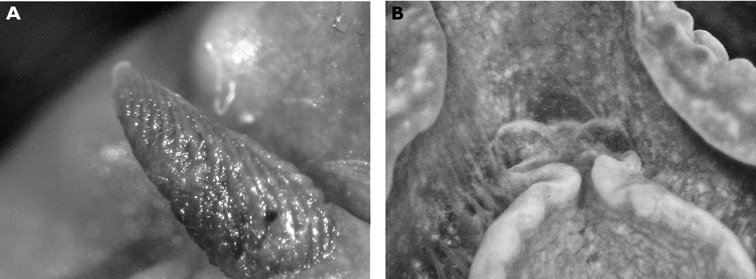
*Phyllidiella meandrina* **A** left rhinophore of 40 mm pres. Socotra specimen **B** ventral view of 50 mm Madagascar specimen showing propodium and rounded black oral tentacles.

#### Description.

The Socotra specimen is an almost perfect replica of the type specimen, with two incomplete median circles of small tubercles. First lateral encircling band of tubercles composed of small tubercles followed by row of single isolated tubercles and narrow black line before pink margin; long black rhinophores (Fig. 21A). Large fused oral tentacles tinged with black in life; pigment still remains on Madagascar specimens 20 years later (Fig. 21B).

#### Remarks.

Although easily recognized, *Phyllidiella meandrina* is not a common species in the western Indian Ocean; it has not been recorded from the Maldives (contrary to my statement in Yonow et al. 2002: 864). Eliot’s (1904) species recorded as *Phyllidiella nobilis* from East Africa is here identified as *Phyllidiella meandrina*: his description and illustrations of the outer two to three rows of tubercles are typical of *Phyllidiella meandrina*.

### Phyllidiella pustulosa

(Cuvier, 1804)

http://species-id.net/wiki/Phyllidiella_pustulosa

Plate 68

Phyllidiella pustulosa . – Fahrner and Beck 2000: 201, pl. 2 fig. 3 (Pacific); Yonow 2008: 220 (Red Sea); Apte 2009: 172, fig. 2v (Laccadive Islands); Yogesh Kumar et al. 2011: 111, fig. 3d (India).
Phyllidia nobilis Bergh, 1869. – Yonow and Hayward 1991: 21, figs. 11f–h (Mauritius).

#### Material.

Socotra: 28 × 9 mm curled pres. (IT-157, RJ-011), 12°18.698'N, 53°48.285'E, 09 April 1999, leg. R Janssen. – Little Aden: 40 × 17 mm preserved (NHMUK), curled at edges, BP Refinery, PO Box 3003, 05 February 1968, leg. Miss Gobnait Murphy. – Seychelles: preserved (NHMUK acc. no. 2222), Passe Femme, Aldabra, shallow water beneath coral, 29 November 1967, leg. JD Taylor. – Sri Lanka: 17 × 12 mm pres., near Pigeon Island, Nilaveli, Trincomalee, 11 March 1995, leg. SG Buttress & RC Anderson; photo of 1 individual, Negombo, N of Colombo, April 1995, RC Anderson; photos of one individual, Negombo, N of Colombo, 11 January 2010, S Kahlbrock. – Tanzania: photo of one individual, 1994, MD Richmond. – Andaman Islands: one pres. specimen (NHMUK 1960.1334, acc. no. 1838), Winckworth coll. – Christmas Island: 25 × 11 mm pres. (NHMUK 1899.5.26.12), Flying Fish Cove, leg. Sir J Murray (Andrews).

### Phyllidiella rosans

(Bergh, 1873)

http://species-id.net/wiki/Phyllidiella_rosans

Fig. 22 Plate 69

Phyllidiella rosans . – Brunckhorst 1993: 56, pl. 6C, D (Maldives, La Réunion, Mauritius); Yonow et al. 2002: 864, figs. 16e, 17a (Chagos); Apte 2009: 172, fig. 2w (Laccadive Islands).

#### Material.

Maldives: two pres. specimens 52 × 16 mm and 27 × 16 mm (with 3 median lines and 2 rings), Gangehi Island lagoon, Ari Atoll, 1-5 m depth, February 1988, leg. G Corriero; 16 × 11 mm (3 median lines and 2 rings), Gangehi Island lagoon, Ari Atoll, < 5 m depth, August 1990, leg. Cabona; 43 × 14 mm (NY-4 “green ridges, tubercles in 3 median lines + 3 rings, long black anal papilla, long black rhinophores”), Bodhuhithi Channel, North Malé Atoll, 21.5 m depth, 11 May 1990, leg N Yonow; 21 × 14 mm pres., curled (NY-19 “3 median line of tubercles + 3 rings”), Rasfaree outer reef, North Malé Atoll, 27 m depth, 13 May 1990, leg. N Yonow; 34 × 14 mm (NY- 24 “pink edge, smelly”), Kuramathi, Rasdhu Atoll, 20 m depth, 13 May 1990, leg. N Yonow; 33 × 17 mm (NY- 62) and 21 × 17 mm (NY- 63), Kandholudhu Tila, Ari Atoll, 10 m depth, 15 May 1990, leg. N Yonow; 21 × 8 mm (NY-103 “green with 3 rings”), Maaya Tila, Ari Atoll, 11 m depth, 18 May 1990, leg. N Yonow; 48 × 15 mm (NY-119 “crests in 3 lines + 2 rings, produced slimy mucus”), Tin Tila, Ari Atoll, 27 m depth, 21 May 1990, leg. N Yonow; 22 × 11 mm (NY-126 “median ridge + 3 rings”), Maayafushi Tila, Ari Atoll, 46 m depth, 22 May 1990, leg. N Yonow; 45 × 18 mm (NY-145 “green, separated grey oral tentacles, sole of foot mottled black concentrated centrally, smelly, exuded white mucus”) and 40 × 16 mm (NY-146), Fulidhoo house reef, Felidhoo Atoll, 20 m depth, 12 January 1991, leg. N Yonow; four specimens 33 × 16 mm (NY-159), 30 × 11 mm (NY-160), 18 × 15 mm (NY-161) (all “green and black ringed”), 25 × 13 mm (NY-162, “pink & black, very ridged, black rhinophores”), Kunarvashi Tila, Felidhoo Atoll, 22 m depth, 14 January 1991, leg. N Yonow; six specimens 30 × 15 mm pres. curled (“3 median + 3 rings”), 42 × 18 mm pres. (“3 medians + 3 rings”), 28 × 12 mm pres. (“3 medians slightly broken”), 26 × 12 mm pres. (“3 medians + 2 rings”), 24 × 11 mm pres., 22 × 10 mm pres. (all as NY dive 42), Maaya Tila, Ari Atoll, 33 m depth, 19 January 1991, leg. N Yonow; 21 × 5 mm (NY- 208), Manta Point, North Malé Atoll, 17 m depth, 21 January 1991, leg. N Yonow; numerous individuals photographed & measured but not collected, 21-52 mm, Maldives Expedition, N Yonow (see above and Yonow 1994, 1996); photographs only of many individuals, 1986-1994, J Hinterkircher. – Tanzania: photographs of numerous individuals, M’Nazi Bay, Msimbati, near Mtwara, May 1994, May 1995, IM Horsfall. – Mauritius and La Réunion: numerous photographs, 18-35 mm, http://seaslugs.free.fr/nudibranche/a_intro.htm.

**Figure 22.**
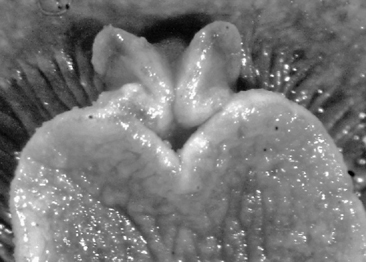
*Phyllidiella rosans*, 42 mm (NY dive 42), view of propodium, head, and black pigment on oral tentacles.

### Phyllidiella rudmani

Brunckhorst, 1993

http://species-id.net/wiki/Phyllidiella_rudmani

Fig. 23 Plate 70

Phyllidiella rudmani Brunckhorst, 1993: 62; fig. 29d, pl. 7C, D (SW Thailand + Pacific); Yonow 1996: 504, fig. 11A-G (Maldives).

#### Material.

Maldives: 10 × 3 mm alive (NY-11a), Nakatcha Tila, North Malé Atoll, 20 m depth, 12 May 1990, leg. N Yonow.

#### Description/Distribution.

**Figure 23.**
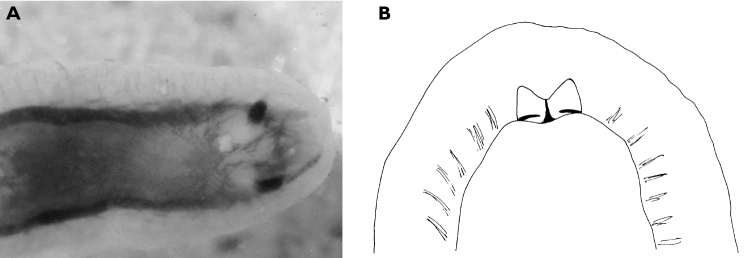
*Phyllidiella rudmani*, juvenile 10 mm specimen **A** dorsal view showing black markings, lack of tubercles, and spicule arrangement **B** ventral view showing propodium, head, and oral tentacles.

This juvenile specimen has identical marking to adult specimens, white with two black lines; dorsum completely smooth, tubercles not yet developed and spicules clearly visible in skin (Fig. 23A). Ventrally, minute tentacles same shape as in adults (Yonow 1996, figs. 11C, D), but no black pigment (Fig. 23B); gills and crevice between foot and hyponotum still black. *Phyllidiella rudmani* has a wide distribution from the eastern Indian Ocean to Japan in the western Pacific; it is currently only known from the Maldives in the central Indian Ocean.

### Phyllidiella striata

(Bergh, 1889)

http://species-id.net/wiki/Phyllidiella_striata

Fig. 24 , Plate 71

Phyllidiopsis striata Bergh, 1889: 866, pl. 84 figs. 23, 24 (SW Thailand); Eliot 1906a: 563 (Maldives).
Phyllidia nobilis . – Edmunds 1972: 79 (part), fig. 3A, F (Seychelles) (non *Phyllidiella nobilis* Bergh).
Phyllidia empelia Yonow, 1984: 223, figs. 6, 7B, 8A, B (Sri Lanka).
Phyllidia mediocris Yonow & Hayward, 1991: 20, figs. 11C-E, 13A (Mauritius).
Phyllidiella striata . – Yonow et al. 2002: 866, figs. 16f, 17b, 18a (Chagos).

#### Material.

Maldives: 22 × 11 mm pres., Gan, Maldives Island Expedition, 20 m depth, 28 July 1964, leg. PSD (NHMUK ref. no. M/01/0); 45 × 11 mm (NY-2, “tuberculate ridges, grey oral tentacles with grooves”), Blue Cave, North Malé Atoll, 25 m depth, 11 May 1990, leg. N Yonow; 47 × 9 mm (NY-9), Nakatcha Tila, North Malé Atoll, 27 m depth, 12 May 1990, leg. N Yonow; 26 × 10 mm (NY-17), Nakatcha Kura Tila, North Malé Atoll, 12 m depth, 12 May 1990, leg. N Yonow; three specimens 20 × 7 mm, 21 × 7 mm, and 22 × 8 mm (NY-20, 21, 22, “sole grey cf. *rosans* black”), Rasfaree Outer Reef, North Malé Atoll, 27 m depth, 13 May 1990, leg. N Yonow; four specimens 34 × 10 mm (NY-25, “broken crests, 2 rings, ring around anus, grey oral tentacles”), 34 × 13 mm (NY-26, “similar to NY-25 but crests more continuous, distinctly elongated”), 21 × 6 mm (NY-28, “median line + laterals + ring, metapodium with black median line, oral tentacles grey with black tip,”), 16 × 4 mm (NY-29, “black with broken med + laterals + 1 ring tubercles, oral tentacles grey pink”), Kuramathi, Rasdhu Atoll, 20 m depth, 13 May 1990, leg. N Yonow; six specimens 30 × 10 mm (NY- 48), 24 × 7 mm (NY- 50), 20 × 5 mm (NY- 56), 23 × 6 mm (NY- 57), 33 × 10 mm (NY- 58), 11 × 5 mm (NY- 66, “black with median + 2 lateral + 1 ring, pink tubercular crests”), Kandholudhu Tila, Ari Atoll, 10 m depth, 15 May 1990, leg. N Yonow; three specimens all pres. 16 × 10 mm (bent), 22 × 10 mm, 25 × 8 mm (NY dive 11, “pink & black crested”), Kandholudhu fringing reef, Ari Atoll, max 25 m depth, 15 May 1990, leg. N Yonow; three specimens pres. 22 × 8 mm bent, 22 × 10 mm curled, 25 × 10 mm (NY dive 12), Maayafushi fringing reef, Ari Atoll, max 30 m depth, 16 May 1990, leg. N Yonow; two specimens 26 × 10 mm (NY-104, “pink crested”) and 12 × 6 mm (NY-105), Maaya Tila, Ari Atoll, 11 m depth, 18 May 1990, leg. N Yonow; 15 × 7 mm pres. (NY-108), Toroca, Ari Atoll, 10 m depth,19 May 1990, leg. N Yonow; 24 × 10 mm (NY-127), Maayafushi House Reef, Ari Atoll, 46 m depth, 22 May 1990, leg. N Yonow; 24 × 10 mm (NY-141), Small Banana Reef, North Malé Atoll, 25 m depth, 10 January 1991, leg. N Yonow; 20 × 7 mm (NY-147), Felidhoo House Reef, Fulidhoo Atoll, 20 m depth, 12 January 1991, leg. N Yonow; two specimens 24 × 18 mm (NY-149) and 24 × 7 mm (NY-150, “pink crested, 2 rings, smelly & exuded slime”), Fulidhoo House Reef, Felidhoo Atoll, 20 m depth, 12 January 1991, leg. N Yonow; 25 × 10 mm (NY-179, “pink crested”), Dhageti Tila, Ari Atoll, less than 17 m depth, 16 Jan 1991, leg. N Yonow; four specimens 40 × 13 mm (NY-199), 40 × 13 mm (NY-200), 25 × 10 mm (NY-201), 35 × 14 mm (NY-202), Bathala Faro, Ari Atoll, 15 m depth, 17 January 1991, leg. N Yonow; two specimens pres. 17 × 7 mm and 18 × 8 mm (NY dive 42, “pink broken crested”), Maaya Tila, Ari Atoll, 33 m depth, 18 January 1991, leg. N Yonow; 45 × 17 mm (NY-203, “pink broken crested”), Maayafushi lagoon, Ari Atoll, 22 m depth, 18 Jan 1991, leg. N Yonow; 13 × 5 mm (NY-205, “pink crested”), Bodhu Fahlu, Ari Atoll, 15 m depth, 19 Jan 1991, leg. N Yonow; 30 mm length (SH-6, “3 broken median lines, edge of mantle white”), Huravalhi Reef, Lhaviyani Atoll, 20 m depth, 1 March 1991, leg. S Harwood; 18 × 8 mm pres. (SH-55), Foteo West Reef, Felidhoo Atoll, 20 m depth, 25 April 1991, leg. S Harwood; 12 × 7 mm pres., Naifaro House Reef, Lhaviyani Atoll, 29 m depth, 3 November 1991, leg. H Debelius; 16 mm, Bathala Island, Ari Atoll, 10 m depth, 27 July 1995, leg. SG Buttress & RC Anderson; numerous individuals photographed & measured but not preserved, 13-35 mm, Maldives Expedition, N Yonow (see above and Yonow 1994, 1996) and 1986-1999, J Hinterkircher. – Christmas Island: 15 × 8 mm preserved (NHMUK 1899.5.26.13), Flying Fish Cove, leg. Sir J Murray (Andrews).

#### Remarks.

**Figure 24.**
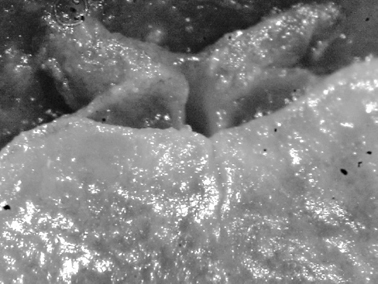
*Phyllidiella striata*, 35 mm (NY-202), view of propodium, head, and oral tentacles.

**Plate 71.**
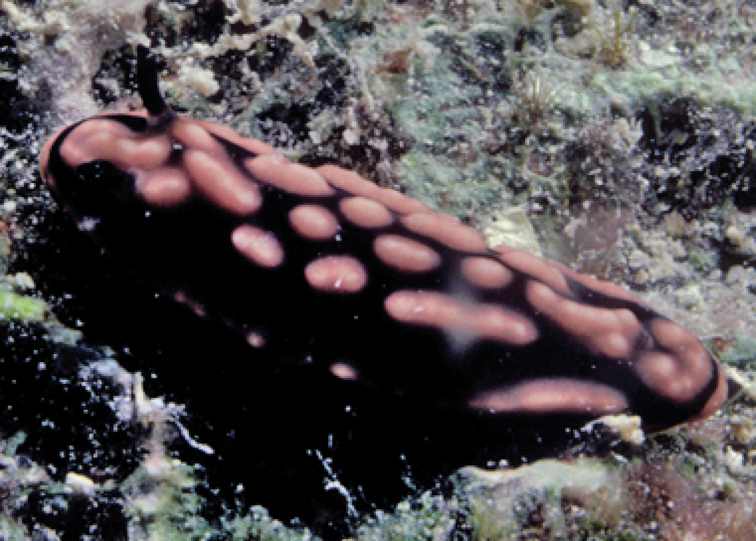
*Phyllidiella striata*, 20 mm, Maldives, photo: N Yonow.

The recognition of Bergh’s species by Yonow et al. (2002) is further supported by these 45 specimens from the Maldives and Christmas Island. The Maldives appears to be a real hot spot for phyllidiid nudibranchs, supporting at least one endemic species (*Phyllidia koehleri*, p. 61) and vast numbers of *Phyllidiella striata* and *Phyllidiella rosans* (see above). *Phyllidiella striata* is most similar to *Phyllidiella rosans*, but the (usually) broken dorsal ridges which are angled behind the rhinophores, which form a circle in front of the rhinophores, and which form a circle around the anus are characteristic (Plate 71). Additionally, the two species differ ventrally, with *Phyllidiella striata* lacking the black pigmentation on the foot and oral tentacles (compare Fig. 22 and Fig. 24). *Phyllidiella striata* ranges from 13-35 mm while *Phyllidiella rosans* is usually larger, measuring up to 52 mm (p. 67) and is much darker ventrally.

### Phyllidiella zeylanica

(Kelaart, 1858)

http://species-id.net/wiki/Phyllidiella_zeylanica

Plates 72 , 73

Phyllidia zeylanica Kelaart, 1858: 120 (Sri Lanka).
Phyllidiella zeylanica . – Yonow 1996: 502, figs. 10A-G (Maldives, Seychelles, SW Thailand, Christmas Island) and references therein; Yonow et al. 2002: 868, fig. 191 (Chagos); Apte 2009: 172, fig. 2x (Laccadive Islands); Matwal and Joshi 2011: 1951, image 1 (western India).

#### Material.

Seychelles: 30 × 15 mm (PK-Y), off Ade’s house, Lilôt, NW Mahé, 10 April 1992, leg. P Kemp; photos of three individuals, Lilôt, NW Mahé, 1988–1989, P Kemp. – Maldives: photos of several individuals, 20–25 mm, March 1997, March 1998, March 1999, J Hinterkircher. – Sri Lanka: photos of one individual, Unawatuna, S of Galle, 26 December 2010, S Kahlbrock.

### Phyllidiopsis gemmata

(Pruvot-Fol, 1957)

http://species-id.net/wiki/Phyllidiopsis_gemmata

Fig. 25 , Plate 74

Phyllidia gemmata Pruvot-Fol, 1957: 121, figs. 50–53, pl. 1 figs. v, vi (no locality, NHMUK).
Phyllidiopsis gemmata . – Brunckhorst 1993: 65, pl. 7H (La Réunion and Thailand).

#### Material.

Maldives: 33 × 15 mm pres. (NY-35), Maayafushi Tila, Ari Atoll, 23 m depth, 14 May 1990, leg. N Yonow (“white with 4 black lines, sole pale grey/white, foot extends beyond margin, has black line dorsally”); photographs of one individual, Maayafushi, South Malé Atoll, March 1997, J Hinterkircher.

#### Description.

Living specimen very white and grey with two longitudinal black lines on each side of centre; each pair meets in front of its respective rhinophore. Outer black lines additionally meet posteriorly beyond anus and extend to mantle margin. Tubercles small and simple, although they may occur in clusters along tuberculate lines. Anus located on a tubercle, and was extended in life. Sole pale grey to white but top of foot with black line.

**Figure 25.**
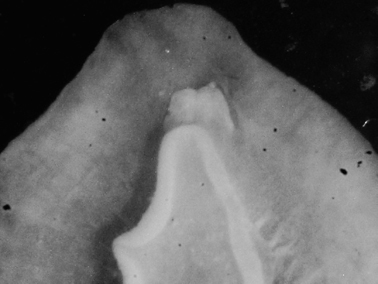
*Phyllidiopsis gemmata*, 33 mm (NY-35), view of propodium, head, and oral tentacles.

In preservative, specimen is distinctive: relaxed, with a very broad but thin mantle skirt. Central tubercles bead-like, arranged in three longitudinal lines; median one several tubercles wide. Skirt has scattered tubercles arranged neither in groups nor in lines. Anterior margin of foot narrow with thickened edge. Head distinctive, forming a “funnel” below broad fused oral tentacles, difficult to see in Fig. 25 (but similar to *Phyllidiella zeylanica*); gills and head white in preserved material.

#### Remarks.

There appear to be very few records of this species in the literature: the species identified as *Phyllidia* cf.* gemmata* by Lin (1983), although listed as such by Brunckhorst (1993), does not appear to be a species of *Phyllidiopsis*: the oral tentacles are the shape of those of species belonging to the genera *Phyllidia* and *Phyllidiella*, separated and long; the shape of the oral tentacles and the three dorsal ridges are more characteristic of *Phyllidia varicosa* or *Phyllidia alyta* although there is apparently no black line on the sole of the foot. Larger specimens of *Phyllidia gemmata* have perpendicular short black lines on the skirt (Pruvot-Fol 1957, Brunckhorst 1993, http://seaslugs.free.fr/nudibranche/a_intro.htm).

### Phyllidiopsis krempfi

Pruvot-Fol, 1957

http://species-id.net/wiki/Phyllidiopsis_krempfi

Fig. 26 , Plate 75

Phyllidiopsis krempfi Pruvot-Fol, 1957: 120, figs. 41–49, pl. 1 figs. 7, 8 (Viet-Nam); Brunckhorst, 1993: 66, fig. 29E, pl. 8A (southwest Thailand); Domínguezet al. 2007: 101, figs. 1E-F, 9 (Papua New Guinea).

#### Material.

Maldives: 35 mm (SH-49), Fulidhoo Reef, Felidhoo Atoll, 21 April 1991, leg. S Harwood. – Sri Lanka: 40 × 20 mm pres., near Pigeon Island, Nilaveli, Trincomalee, 11 March 1995, leg. SG Buttress & RC Anderson.

#### Description.

**Figure 26.**
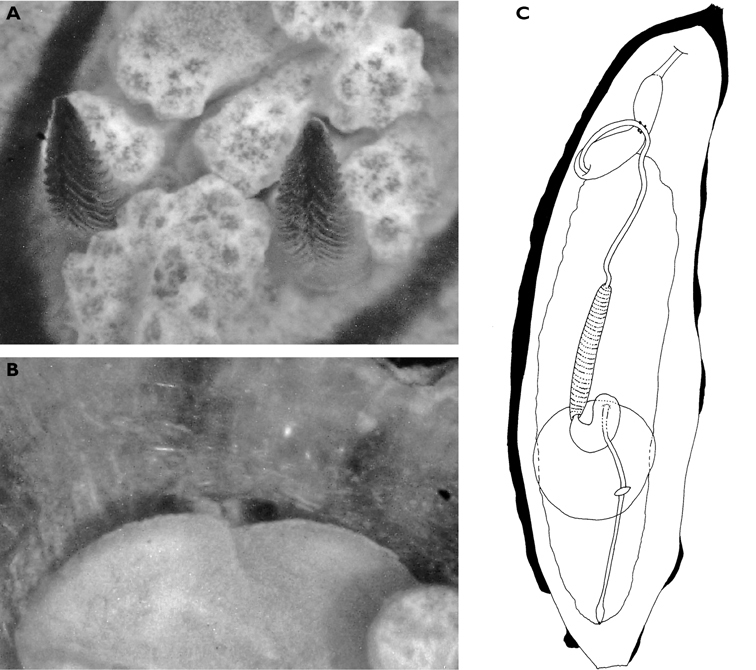
*Phyllidiopsis krempfi*, 40 mm pres. specimen **A** close-up view of rhinophores and rhino-tubercles **B** ventral view of propodium, head, and rounded oral tentacles with black tips **C** digestive anatomy.

Sri Lankan and Maldives specimens bear large compound tubercles centrally, as well as on extended mantle skirt. Pair of black lines meets in V-shape in front of rhinophores (Fig. 26A) and extends to margin; posteriorly, they normally do not meet but bend outwards near the level of the anus and extend to the mantle margin. Traces of black in between central tubercles, as well as spots and streaks on skirt. Anus protrudes in preserved specimens, located on edge of last tubercular cluster. Gills grey, fused oral tentacles with black tips (Fig. 26B). Digestive anatomy typical of genus (Fig. 26C).

#### Distribution.

These records from the Maldives and Sri Lanka are the first records of this species for the western Indian Ocean; it is better known in the western Pacific, where it is larger and more complex: the tubercles become so compound that the longitudinal black lines are almost lost. In the Indian Ocean, it is one of the larger pink species with numerous compound tubercles bearing black lines which always meet in a V-shape in front of the rhinophores.

### Phyllidiopsis shireenae

Brunckhorst, 1990

http://species-id.net/wiki/Phyllidiopsis_shireenae

Fig. 27 , Plate 76

Phyllidiopsis shireenae Brunckhorst, 1990: 557, figs. 1–4 (N Queensland, Papua New Guinea, Solomon Islands); Domínguezet al. 2007: 97, fig. 7 (Papua New Guinea); Yonow 2011: 942 (Indonesia).

#### Material.

Maldives: three specimens 60 × 24 mm (NY-100), 75 × 25 mm (NY-98), and 95 × 33 × 20 mm (NY-99), Maaya Tila, Ari Atoll, 8–30 m depth, 17 May 1990, leg. N Yonow, photos H Voigtmann; 80 mm length (NY dive 42), Maaya Tila, Ari Atoll, 33 m depth, 18 January 1991, leg. N Yonow; individual 40 mm length (photographed but not preserved), Vihafushi Tila, Baa Atoll, 18 m depth, 7 March 1991, S Harwood; individual 30 mm length (photographed but not preserved), Vadhoo Reef, South Malé Atoll, 16 m depth, 3 April 1991, S Harwood; photographs of several individuals, Ari Atoll, approx. 20 m depth, late 1980s, H Voigtmann.

#### Description.

**Figure 27.**
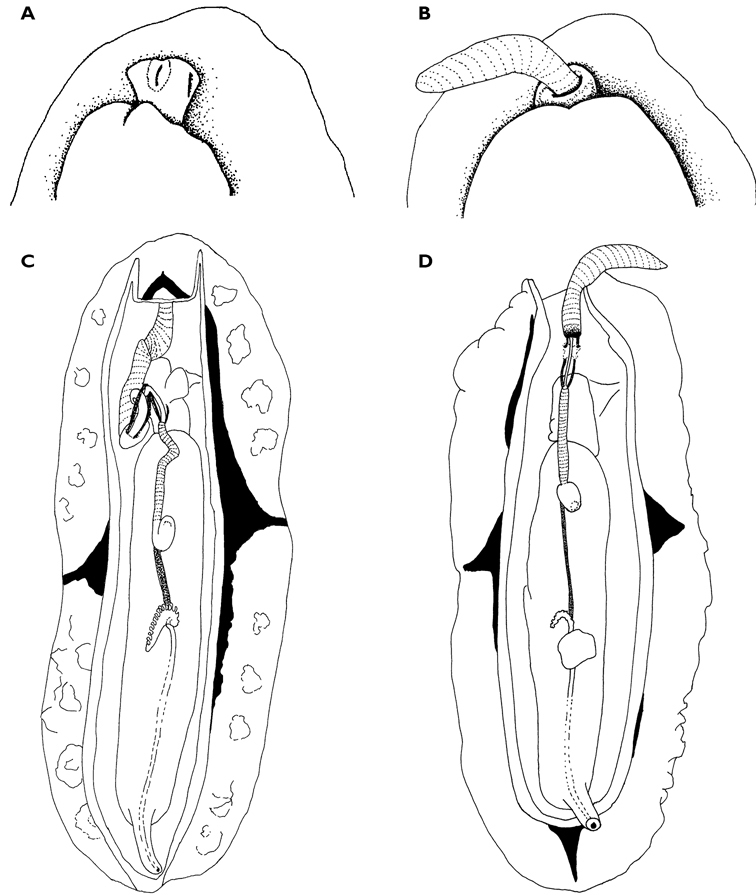
*Phyllidiopsis shireenae* **A** ventral view of 80 mm specimen **B** ventral view of 95 mm specimen with mouthparts extruded **C** digestive anatomy of 75 mm specimen **D** digestive anatomy of 95 mm specimen.

**Plate 76.**
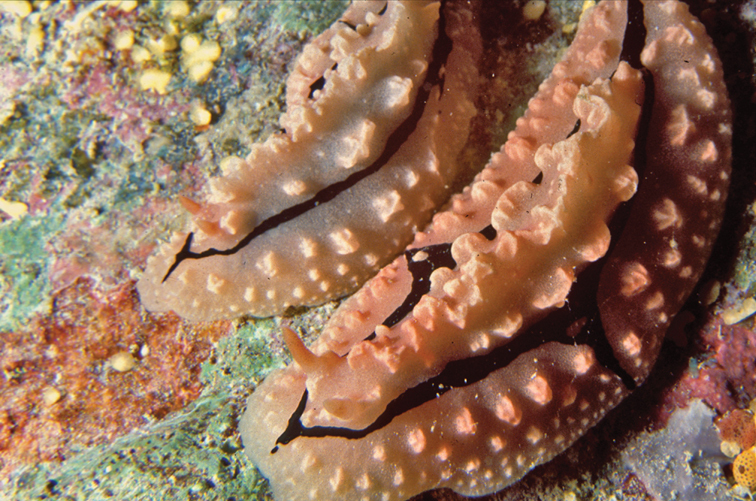
*Phyllidiopsis shireenae*, 60 & 95 mm, Maldives, photo: H Voigtmann.

Body elongate oval, flexible and rubbery, with high profile. Ground colour semi-translucent pink with black line surrounding central region; four smaller perpendicular lines extending to margin in opposing pairs. Central region bears high median crest and lower lateral one either side. Crests composed of irregular granular compound tubercles, opaque in comparison with body. Rhinophores and anal papilla located within black oval. Rhinophores translucent pink, 12 to 19 oblique lamellae; anus conical, located on last tubercle of central crest, visible on larger specimen Plate 76; both extend through raised rims. Outside the black ellipse, a row of large tubercles is followed by a row of smaller ones, also compound and opaque pink-white in life. Metapodium projected beyond the mantle in life. Black band in crevice where hyponotum meets foot behind gills; this black line extends posteriorly along top of metapodium in one specimen. Gills black, genital opening a white swelling on right side. Propodium nearly divided in smaller specimens and notched in the largest. White head and fused oral tentacles, very short with groove on outer sides (figs. 27A).

Internally, three specimens from the Maldives proved to be identical (the fourth was badly preserved). One specimen (95 mm) everted its mouthparts completely during preservation (Fig. 27B, D). The mouth leads to a short oral tube which, in one specimen, is narrow and contracted like a concertina (Fig. 27C). Where it thickens into the pharyngeal bulb, there is a band of pigment, incomplete dorsally. The pharyngeal bulb is extensible, as evidenced by the specimen illustrated in Fig. 27D. A sharp demarcation exists where the pharynx joins the bulb; the bulb turns anteriorly and loops posteriorly, passing through the nerve ring. The posteriorly-directed portion is of equal length to the anteriorly-directed segment, and inserts into the oesophagus at a constriction. The oesophagus is a thicker banded tube leading into a sac-like structure with which it is continuous (the muscular oesophageal segment), and opens into the digestive gland. The pathway from the oesophagus to the digestive gland follows a Z-shaped route, located in the anterior quarter of the digestive gland. Almost in a continuous line with the oesophagus, a long muscle attaches and runs under the intestine and pericardium to emerge on the other side. The intestine then runs along the midline to the anus.

#### Distribution.

These records greatly extend the range of the species from the tropical western Pacific to the central Indian Ocean. It was previously described from eastern Australia, the Solomon Islands (Brunckhorst 1990), Papua New Guinea (Domínguez et al. 2007), and Ambon, Indonesia (Yonow 2011). The internal anatomy of the Indian Ocean specimens differs somewhat from Brunckhorst’s material, which is presented as a generalised figure, but it is unlikely that more than one species are involved: the detailed description and illustrations of the Papua New Guinea material are more comparable (Domínguezet al. 2007).

### Phyllidiopsis sphingis

Brunckhorst, 1993

http://species-id.net/wiki/Phyllidiopsis_sphingis

**Figure 28.**
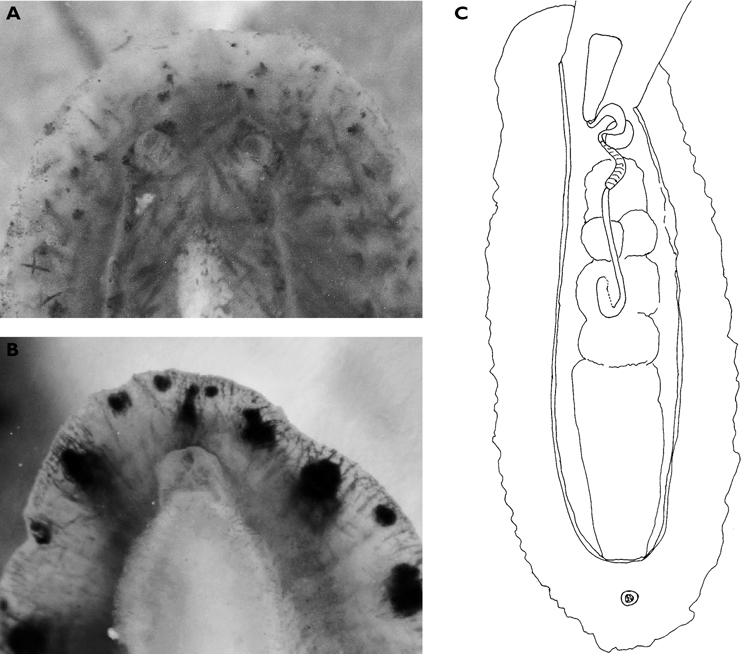
*Phyllidiopsis sphingis* **A** dorsal view of rhinophores and spicule alignment of 19 mm specimen **B** ventral view of 17 mm specimen showing the large fused oral tentacles **C** digestive anatomy of 19 mm specimen.

**Plate 77.**
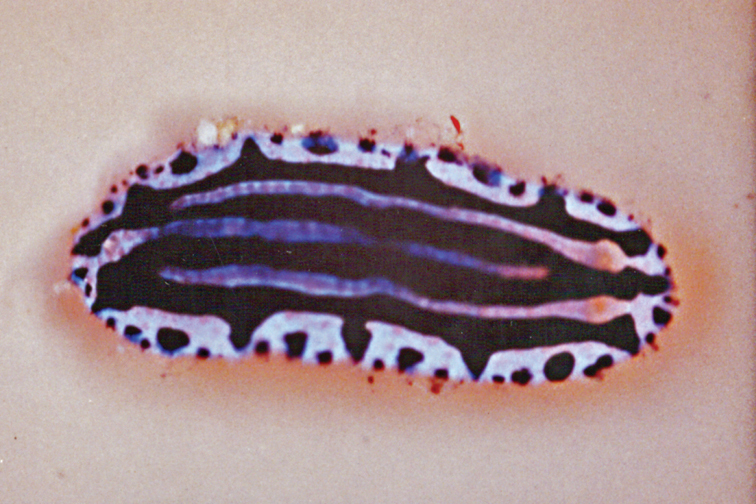
*Phyllidiopsis sphingis*, 19 mm, Maldives, photo: RC Anderson.

**Plate 78.**
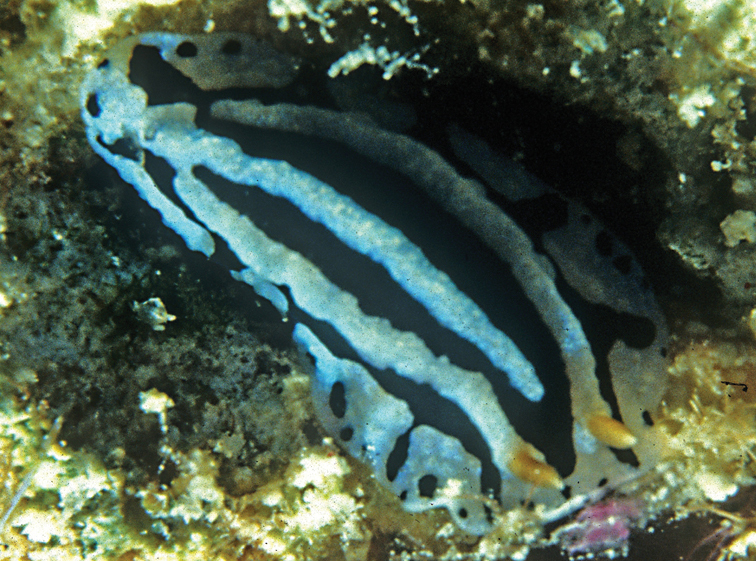
*Phyllidiopsis sphingis*, 17 mm, Maldives, photo: S Harwood.

Fig. 28 , Plates 77 , 78

Phyllidiopsis sphingis Brunckhorst, 1993: 72, pl. 8H (Hawaii, Guam, Papua New Guinea).

#### Material.

Maldives: 17 mm (SH-7, “yellow rhinophores”), Fehigili, Lhaviyani Atoll, 30 m depth, 05 March 1991, leg. S Harwood; 19 × 10 mm (MDV/AB/96/18, “pale blue, no tubercles, rhinophores cream/pale yellow”), Yacht Tila, near Medufinolhu, South Malé Atoll, 10 m depth, 09 May 1996, leg. RC Anderson & SG Buttress.

#### Description.

Both specimens well relaxed, virtually identical to each other with black linear pattern composed of four lines: median pair merges between rhinophores and extends to margin anteriorly, posteriorly they remain separate to anus or meet outer lines at level of anus. Outer lines straight medially but scalloped laterally in extensions to margin; scallops not symmetrical on each side, with five on left and six on right on both specimens. Collectors’ sketches identical to illustration in Brunckhorst, 1993; although only one collector comments on blue colour, both comment on ochre-yellow rhinophores, visible in photographs of both specimens. Anal papilla ochre, very near posterior margin. Photograph of 19 mm specimen (Plate 77) shows central area between black lines to have been blue, outer scalloped areas were granular white with blue spots along margin. Faint orange tinge behind rhinophores (and generally) is a photographic artefact.

The specimens, both preserved in formaldehyde, are very different: one is perfectly marked as if alive, with extended rhinophores and black markings (17 mm, Plate 78). The second appears ‘bleached’ and translucent, to the point where the black spicules aligned in the central region above the yellowish gut are perfectly clear (Fig. 28A). Ventrally, the head is typical of species of *Phyllidiopsis*, a large fused unit with the grooves of the tentacles just visible in the pigmented specimen; spicules visible along foot margin and on hyponotum (Fig. 28B). The hyponotum, foot sole, and gills are cream coloured.

Internally, the digestive anatomy is typical of the genus: the solid pharyngeal bulb has a flat end anteriorly and tapers posteriorly. The tubular pharynx is narrow and turns on itself to pass through the nerve ring before joining the muscular oesophagus, which is banded and brown-black. There is a long narrow cream oesophagus which enters the digestive gland at approximately the one third mark (fig. 28C). The anal papilla is slightly swollen.

#### Distribution. 

This is the first record of *Phyllidiopsis sphingis* for the Indian Ocean. Both specimens are small, but within the range reported for West Pacific specimens (4–23 mm). They differ from the Pacific records which have blue pigment only on the mantle skirt. The species in the Maldives is similar to *Phyllidia koehleri* in its small size, a ridged dorsum rather than a tuberculate one, and with the anus located very far posteriorly.

### Phyllidiopsis xishaensis

(Lin, 1983)

http://species-id.net/wiki/Phyllidiopsis_xishaensis

Fig. 29 , Plate 79

Phyllidia xishaensis Lin, 1983: 153, pl. 1 fig. 2 (China); Yonow et al. 2002: 870, fig. 19c (Chagos).
Phyllidiopsis striata . – Brunckhorst 1993: 69, fig. 29H, pl. 8D, E (W Australia, SW Thailand + W Pacific); Apte 2009: 172, fig. 3b (Laccadive Islands) (non *Phyllidiopsis striata* Bergh).

#### Material.

Maldives: 21 × 8 mm (NY-34, “translucent white, tiny oral tentacles ochre like rhinophores”), Maaya Tila, Ari Atoll, 15 m depth, 14 May 1990, leg. N Yonow; 15 mm (SH-12, “yellow rhinophores make him different”), Boamandhifurihurai, Lhaviyani Atoll, 6 m depth, 05 March 1991, leg. S Harwood; 12 × 5 mm pres. (“with dots around edge”), Hans Place, south end of North Malé Atoll, 9 m depth, 11 October 1994, leg. SG Buttress & RC Anderson; photographs of five individuals, one measuring 15 mm, March 1997, March 1998, March 1999, J Hinterkircher. – Mauritius, La Réunion, and Mayotte: numerous individuals photographed, 6–19 mm alive (http://seaslugs.free.fr/nudibranche/a_intro.htm). – Seychelles: photos of two individuals, Lilôt, NW Mahé, 1988–1989, P Kemp.

#### Remarks.

**Figure 29.**
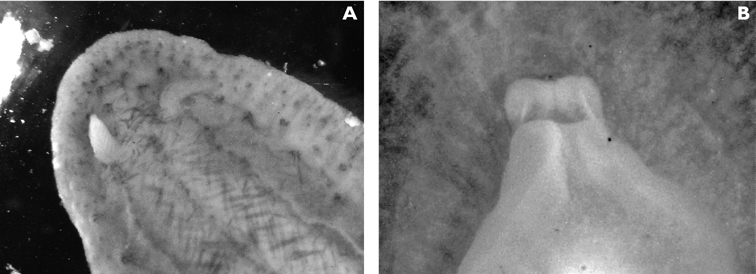
*Phyllidiopsis xishaensis* **A** dorsal view of anterior end of 15 mm specimen **B** ventral view of propodium of 21 mm specimen.

The resurrection of this species and the rationale behind this were described in Yonow et al. (2002). These specimens from the western Indian Ocean clearly conform to the original (and subsequent) illustrations of the species (erroneously identified as *Phyllidiopsis striata* in the literature and on many websites) and further illustrations are provided here of the anterior dorsum (Fig. 29A) and head/oral tentacles (Fig. 29B). It has an isolated distribution in the western Indian Ocean, from the Laccadive and Maldive islands south to Chagos and west to Mauritius, La Réunion, and Mayotte; there are no specimen records from East or South Africa.

## Discussion

Preliminary evaluation of this particular assemblage of species shows no real patterns at present. Without doubt there are a number of species which seem to be limited to the western Indian Ocean, especially within the chromodorids and phyllidiids. At present, only the species covered in this paper are tabulated for locality, with a total for each location (Table 1). La Réunion and Mauritius are ranked as the most diverse, with a total of 48 species, closely followed by the Maldives with 45 species (except Kenya, see below). A number of species appear to be limited to the Maldives, despite similar collection methodologies in the other regions studied: *Nembrotha guttata* and *Notodoris gardineri* form *nigerrima* only occur here, as does *Phyllidia koehleri*. Of the ten new records for the Indian Ocean, seven are recorded from the Maldives, four of which are recorded exclusively from that locality. Three somewhat rare West Pacific phyllidiids are also recorded only from the Maldives, or also at one other locality: *Phyllidia exquisita*, *Phyllidiella rudmani*, and *Phyllidiopsis gemmata*. It is a little surprising that Mauritius, with its much longer history of research, does not rank as highly as the Maldives for this clutch of species, despite the establishment of the Mauritius/La Réunion website (however, see discussion on Kenya below). Admittedly there are many species which have not been included, but the current comparison of “like-for-like” is revealing some interesting observations. The area with the most species in common with the Maldives is Mauritius/La Réunion, with 25 species occurring in both.  This contrasts with, for example, South Africa which has been under study for many years: of the 29 species recorded here, the most similar is Tanzania with 18 species in common followed by Mauritius and La Réunion with 18 species, while single studies, such as Chagos and the Laccadives for example, provide relatively high diversities of 23 and 21 species respectively.

The highest number of species recorded for the countries listed in Table 1 was for Kenya; this is an ‘experimental’ result produced by using the locations tab on the NudiPixel website, where Kenya has the fourth highest number of species, followed by India and South Africa in the Indian Ocean. Photographs from Kenya on NudiPixel of the species covered in this paper were carefully examined and, if verified, they were inserted in my table as NP; these records have a very high chance of occurring in Kenya and for this experiment were deemed acceptable. This increased the species records for Kenya from 17 to 49, and simply shows only that the numbers produced by scientific papers alone are not indicative of the real biodiversity of an area as the variables (collecting method, season, duration, experience, etc.) are numerous, and many more studies such as this one are required. As collections in these areas increase with environmental monitoring and biodiversity projects, and more work is written up (Yogesh Kumar et al. 2011 increased the total for India from 16 to 24 while this paper was in press), more meaningful conclusions may be drawn in the future. The only real conclusion is that much remains unknown and additional research is badly needed.

## Supplementary Material

XML Treatment for Haminoea cymbalum

XML Treatment for Chelidonura electra

XML Treatment for Chelidonura hirundinina

XML Treatment for Chelidonura punctata

XML Treatment for Chelidonura sandrana

XML Treatment for Chelidonura varians

XML Treatment for Philinopsis speciosa

XML Treatment for Aplysia parvula

XML Treatment for Dolabella auricularia

XML Treatment for Dolabrifera dolabrifera

XML Treatment for Notarchus indicus

XML Treatment for Stylocheilus longicauda

XML Treatment for Cyerce bourbonica

XML Treatment for Elysia nigropunctata

XML Treatment for Plakobranchus ocellatus

XML Treatment for Thuridilla gracilis

XML Treatment for Thuridilla vataae

XML Treatment for Nembrotha guttata

XML Treatment for Roboastra gracilis

XML Treatment for Tambja amakusana

XML Treatment for Tambja morosa

XML Treatment for Notodoris gardineri nigerrima

XML Treatment for Notodoris minor

XML Treatment for Hexabranchus sanguineus

XML Treatment for Carminodoris grandiflora

XML Treatment for Halgerda formosa

XML Treatment for Halgerda punctata

XML Treatment for Halgerda tessellata

XML Treatment for Cadlinella ornatissima

XML Treatment for Ceratosoma miamirana

XML Treatment for Chromodoris africana

XML Treatment for Chromodoris annulata

XML Treatment for Chromodoris boucheti

XML Treatment for Chromodoris cavae

XML Treatment for Chromodoris conchyliata

XML Treatment for Chromodoris decora

XML Treatment for Chromodoris geometrica

XML Treatment for Chromodoris gleniei

XML Treatment for Chromodoris hamiltoni

XML Treatment for Chromodoris quadricolor

XML Treatment for Chromodoris tennentana

XML Treatment for Glossodoris cincta

XML Treatment for Glossodoris pallida

XML Treatment for Glossodoris undaurum

XML Treatment for Hypselodoris maculosa

XML Treatment for Risbecia bullockii

XML Treatment for Risbecia pulchella

XML Treatment for Thorunna horologia

XML Treatment for Dendrodoris fumata

XML Treatment for Dendrodoris nigra

XML Treatment for Doriopsilla nigrocera

XML Treatment for Phyllidia (Fryeria) marindica

XML Treatment for Phyllidia (Fryeria) picta

XML Treatment for Phyllidia alyta

XML Treatment for Phyllidia coelestis

XML Treatment for Phyllidia exquisita

XML Treatment for Phyllidia koehleri

XML Treatment for Phyllidia multituberculata

XML Treatment for Phyllidia varicosa

XML Treatment for Phyllidiella meandrina

XML Treatment for Phyllidiella pustulosa

XML Treatment for Phyllidiella rosans

XML Treatment for Phyllidiella rudmani

XML Treatment for Phyllidiella striata

XML Treatment for Phyllidiella zeylanica

XML Treatment for Phyllidiopsis gemmata

XML Treatment for Phyllidiopsis krempfi

XML Treatment for Phyllidiopsis shireenae

XML Treatment for Phyllidiopsis sphingis

XML Treatment for Phyllidiopsis xishaensis

## Figures and Tables

**Plate 1.**
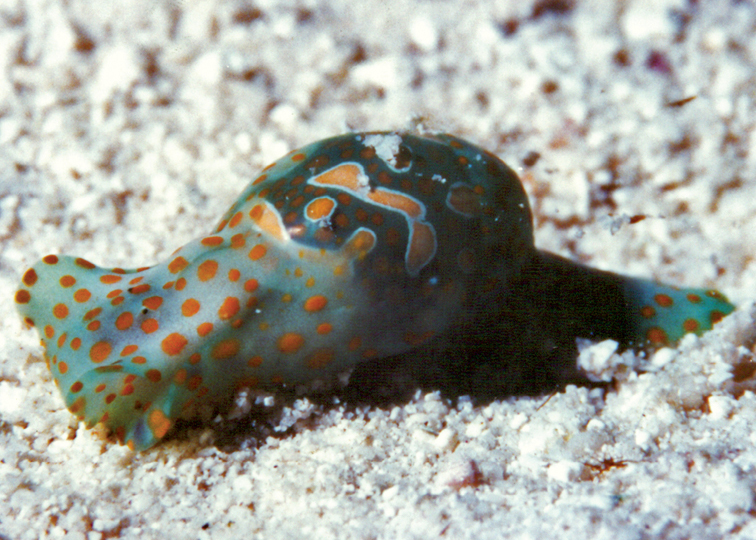
*Haminoea cf. cymbalum*, 20 mm, Maldives, photo: RC Anderson.

**Plate 2.**
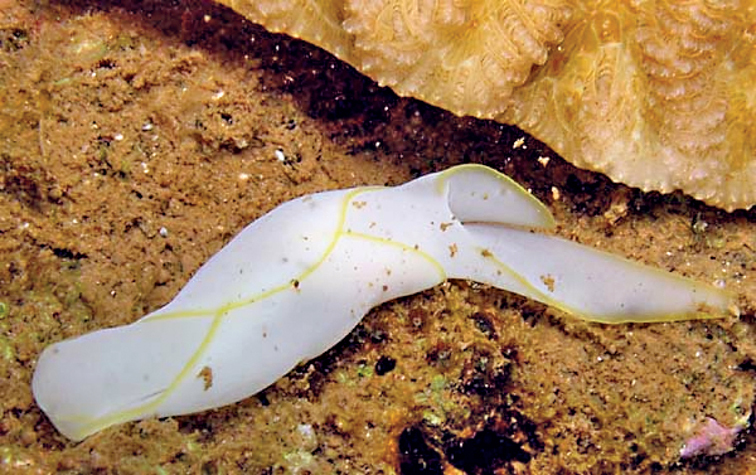
*Chelidonura electra*, 20–25 mm, Mayotte, photo: P Bidgrain.

**Plate 3.**
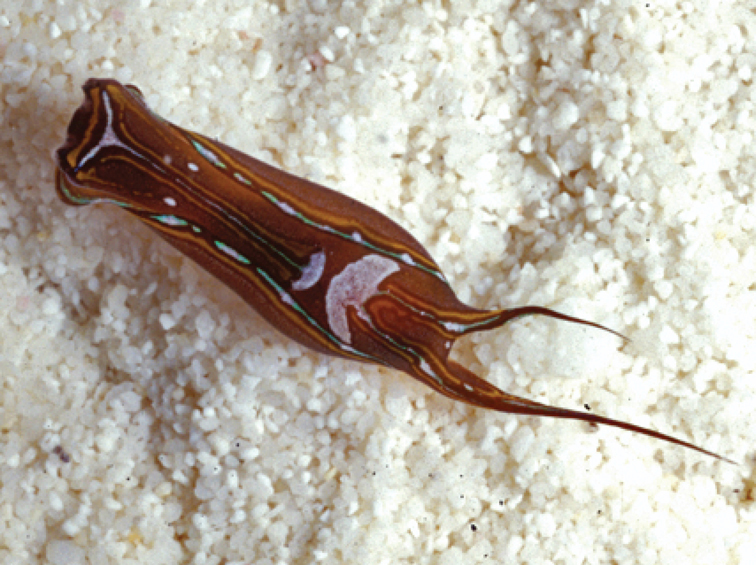
*Chelidonura hirundinina*, 5–6 mm pres., Maldives, photo: J Hinterkircher.

**Plate 4.**
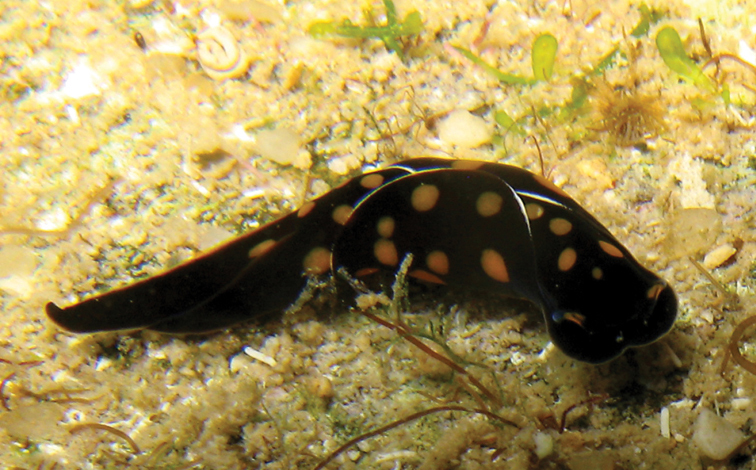
*Chelidonura punctata*, 18–20 mm, La Réunion, photo: P Bidgrain.

**Plate 8.**
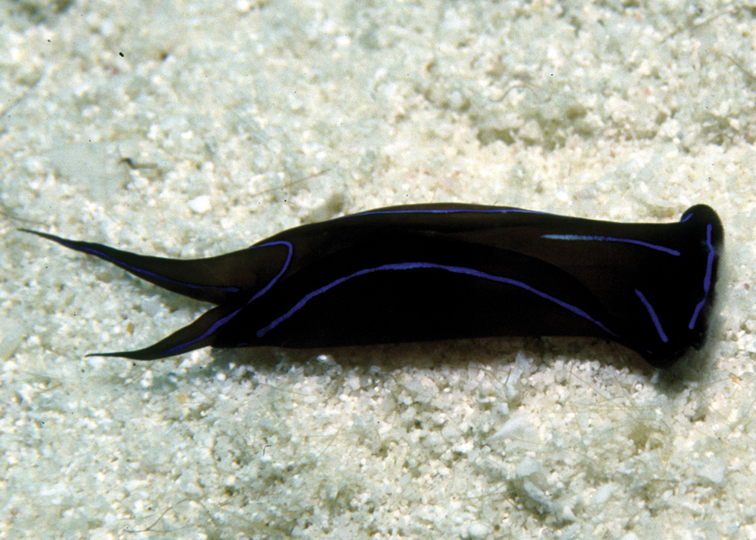
*Chelidonura varians*, 30 mm, Maldives, photo: RC Anderson.

**Plate 9.**
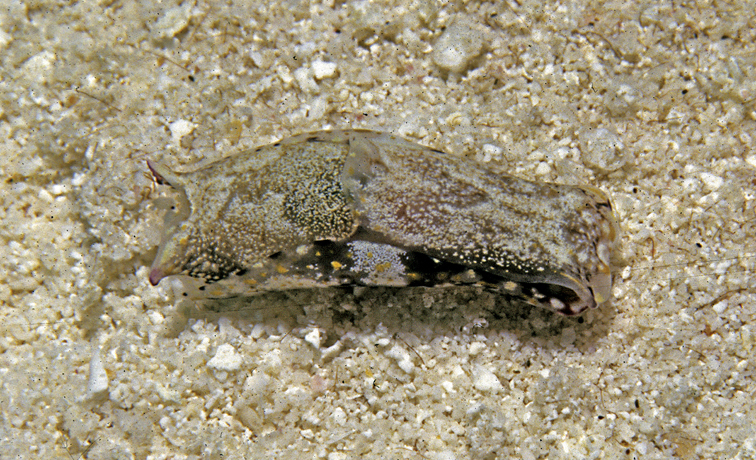
*Philinopsis speciosa*, 18 mm pres., Maldives, photo: RC Anderson.

**Plate 12.**
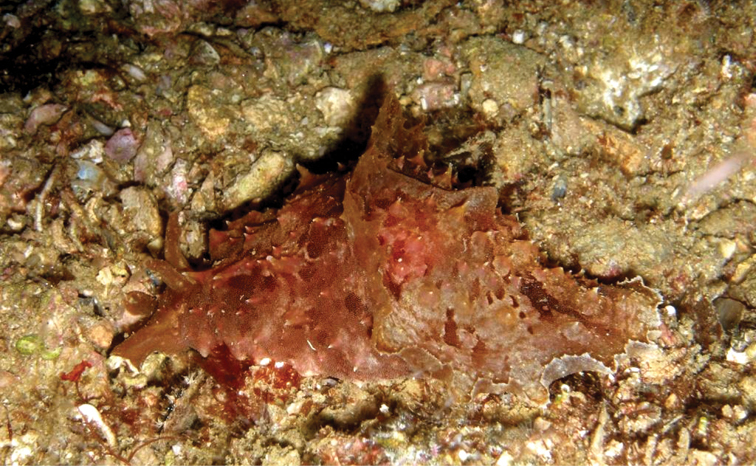
*Dolabella auricularia*, Oman, photo: S Kahlbrock (no spcm).

**Plate 13.**
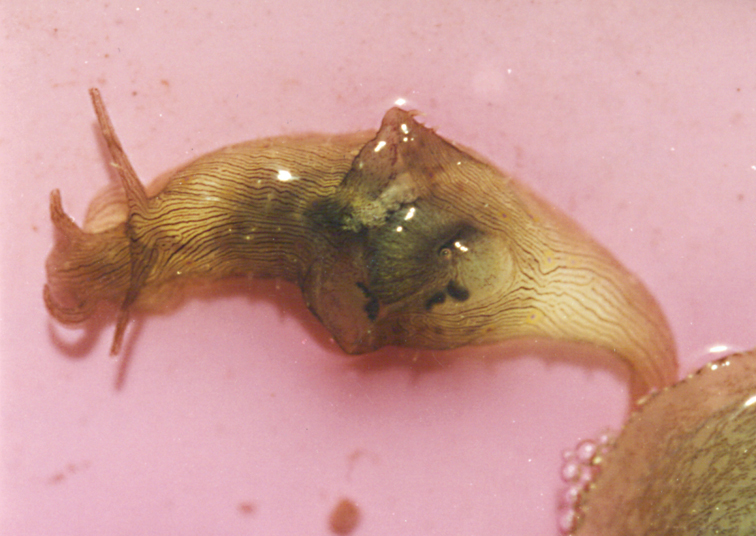
*Stylocheilus longicauda* form *striata*, 10–50 mm pres., Zanzibar, photo: MD Richmond.

**Plate 16.**
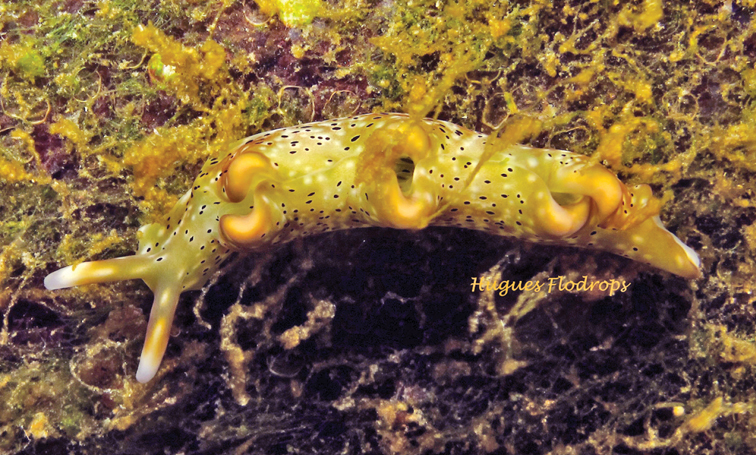
*Elysia cf. nigropunctata*, 35 mm, La Réunion, photo: H Flodrops (no spcm).

**Plate 17.**
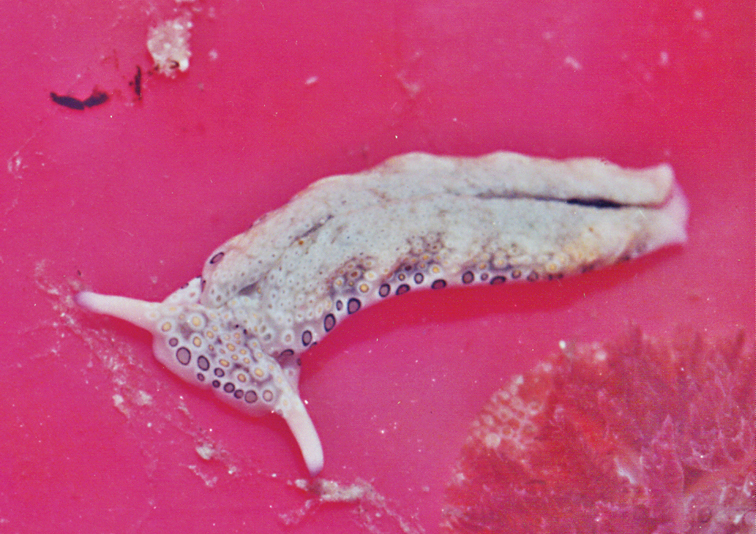
*Plakobranchus ocellatus*, 14 mm pres., Zanzibar, photo: MD Richmond.

**Plate 20.**
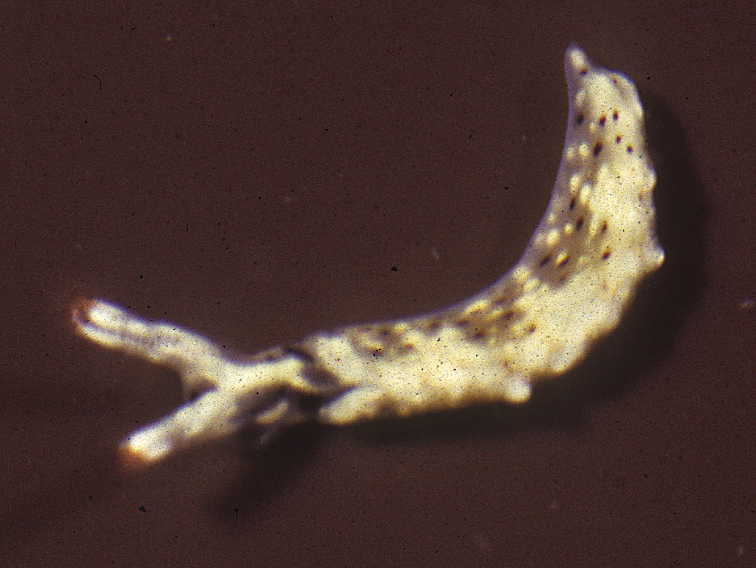
*Thuridilla vataae*, 10 mm, Maldives, photo: RC Anderson.

**Plate 22.**
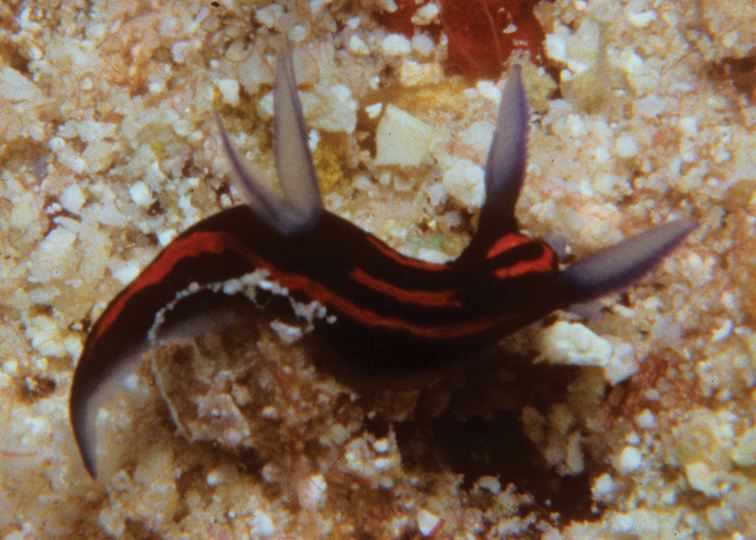
*Roboastra gracilis*, 20 mm, Maldives, photo: J Hinterkircher (no spcm).

**Plate 24.**
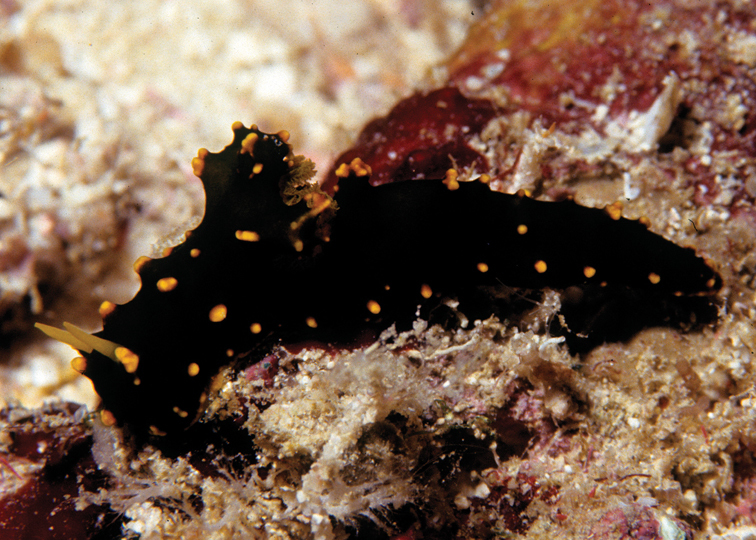
*Notodoris gardineri* form *nigerrima*, 40 mm, Maldives, photo: RC Anderson.

**Plate 25.**
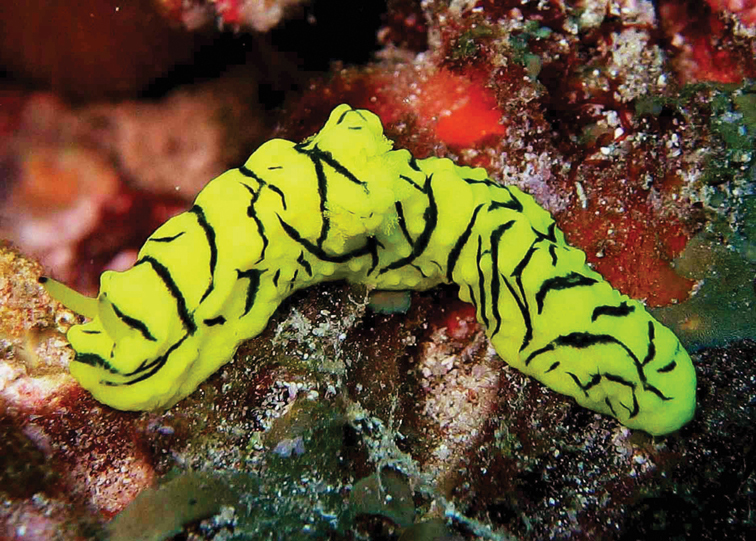
*Notodoris minor*, Pemba Island, photo: A de Villiers (no spcm).

**Plate 28.**
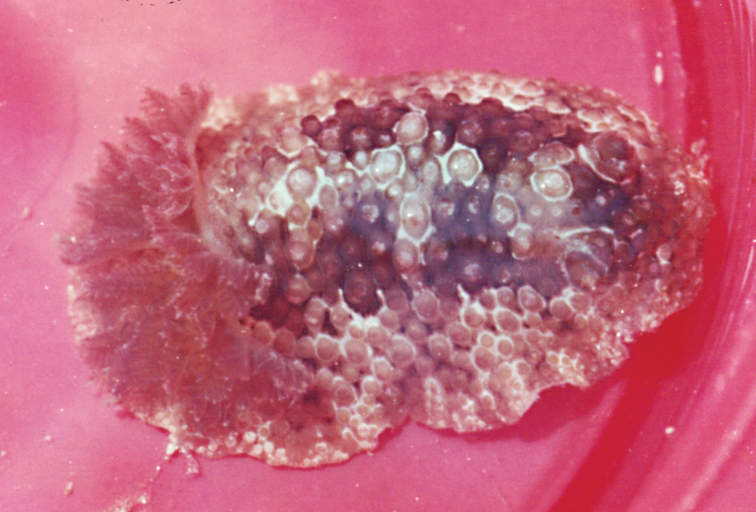
*Carminodoris grandiflora*, 30 mm pres., Zanzibar, photo: MD Richmond.

**Plate 29.**
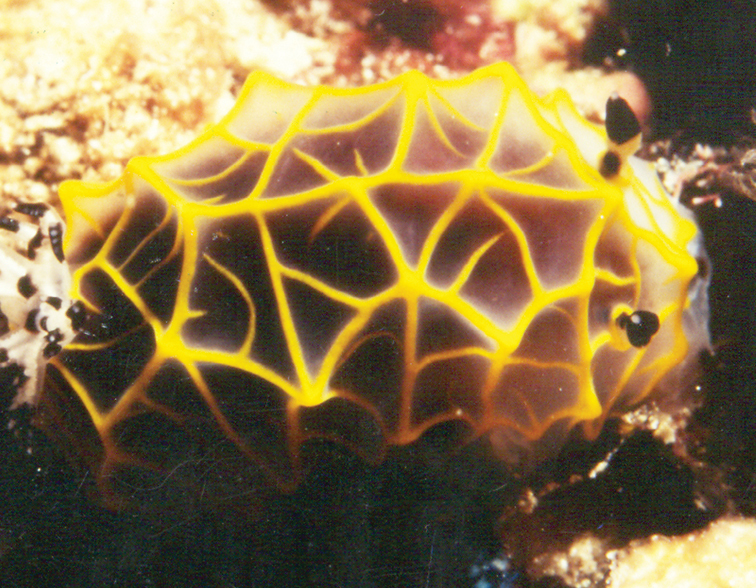
*Halgerda formosa*, Kenya, photo: MD Richmond (no spcm).

**Plate 31.**
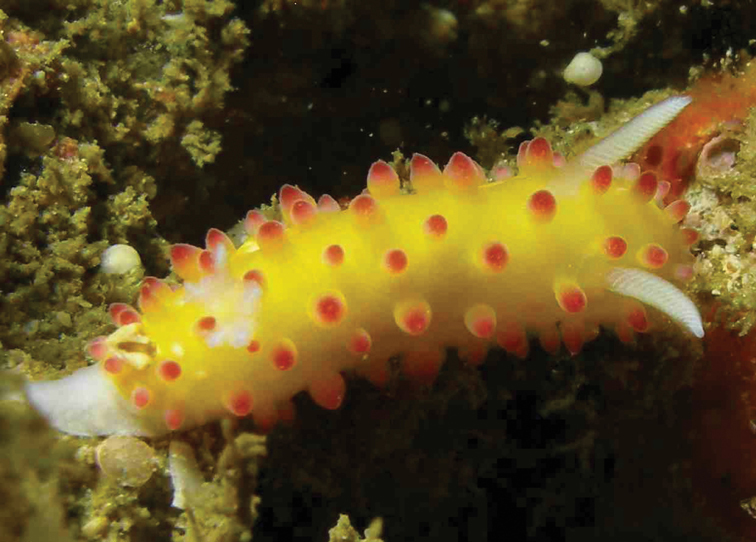
*Cadlinella ornatissima*, 13 mm pres., Sri Lanka, photo: S Kahlbrock.

**Plate 32.**
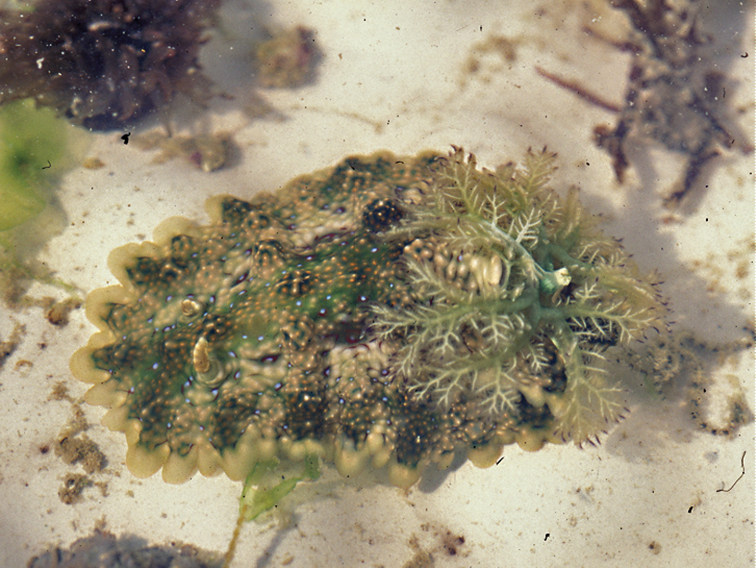
*Ceratosoma miamirana*, 36 mm pres., Zanzibar, photo: MD Richmond.

**Plate 33.**
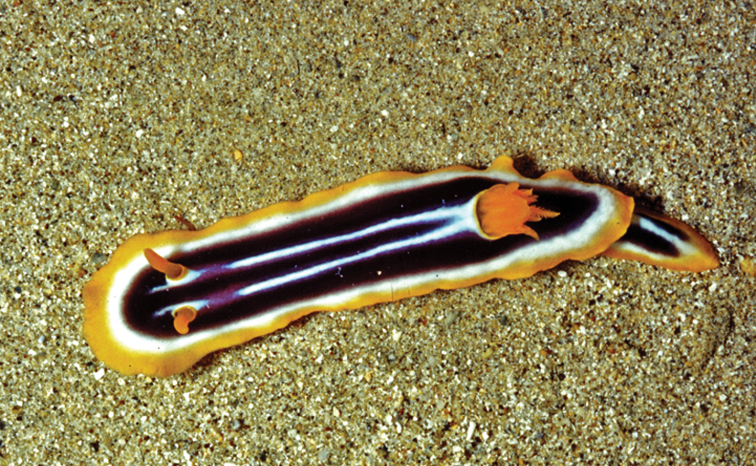
*Chromodoris africana*, Madagascar, photo: J Hinterkircher (no spcm).

**Plate 36.**
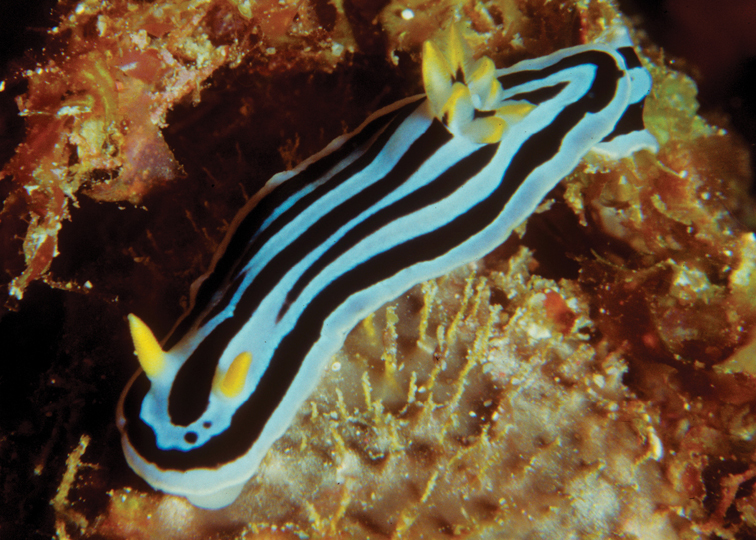
*Chromodoris boucheti*, Maldives, photo: J Hinterkircher (no spcm).

**Plate 44.**
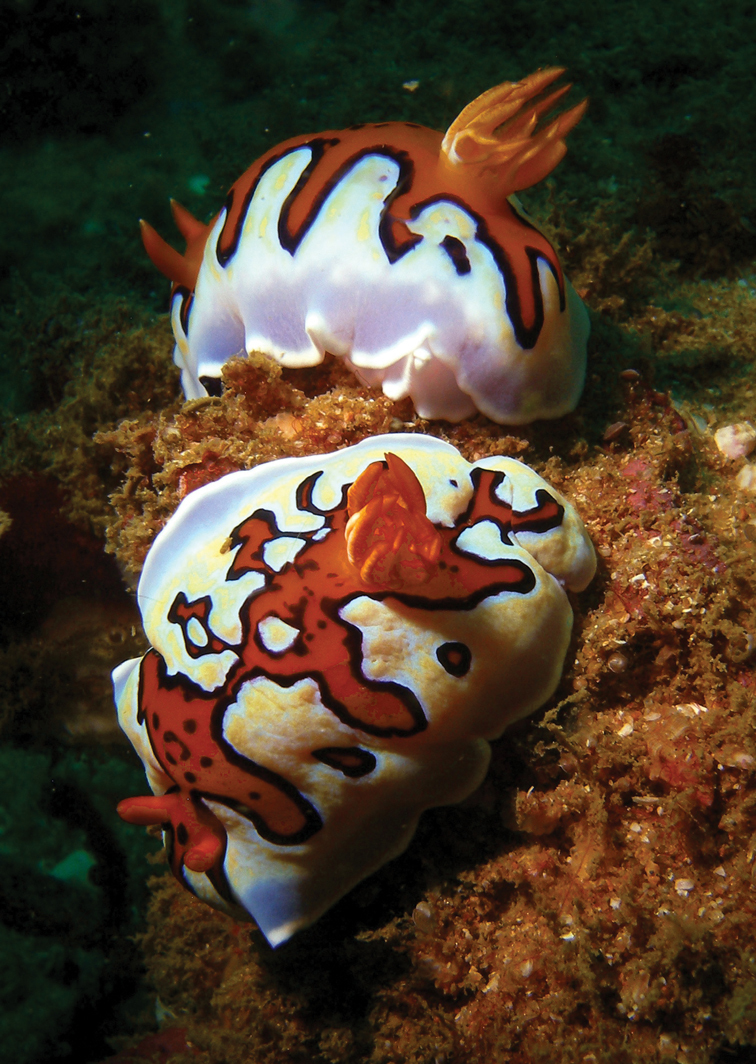
*Chromodoris gleniei*, Sri Lanka, photo: S Kahlbrock (no spcm).

**Plate 45.**
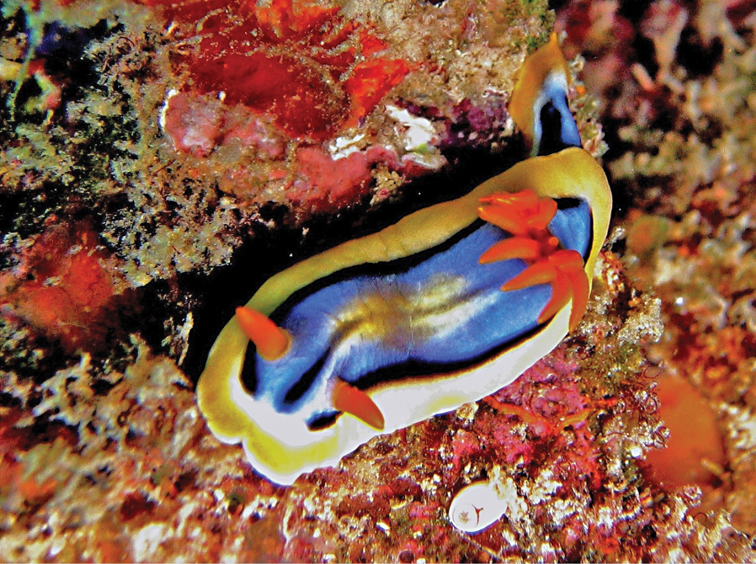
*Chromodoris hamiltoni*, Tanzania, photo: A de Villiers (no spcm).

**Plate 46.**
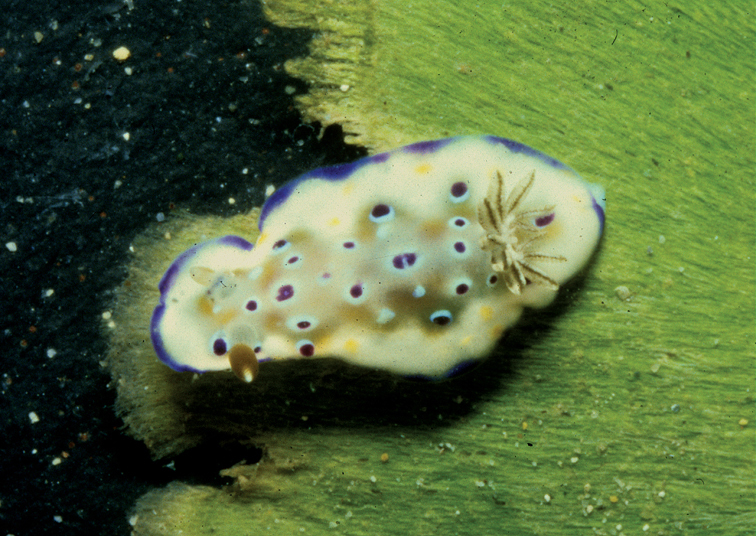
*Chromodoris tennentana*, 10 mm, Madagascar, photo: J Hinterkircher (no spcm).

**Plate 47.**
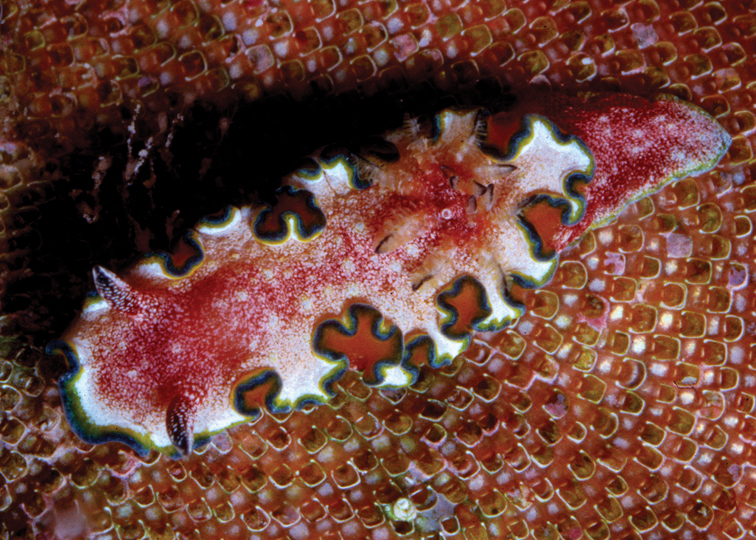
*Glossodoris cincta*, 15 mm, Maldives, on the bryozoan Steginoporella, photo: J Hinterkircher.

**Plate 49.**
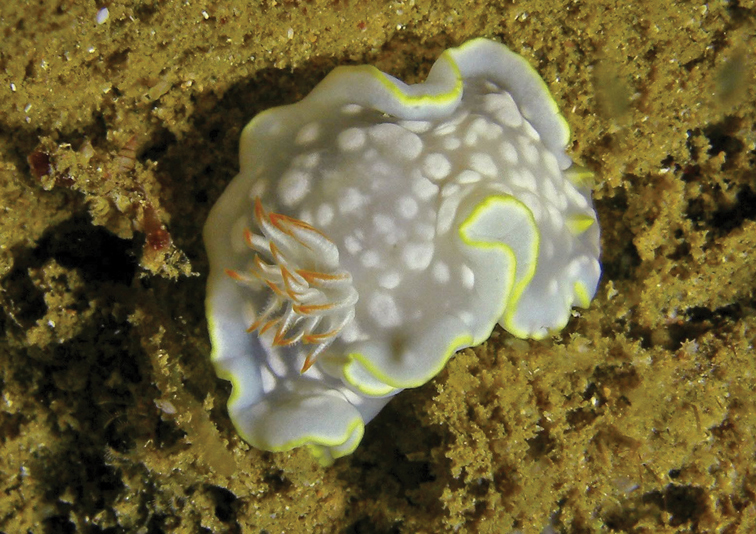
*Glossodoris undaurum*, 13 mm pres., Sri Lanka, S Kahlbrock.

**Plate 50.**
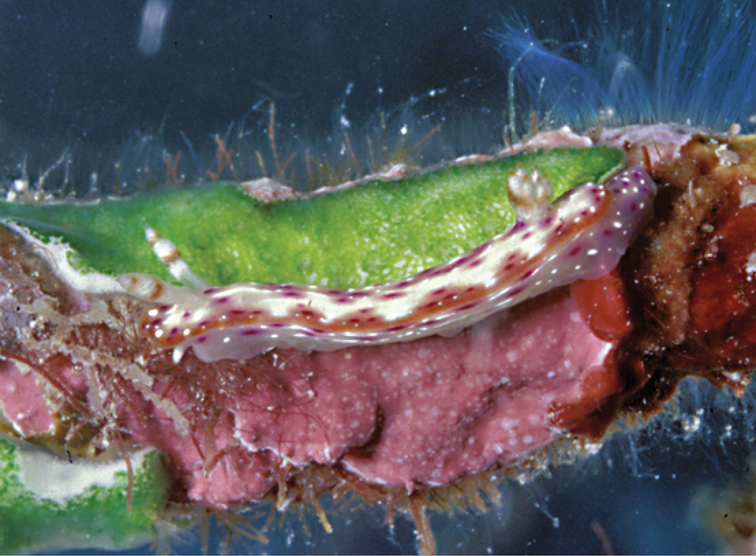
*Hypselodoris maculosa*, 15 mm, Maldives, photo: J Hinterkircher.

**Plate 54.**
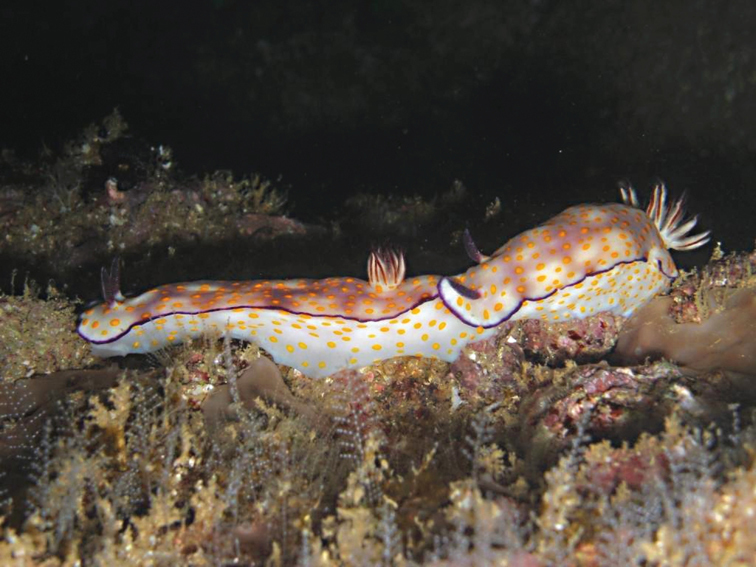
*Risbecia pulchella*, Oman, photo: S Kahlbrock (no spcm).

**Plate 55.**
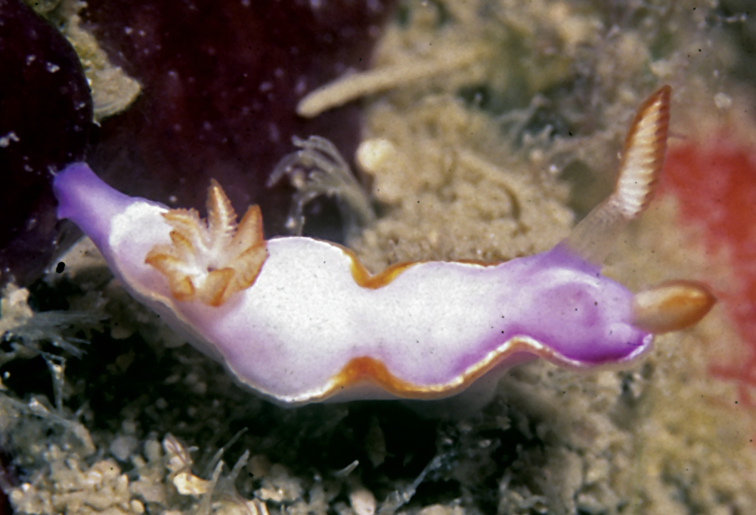
*Thorunna cf. horologia*, 5 mm pres., Sri Lanka, RC Anderson.

**Plate 57.**
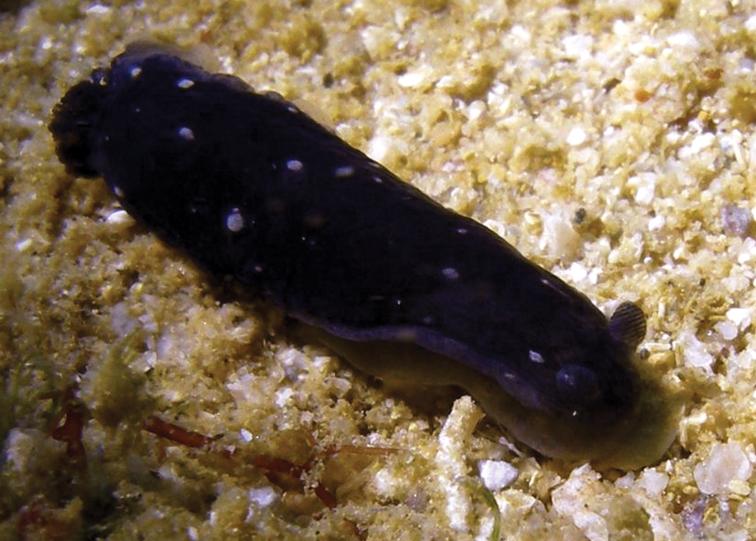
*Dendrodoris nigra*, Oman, photo: S Kahlbrock (no spcm).

**Plate 60.**
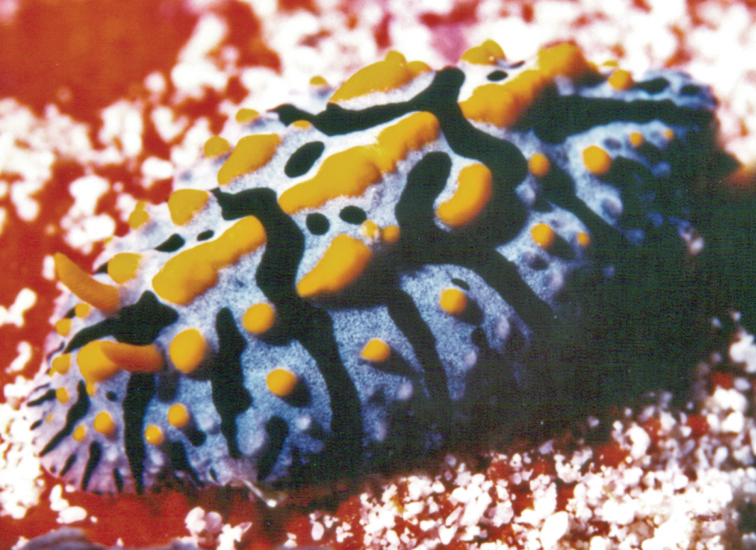
*Fryeria marindica* with Pacific pattern, 35 mm, Maldives, photo: RC Anderson.

**Plate 62.**
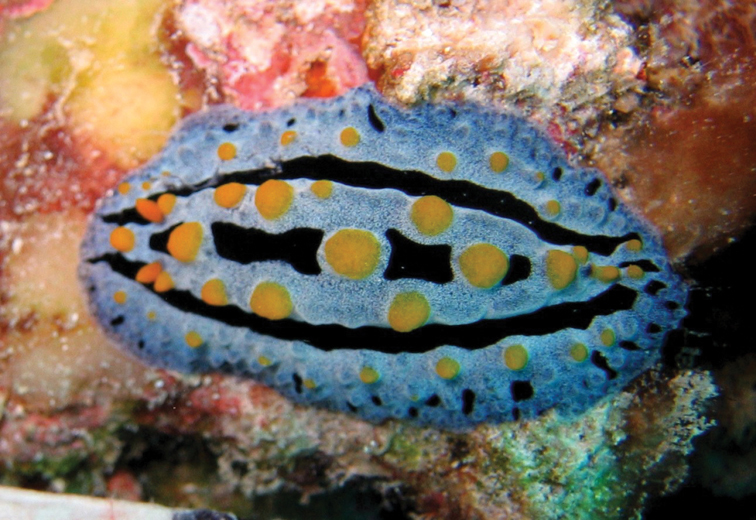
*Phyllidia coelestis*, Tanzania, photo: A de Villiers (no spcm).

**Plate 67.**
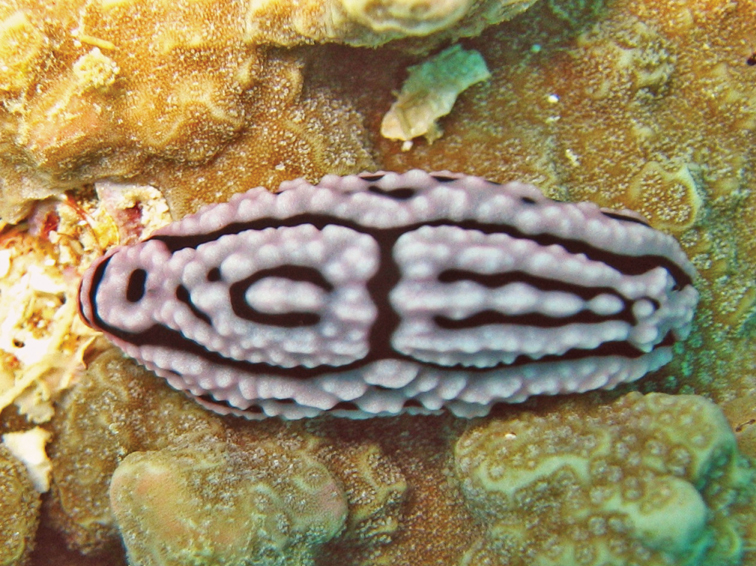
*Phyllidiella meandrina*, Tanzania, photo: A de Villiers (no spcm).

**Plate 68.**
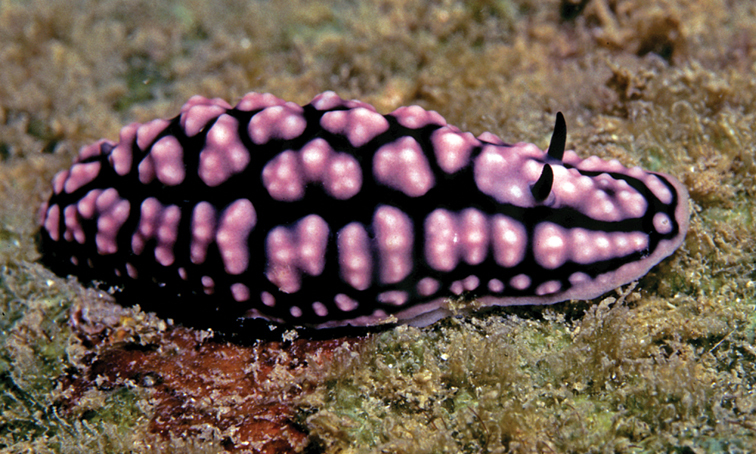
*Phyllidiella pustulosa*, Sri Lanka, photo: RC Anderson (no spcm).

**Plate 69.**
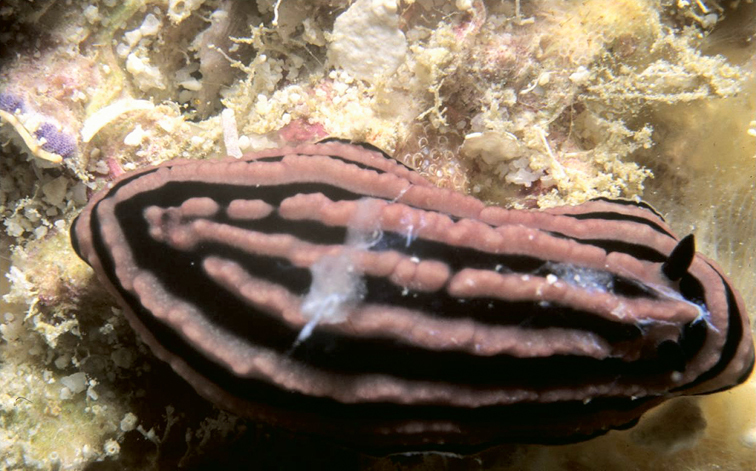
*Phyllidiella rosans*, 30 mm, Maldives, photo: N Yonow (note defensive exudates).

**Plate 70.**
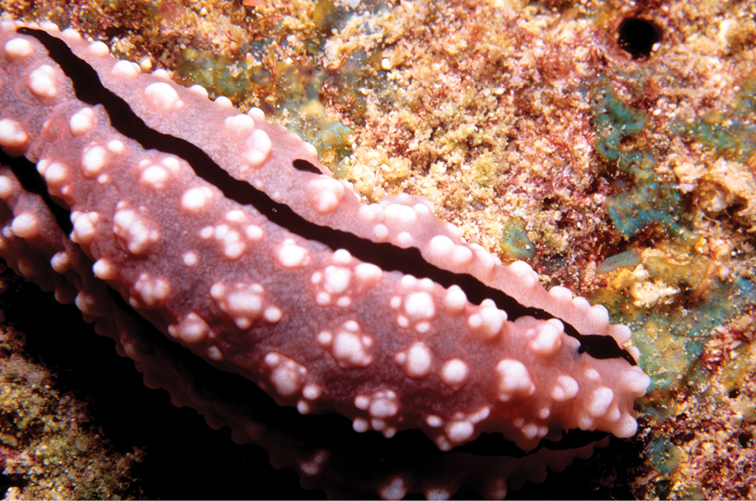
*Phyllidiella rudmani*, 35 mm, Maldives, photo: N Yonow (spcm in Yonow 1996).

**Plate 72.**
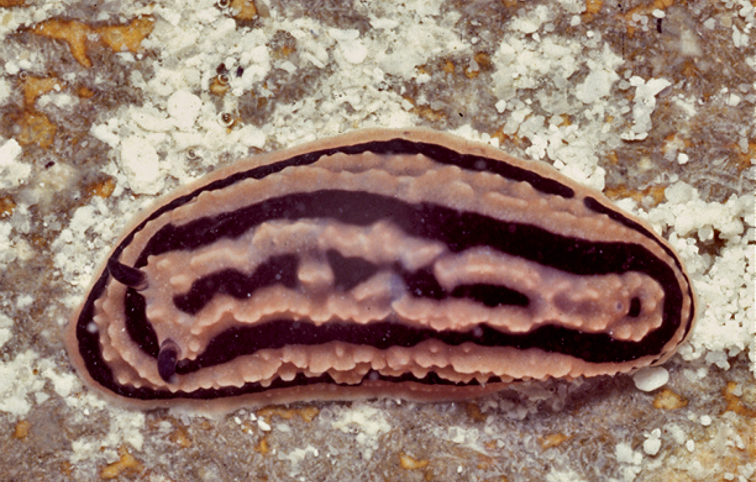
*Phyllidiella zeylanica*, Maldives, photo: J Hinterkircher (no spcm).

**Plate 73.**
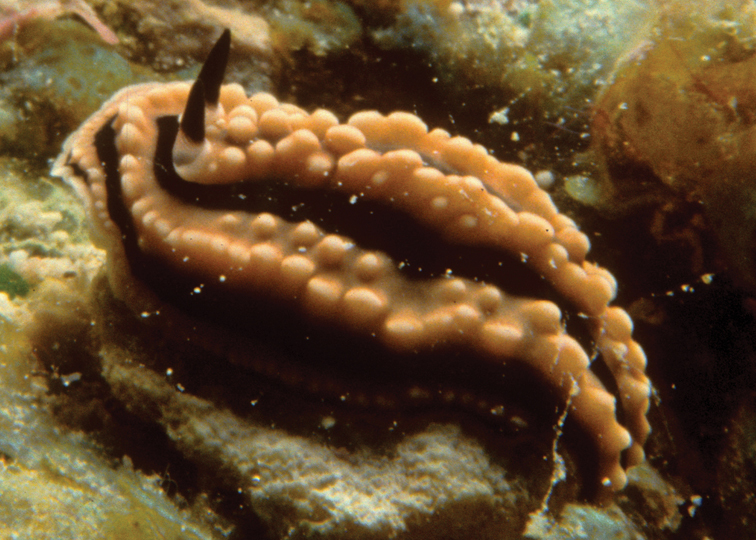
*Phyllidiella zeylanica*, 38 mm, Maldives, photo: H Debelius (spcm in Yonow 1996).

**Plate 74.**
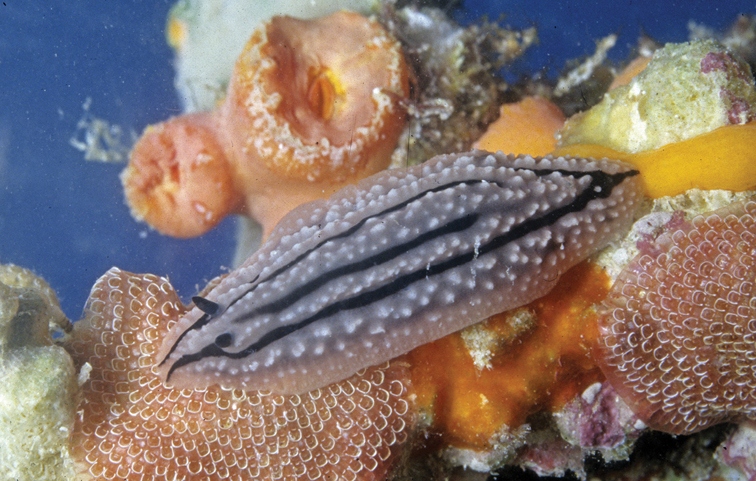
*Phyllidiopsis gemmata*, Maldives, photo: J Hinterkircher (no spcm).

**Plate 75.**
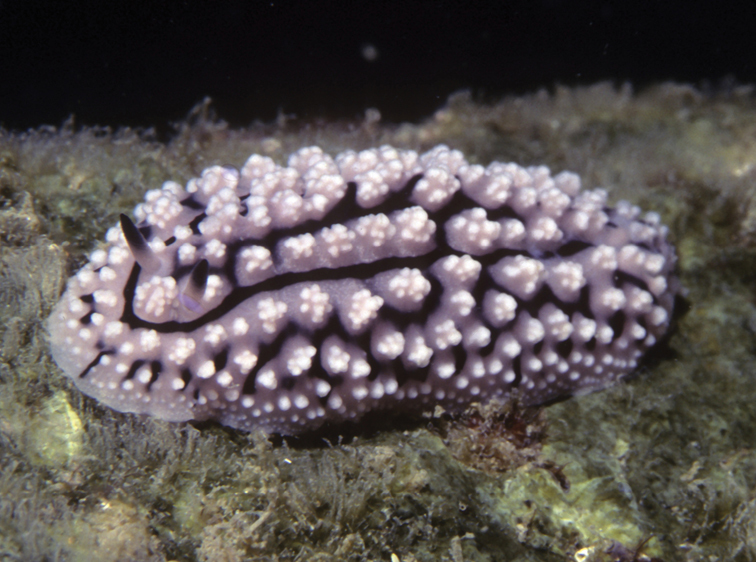
*Phyllidiopsis krempfi*, 40 mm pres., Sri Lanka, photo: RC Anderson.

**Plate 79.**
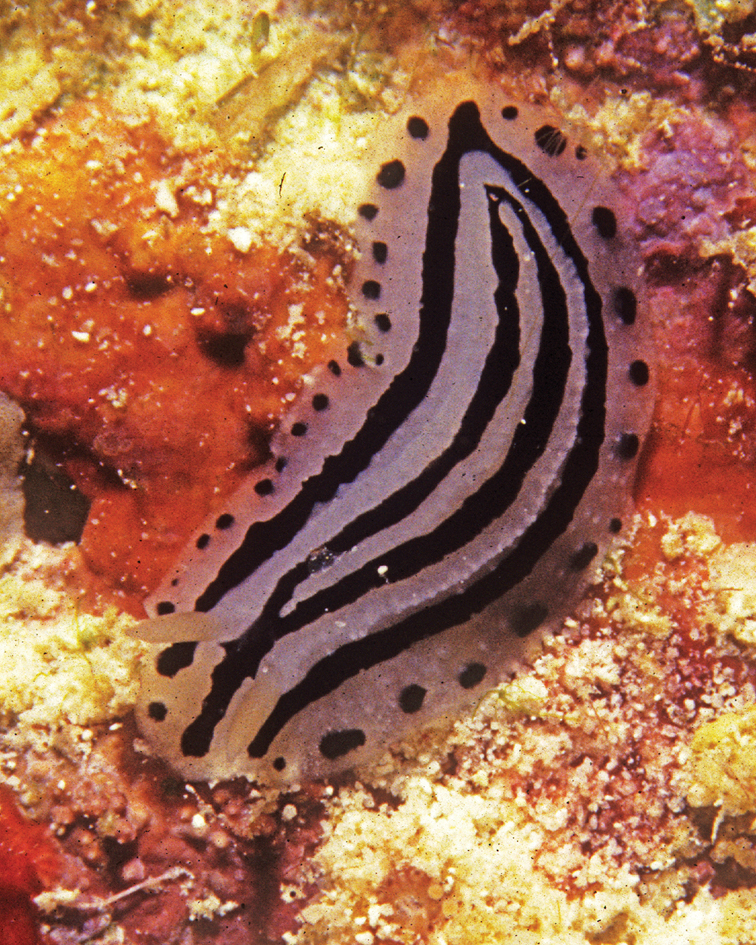
*Phyllidiopsis xishaensis*, 10 mm, Maldives, photo: RC Anderson.

## Appendix

List of species recorded in this paper with locality data for each. (doi: doi: 10.3897/zookeys.197.1728.app). File format: Excel spreadsheet (xls).

**Explanation note:** List of species recorded in this paper with locality data for each; the total number of species for each locality is listed along the last row. References of previous records are listed and X denotes species recorded in this work. All references are included in the synonymy and reference list.
